# Encoding of contextual fear memory in hippocampal–amygdala circuit

**DOI:** 10.1038/s41467-020-15121-2

**Published:** 2020-03-13

**Authors:** Woong Bin Kim, Jun-Hyeong Cho

**Affiliations:** Department of Molecular, Cell and Systems Biology, University of California, Riverside, CA 92521 USA

**Keywords:** Learning and memory, Neural circuits, Synaptic plasticity

## Abstract

In contextual fear conditioning, experimental subjects learn to associate a neutral context with an aversive stimulus and display fear responses to a context that predicts danger. Although the hippocampal–amygdala pathway has been implicated in the retrieval of contextual fear memory, the mechanism by which fear memory is encoded in this circuit has not been investigated. Here, we show that activity in the ventral CA1 (vCA1) hippocampal projections to the basal amygdala (BA), paired with aversive stimuli, contributes to encoding conditioned fear memory. Contextual fear conditioning induced selective strengthening of a subset of vCA1–BA synapses, which was prevented under anisomycin-induced retrograde amnesia. Moreover, a subpopulation of BA neurons receives stronger monosynaptic inputs from context-responding vCA1 neurons, whose activity was required for contextual fear learning and synaptic potentiation in the vCA1–BA pathway. Our study suggests that synaptic strengthening of vCA1 inputs conveying contextual information to a subset of BA neurons contributes to encoding adaptive fear memory for the threat-predictive context.

## Abstract (web summary)

Previous studies implicate the hippocampal–amygdala pathway in contextual fear conditioning, in which animals learn to associate a neutral context with an aversive stimulus and display fear responses to dangerous situations. Here the authors show that selective strengthening of hippocampal–amygdala pathway contributes to encoding adaptive fear memory for threat-predictive context.

## Introduction

In order to survive, animals develop fear responses to dangerous situations. The neural mechanism of learned fear has great survival value for animals, which must predict danger from seemingly neutral contexts. In contextual fear conditioning, an experimental model of fear learning, experimental subjects learn to associate a neutral context with an aversive stimulus and display fear responses to a context that predicts danger^1^. Contextual fear learning requires coordinated activity of the hippocampus and amygdala^2^. Ventral CA1 (vCA1) hippocampal neurons encode and convey contextual representations through monosynaptic projections to the amygdala, which induces defensive behavior^3–5^. Thus, the vCA1–amygdala pathway can play an essential role in contextual fear learning. Although the vCA1–amygdala pathway has been implicated in the retrieval of contextual fear memory^4^, the mechanism by which contextual fear memory is encoded in this circuit has not been investigated.

Exposure to a context activates a subset of vCA1 hippocampal neurons, which convey contextual representations directly to the amygdala^3^. The contextual information is then integrated with aversive signals in the amygdala for fear memory formation^1,2^. Strengthening of the hippocampal–amygdala pathway as a consequence of learning can facilitate the activation of the amygdala, resulting in conditioned fear responses to the threat-predictive context during the recall of contextual fear memory^6^. Moreover, selective strengthening of the hippocampal inputs conveying specific contextual information to the amygdala can confer selective fear responses only to the relevant context^7^. However, these hypotheses have not been examined in contextual fear conditioning. Recent studies have identified memory engram cells in the hippocampus and amygdala^8–13^. Although these studies demonstrate the role of memory engram cells in contextual fear learning, it remains unknown how memory engram cells in the amygdala are connected to hippocampal engram cells encoding specific contextual representations, as well as how the synaptic strength of these connections is modified to encode contextual fear memory. In this study, we determined the mechanism by which contextual fear memory is encoded in the hippocampal–amygdala circuit by testing our hypothesis that fear memory associated with a particular context is encoded by selective strengthening of hippocampal inputs conveying the contextual information to the amygdala.

## Results

### vCA1–BA activity contributes to contextual fear learning

**Fig. 1:**
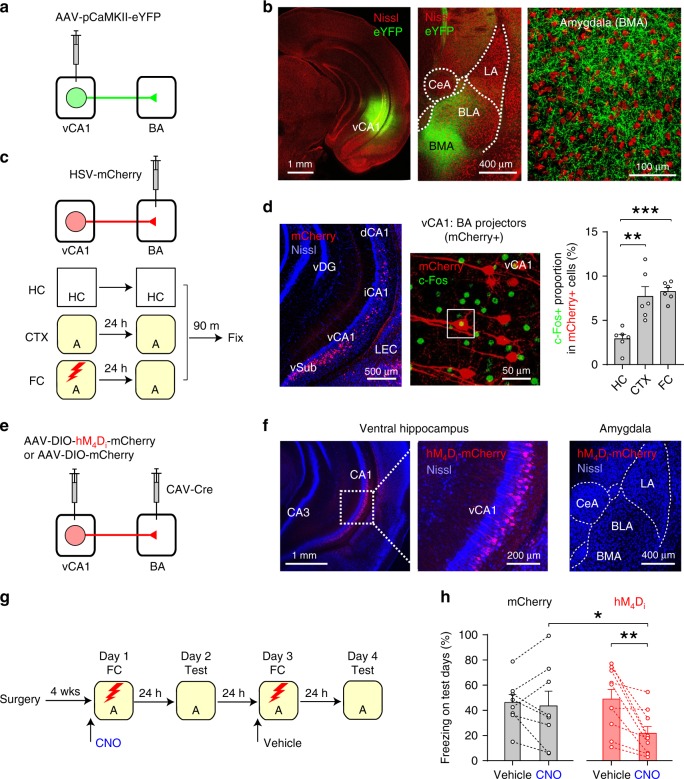
Activity in the vCA1–BA pathway contributes to the acquisition of contextual fear memory. **a** Experimental setup for **b**. **b** Images showing eYFP expression in the vCA1 (left, green) and eYFP-labeled vCA1 axons in the amygdala (middle and right). Red, Nissl stain. LA, BLA, BMA, and CeA: lateral, basolateral, basomedial, and central nuclei of the amygdala, respectively. **c** Experimental setup for **d**. Top: vCA1 neurons projecting to the BA (vCA1: BA projectors) were retrogradely labeled with HSV-mCherry. Bottom: mice in FC group were fear conditioned in Context A as in Supplementary Fig. 1a. Mice in CTX group were exposed to Context A without a US. After 24 h, they were tested for freezing behavior in Context A. Brain tissues were then fixed 90 min later for c-Fos immunohistochemistry. Mice in HC group were left in their home cages until brain fixation. **d** Left: image showing vCA1: BA projectors (red) in the dorsal (dCA1), intermediate (iCA1), ventral CA1 hippocampus (vCA1), and ventral subiculum (vSub). vDG, ventral dentate gyrus. LEC, lateral entorhinal cortex. Middle: image showing c-Fos+ cells (green) and vCA1: BA projectors (mCherry+, red). A square indicates a c-Fos+ vCA1: BA projector. Right: quantification of c-Fos+ proportion among vCA1: BA projectors (6 mice per group). **p = 0.001, ***p < 0.001 (one-way ANOVA with post hoc comparisons). **e** Experimental setup for **f**–**h**. vCA1: BA projectors expressed hM_4_D_i_-mCherry or mCherry. We bilaterally injected retrograde CAV2-Cre into the BA and AAV-DIO-hM4Di-mCherry (hM4Di group) or AAV-DIO-mCherry (mCherry group) into the vCA1. **f** Images showing hM_4_D_i_-mCherry expression in vCA1: BA projectors (left and middle). Note no hM_4_D_i_-mCherry expression in the amygdala (right). **g** Behavioral training and testing protocols for **h**. **h** Quantification of freezing behavior on test days in the hM_4_D_i_ (left, 10 mice) and mCherry groups (right, 8 mice) on test days. *p < 0.05, **p < 0.01 (two-way ANOVA with post hoc comparisons; group × treatment interaction, *p* < 0.05). Error bars indicate standard error of the mean (SEM). Source data are provided as a Source Data file. See also Supplementary Figs. 1 and 2.

In the anterograde tracing experiment, eYFP-labeled vCA1 projections were found in the basolateral (BLA) and basomedial nuclei of the amygdala (BMA), collectively termed the basal amygdala (BA) (Fig. 1a, b). In the retrograde tracing experiment, hippocampal neurons projecting to the BA were predominantly found in the vCA1 and ventral subiculum (Fig. 1c, d), suggesting monosynaptic connection of vCA1 neurons to BA neurons^3^. More vCA1 neurons projecting to the BA (vCA1:BA projectors) expressed the immediate early gene *c-fos* in mice that were exposed to a novel context or recalled contextual fear memory than in mice left in their home cages (Fig. 1c, d, Supplementary Fig. 1a, and Supplementary Table 1), suggesting that a subset of vCA1: BA projectors can encode contextual representations. We next determined the role of the vCA1–BA pathway in the formation of contextual fear memory using a chemogenetic approach (Fig. 1e, f). Application of clozapine N-oxide (CNO) induced hyperpolarization and inhibited action potential (AP) firing in vCA1: BA projectors expressing hM_4_D_i_ (Supplementary Fig. 2a–c), indicating the validity of our approach to silence vCA1–BA activity. After surgery, mice received a CNO injection and received unconditioned stimuli (US) in Context A 30 min later (Fig. 1g and Supplementary Fig. 1a). After 24 h, mice were tested for freezing behavior in Context A. On the following day, mice were fear conditioned in Context A after a vehicle injection and tested for freezing behavior in Context A 24 h later. In the hM_4_D_i_ group, mice displayed significantly reduced freezing behavior when they had received a CNO injection on the training day compared with a vehicle injection, whereas in the mCherry group, there was no difference in freezing behavior on the test days between CNO and vehicle injections on the training day (Fig. 1h). The CNO effect in the hM_4_D_i_ group on conditioned fear response was not due to the order of CNO and vehicle injections before fear conditioning (Supplementary Fig. 2d–e). These results indicate that silencing vCA1–BA activity during contextual fear learning decreased conditioned fear responses to the context 24 h later. Thus, vCA1–BA activity contributes to the acquisition of contextual fear memory^14^.

### vCA1–BA activity paired with shocks generated a fear memory

**Fig. 2:**
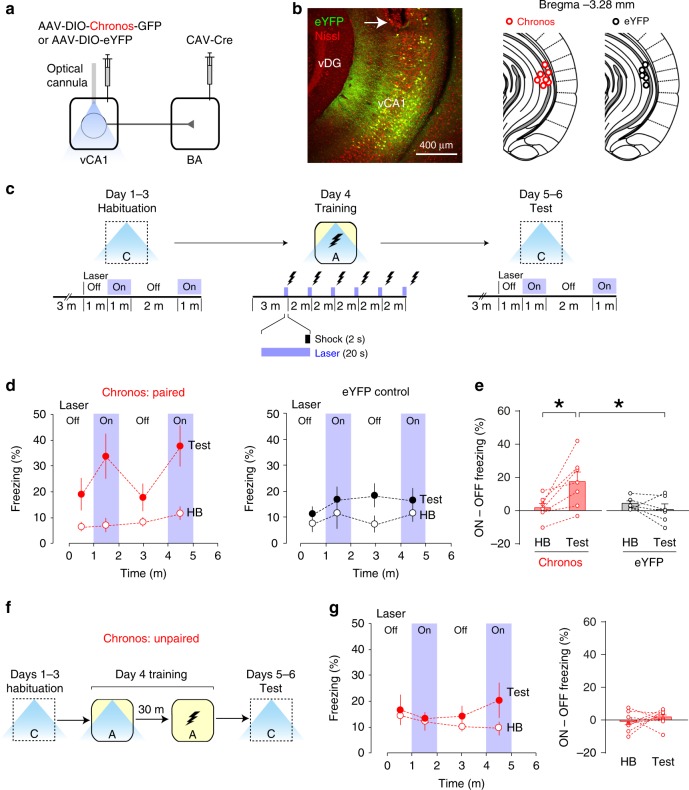
The activation of vCA1 neurons projecting to the BA, paired with aversive stimuli, generated a new conditioned fear memory. **a** An optical cannula was implanted to photostimulate vCA1 neurons projecting to the BA (vCA1: BA projectors) expressing Chronos-GFP (Chronos group) or eYFP (eYFP group). Retrograde CAV2-Cre was injected into the BA, and AAV-DIO-Chronos-GFP or AAV-DIO-eYFP was injected into the vCA1. **b** Left: image showing eYFP expression (green) in vCA1 neurons and optical cannula tip (arrow). Right: diagrams showing optical cannula implantation sites in **d** and **e**. **c** Experimental setup for **d**, **e**. Mice were habituated to 20 Hz photostimulation in Context C on days 1–3. After a 3-min acclimatization period and baseline recording of freezing behavior for 1 min, 20 Hz photostimulation was applied to the vCA1 through an optical cannulae (blue). On day 4, the mice received 20 Hz photostimulation (blue bars) 6 times, each co-terminating with a footshock, in Context A. On days 5–6, the mice were tested for freezing behavior in Context C in the presence and absence of 20 Hz photostimulation. **d** The time course of freezing behavior in Context C during habituation (HB) and test sessions in the Chronos (7 mice) and eYFP groups (6 mice). Freezing time on Days 1–3 (HB) and Days 5–6 (test) were averaged in each mouse for each time bin. **e** Summary plot showing the difference in the average freezing time in the presence and absence of photostimulation (ON – OFF freezing) during habituation and test sessions. *p < 0.05 (two-way ANOVA with post hoc comparisons, group × behavioral session interaction, *p* < 0.01). **f** Experimental setup for **g**. Mice in the Chronos: unpaired group underwent surgery with AAV-DIO-Chronos-GFP injected into the vCA1 as in **a**. On the training day, the mice received 20 Hz photostimulation 6 times in Context A and then received 6 shocks in Context A 30 min later. The mice were tested for photostimulation-induced freezing behavior in Context C. **g** Left: the time course of freezing behavior during habituation and test sessions in the Chronos: unpaired group (9 mice). Right: summary plot of the difference in the average freezing time in the presence and absence of photostimulation (ON – OFF freezing). *p* = 0.39, habituation vs. test sessions (two-sided paired *t*-test). Error bars represent the SEM. Source data are provided as a Source Data file. See also Supplementary Fig. 3.

We next examined whether the activation of a random population of vCA1: BA projectors could serve as a conditioned stimulus with which mice could learn to associate an aversive stimulus. We induced Chronos or eYFP expression in vCA1: BA projectors and implanted an optical cannula to the vCA1 for in vivo photostimulation (Fig. 2a, b). Blue light illumination at 20 Hz reliably induced AP firing in Chronos-expressing vCA1 neurons (Supplementary Fig. 3). After surgery, mice were placed in Context C for habituation to photostimulations, which did not induce freezing behavior (Fig. 2c, d and Supplementary Table 2). On the training day, the mice received 20 Hz photostimulation co-terminating with the US in Context A. On the test days, the mice received the same 20 Hz photostimulation in Context C. In the Chronos group, photostimulation during test sessions significantly increased freezing behavior than photostimulation during habituation, whereas such an effect was not observed in the eYFP group (Fig. 2d, e). However, when presented unpaired with the US on the training day, photostimulation on the test days did not increase freezing behavior in the Chronos group (Fig. 2f, g), suggesting that photostimulation-induced freezing behavior requires temporal association of photostimulation with the US on the training day. Together, our results indicate that the activation of vCA1: BA projectors induced freezing behavior after their activation was temporally paired with the US.

### Functional labeling of vCA1 neurons active in a context

**Fig. 3:**
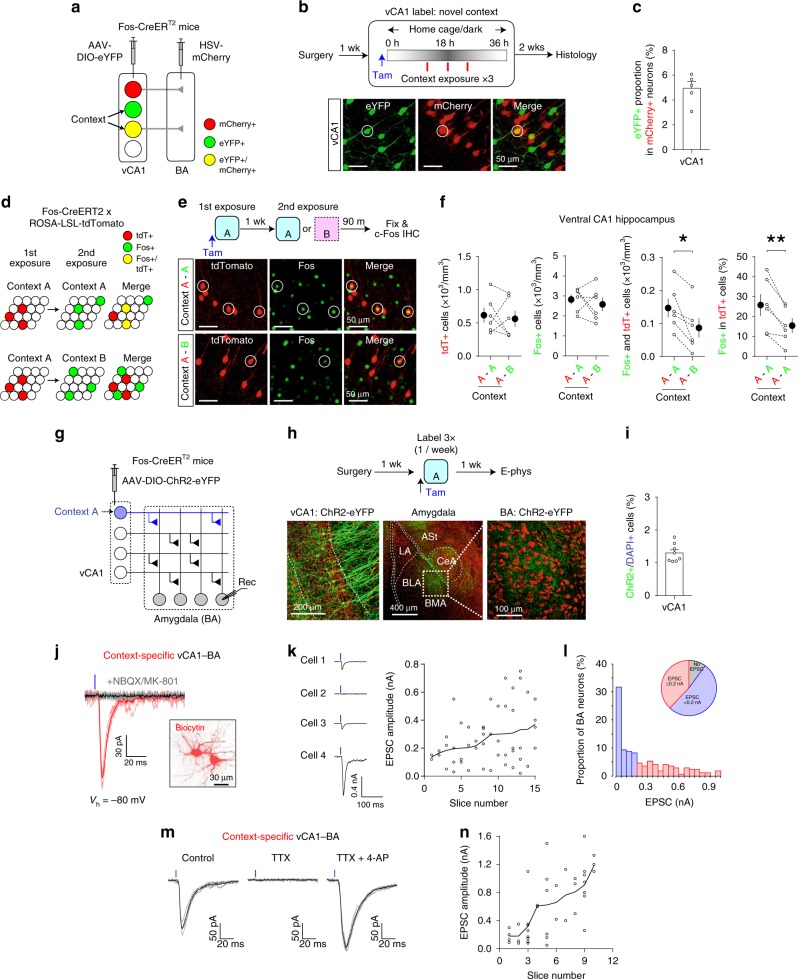
Activity-dependent functional labeling identified vCA1 neurons that are active in a new context and project to a subset of BA neurons. **a** Experimental setup for **b** and **c**. vCA1 neurons projecting to the BA were labeled with mCherry, whereas vCA1 neurons active in a novel context were labeled with eYFP in Fos-CreER^T2^ mice. **b** Top: mice were exposed to a novel context (red vertical bars) after tamoxifen injection (Tam). Bottom: magnified images of the vCA1. A vCA1 neuron labeled with both eYFP and mCherry is circled. **c** The proportion of eYFP-labeled neurons among all mCherry+ vCA1 neurons. *n* = 5 mice. **d** Experimental setup for **e** and **f**. **e** Top: after tamoxifen injection, mice were exposed to Context A to label with tdTomato vCA1 neurons active in Context A. Mice were then exposed to Context A (A–A group) or Context B (A–B group) before brain fixation for c-Fos immunostaining (IHC). Middle and bottom: images showing vCA1 neurons labeled with tdTomato (red) or c-Fos (green). vCA1 neurons labeled with both tdTomato and c-Fos are circled. **f** Comparisons of tdTomato+ cell density (*p* = 0.69), c-Fos+ cell density (*p* = 0.53), Fos+ and tdTomato+ cell density (*p = 0.015), and c-Fos+ proportion among all tdTomato+ vCA1 neurons (**p = 0.007). *n* = 6 mice per group. Two-sided paired *t*-tests were used. **g** Experimental setup for **h**–**n**. Horizontal lines indicate the axons of vCA1 neurons, and vertical lines indicate the dendrites of BA neurons. vCA1 neurons active in Context A expressed ChR2-eYFP (blue). Photostimulation activated ChR2-expressing axons and induced postsynaptic responses recorded in BA neurons (Rec). **h** Top: after surgery, mice received a tamoxifen injection and were exposed to Context A as in **b** to induce ChR2-eYFP expression in vCA1 neurons active in Context A. They received three context labeling sessions with a 1-week interval. Bottom: images showing ChR2-eYFP+ vCA1 neurons (green) and their axons in the amygdala. Red, Nissl stain. **i** The proportion of ChR2-eYFP+ cells among all DAPI+ vCA1 neurons. *n* = 8 mice. **j** Representative traces of EPSCs induced by photostimulation of ChR2+ vCA1 axons and recorded at –80 mV in voltage-clamp mode in a BA principal neuron (red). EPSCs were inhibited by NBQX and MK-801 (black). Inset: image of BA neurons loaded with biocytin during recording and labeled with streptavidin-Alexa Fluor 633. **k** Left: EPSCs recorded in four BA neurons in a brain slice and induced by photostimulation of the same intensity. Right: scatter plot of the peak amplitudes of EPSCs recorded in BA neurons in each brain slice (*n* = 15 slices). Open circles indicate EPSC amplitudes in individual BA neurons. The average amplitude of EPSCs recorded in BA neurons in the same brain slice (black curve) was used to sort data along the *x*-axis in increasing order. **l** Histogram showing the distribution of the peak amplitudes of EPSCs induced by photostimulation of the same intensity (20.0 mW/mm^2^). *n* = 190 BA neurons. **m** Tetrodotoxin (TTX, 1 μM) completely blocked EPSCs in the Context A vCA1–BA pathway (left and middle). Subsequent application of 4-aminopyridine (4-AP, 1 mM) in the presence of TTX rescued EPSCs (right), indicating the monosynaptic nature of EPSCs. **n** Scatter plot of the peak amplitudes of monosynaptic EPSCs induced by photostimulation of the same intensity and recorded in BA neurons in each brain slice as in **m**. Open circles indicate EPSC amplitudes in each BA neurons. The average amplitude of EPSCs recorded in BA neurons in the same brain slice (black curve) was used to sort data along the x-axis in increasing order. Error bars represent the SEM. Source data are provided as a Source Data file. See also Supplementary Figs. 4–7.

We next employed a neural activity-dependent labeling approach with heterozygous Fos-CreER^T2^ knock-in mice^15^ to label vCA1 neurons active in a context. We injected AAV-DIO-eYFP into the vCA1 and retrograde HSV-mCherry into the BA in these mice (Fig. 3a). We exposed the mice to a novel Context A for 12 min 14, 19, and 24 h after tamoxifen administration (Fig. 3b). vCA1 neurons active in Context A expressed CreER^T2^ under the control of endogenous c-Fos promoter, which then induced the recombination of the DIO in the presence of tamoxifen, resulting in permanent eYFP expression, whereas vCA1 neurons projecting to the BA were retrogradely labeled with mCherry. Our labeling induced eYFP expression in 4.9 ± 0.6% of vCA1 neurons that project to the BA (mCherry+) (mean ± SEM; Fig. 3c), suggesting a small population of vCA1: BA projectors was active in Context A. eYFP-labeled neurons were found predominantly in the vCA1 than in other subregions of the hippocampus and were not detected in adjacent brain areas (Supplementary Fig. 4a–d). Without tamoxifen injection before context exposure, transgene expression was not detected in the vCA1 (Supplementary Fig. 5a). We confirmed the specificity of our labeling with c-Fos immunohistochemistry in Fos-CreER^T2^ × ROSA-LSL-tdTomato mice (Fig. 3d). The c-Fos+ proportion among all tdTomato-labeled vCA1 neurons as well as c-Fos+ and tdTomato+ cell density was significantly higher in mice exposed to the same context (A–A group) than in mice exposed to different contexts (A–B group), whereas there was no significant difference in tdTomato+ or c-Fos+ cell density (Fig. 3e, f and Supplementary Fig. 4e–g). In a separate experiment, more vCA1 neurons were labeled with tdTomato in mice exposed to a novel context than in mice that remained in the home cages after tamoxifen injection (Supplementary Fig. 5b), indicating that a subset of labeled neurons reflected vCA1 neurons active during context exposure. Moreover, a subset of labeled vCA1 neurons stably encoded the context representations over time (Supplementary Fig. 5c–e). Together, these results indicate that Context A exposure after tamoxifen injection induces labeling of a vCA1 neuronal population in Fos-CreER^T2^ mice. As a subset of labeled neurons reflects vCA1 neurons active in Context A, we termed these labeled neurons ‘Context A vCA1 neurons’.

We next examined how each BA neuron received inputs from vCA1 neurons active in a context. We labeled vCA1 neurons active in Context A with ChR2-eYFP three times with a 1-week interval for sufficient ChR2 expression (Fig. 3g, h). Three context labeling sessions induced transgene expression in more vCA1 neurons than one labeling session did, whereas the number of labeling sessions did not affect the context specificity (Supplementary Fig. 5c–e). After labeling, ChR2-eYFP expression was detected in 1.3 ± 0.1% of vCA1 neurons (mean ± SEM, 8 mice), and eYFP-labeled axons were sparsely distributed in the BA (Fig. 3h, i). In brain slices, blue light illumination in the BA selectively activated axons of ChR2-expressing Context A vCA1 neurons, which we termed ‘Context A vCA1 inputs’. Thus, photostimulation in the BA induced synaptic responses in the Context A vCA1 inputs to BA pathway, which we termed ‘Context A vCA1–BA pathway’. Using whole-cell patch-clamp technique, we recorded in BA principal neurons excitatory postsynaptic currents (EPSCs), which were mediated by glutamate, an excitatory neurotransmitter (Fig. 3j). EPSC amplitude and the proportion of BA neurons that displayed EPSCs were proportional to the number of context labeling sessions (Supplementary Fig. 6). The peak amplitudes of EPSCs recorded in different BA neurons in the same brain slice were heterogeneous (95% confidence interval: −0.08–0.76 nA, Fig. 3k), and robust EPSCs were detected only in a subset of BA neurons (Fig. 3l). We isolated monosynaptic EPSCs in the Context A vCA1–BA pathway, and their amplitude was also heterogeneous among BA neurons (Fig. 3m, n). However, when vCA1 neurons globally expressed ChR2 and their axons were randomly stimulated, the EPSC amplitudes were much larger and less variable (95% confidence interval: 1.5–8.7 nA, Supplementary Fig. 7a–e). These results suggest that a subset of BA neurons receive more vCA1 inputs conveying specific contextual information than other BA neuronal populations do (Supplementary Fig. 7f–i).

### Contextual fear learning strengthened vCA1 inputs to the BA

**Fig. 4:**
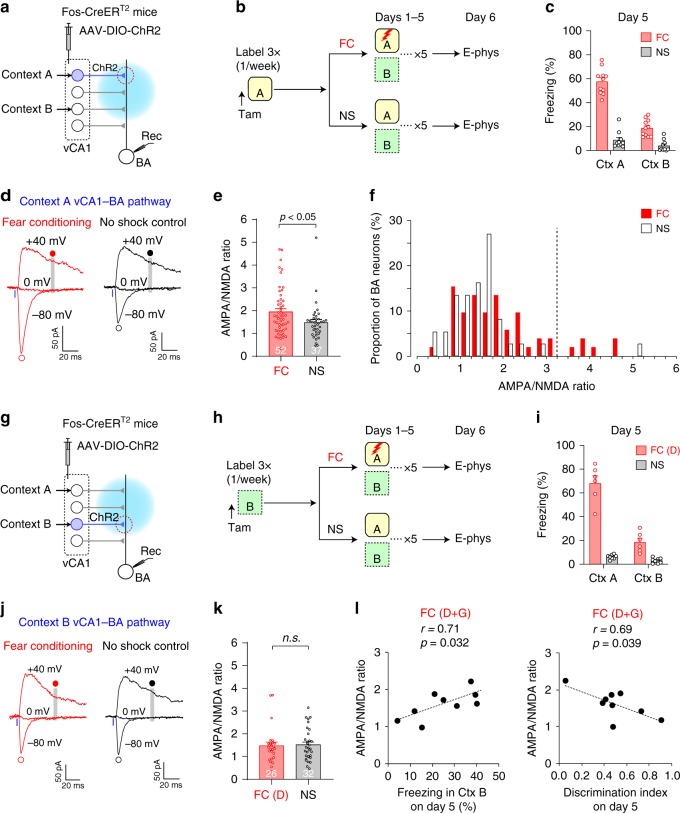
Discriminative contextual fear learning induced synaptic strengthening in the vCA1 inputs that convey threat-predictive contextual information to the BA. **a** Experimental setup for recording synaptic responses in the Context A vCA1–BA pathway in **b**–**f**. ChR2 was expressed in vCA1 neurons active in Context A. Photostimulation selectively activated Context A vCA1 inputs and induced postsynaptic responses in BA neurons (Rec). **b** Mice were exposed to Context A for labeling vCA1 neurons active in Contest A as in Fig. 3h. Mice in the fear conditioning (FC) group were trained for discriminative fear in Context A on Days 1–5 as in Supplementary Fig. 1b. Mice in the no shock (NS) control group were exposed to the contexts without a shock. **c** Freezing behavior in Contexts (Ctx) A and B on Day 5 in the FC (11 mice) and NS groups (10 mice). **d** Traces of EPSCs induced by blue light (1 ms pulses, blue bars), which activated ChR2-expressing Context A vCA1 inputs. EPSCs were recorded at −80 mV, 0 mV, and +40 mV in voltage-clamp mode in the same BA neurons. AMPAR EPSCs were quantified as the peak amplitude of EPSCs recorded at −80 mV (open circles). NMDAR EPSCs were quantified as the average amplitude of EPSC recorded at +40 mV from 47.5 ms to 52.5 ms after photostimulation onset (gray vertical lines and closed circles). SR-95531 (10 μM) was added to block inhibitory postsynaptic currents. **e** Comparison of the AMPA/NMDA ratio in Context A-specific vCA1–BA pathway between the FC and NS groups (*p* = 0.024). Two-sided Kruskal Wallis multiple comparisons were used to analyze combined data in **e** and **k**. Open circles indicate the AMPA/NMDA ratio in each neuron. Numbers within the bars are the number of neurons examined in each group. **f** Histogram showing the distribution of the AMPA/NMDA EPSC ratio in Context A inputs to each BA neuron in the FC (red bars, 52 cells) and NS groups (open bars, 37 cells). A dotted vertical line indicates the mean + 2 standard deviations of the AMPA/NMDA ratio in the NS group. **g** Experimental setup for recording synaptic responses in the Context B vCA1–BA pathway in **h**–**l**. ChR2 was expressed in vCA1 neurons active in Context B. Photostimulation activated Context B vCA1 inputs and induced postsynaptic responses in BA neurons. **h** Mice were exposed to Context B for labeling vCA1 neurons. Mice in the FC group were trained for discriminative fear in Context A on Days 1–5 as in Supplementary Fig. 1b, whereas mice in NS group were exposed to the contexts without a shock. **i** Freezing behavior in Contexts A and B on Day 5 in the FC (6 mice) and NS groups (8 mice). In the FC group, only discriminators (D, freezing score in Context B on Day 5 <35%) were included in the analysis in **j**–**k**. **j** *Left*: traces of EPSCs recorded in the Context B pathway. AMPAR and NMDAR EPSCs were induced and recorded as in **d**. **k** Comparison of the AMPA/NMDA ratio in Context B vCA1–BA pathway between the FC and NS groups (*n.s*., not significant; *p* = 0.68, two-sided Kruskal Wallis multiple comparisons). **l** Correlation between the average AMPA/NMDA ratio vs. freezing score in Context B on Day 5 (Pearson correlation coefficient *r* = 0.71, left) and discrimination index [DI = (Context A freezing − Context B freezing)/(Context A freezing + Context B freezing); Pearson correlation coefficient *r* = 0.69; right]. Both discriminators (D) and generalizers (G, freezing score in Context B on Day 5 >35%) in the FC group were included. For each mouse, the AMPA/NMDA ratios in 4–5 BA neurons were averaged. Error bars represent the SEM. Source data are provided as a Source Data file. See also Supplementary Figs. 8–11.

We next examined how associative fear learning for a particular context affected synaptic strength in vCA1 inputs conveying the contextual information to the BA. We labeled vCA1 neurons active in Context A with ChR2 (Fig. 4a, b). We then trained mice learn to discriminate between contexts and display freezing behavior preferentially in a context that predicts danger. Mice in the fear conditioning (FC) group received a shock in Context A but not in Context B (Fig. 4b and Supplementary Fig. 1b). After multiple trials of fear conditioning, the mice showed fear responses predominantly in Context A (Fig. 4c and Supplementary Fig. 1c–d). Mice in the no shock (NS) control group were exposed to the contexts without the US and did not show fear responses in Context A or B (Fig. 4b, c and Supplementary Fig. 1c). In brain slices, we photostimulated Context A vCA1 inputs and recorded EPSCs in BA principal neurons, which were differentiated from GABAergic interneurons based on their intrinsic membrane properties (Supplementary Fig. 8). Photostimulations induced EPSCs, which reflected postsynaptic responses in the Context A vCA1–BA pathway (Fig. 4a, d). To detect changes in synaptic strength by postsynaptic mechanisms, we recorded both AMPA receptor (AMPAR)- and NMDA receptor (NMDAR)-mediated EPSCs in the same BA neurons and calculated the AMPA/NMDA ratio^16^, which was significantly higher in the FC group than in the NS group (Fig. 4d, e, Supplementary Table 3), suggesting synaptic potentiation in the Context A pathway after fear learning in Context A. However, only 11.5% of 52 BA neurons in the FC group displayed the AMPA/NMDA ratio larger than the average ratio in the NS group by more than two standard deviations (Fig. 4f), suggesting that only a small population of BA neurons underwent synaptic strengthening in the Context A pathway. To detect synaptic changes by presynaptic mechanisms, we compared the rate of progressive block of NMDAR EPSCs by MK-801 and the paired-pulse ratio (PPR)^17,18^, which were not significantly different between groups (Supplementary Fig. 9a–b, 9d; Supplementary Table 4). In the Context A vCA1 inputs to the CeA, we did not detect significant difference in the AMPA/NMDA ratio between groups (Supplementary Fig. 10), suggesting that synaptic changes associated with contextual fear learning are pathway-specific.

We next examined whether discriminative fear conditioning in Context A altered the strength of the Context B vCA1–BA synapses (Fig. 4g). After labeling vCA1 neurons active in Context B with ChR2, mice in the FC group underwent discriminative fear conditioning in Context A (Fig. 4h, i). Some mice (i.e., discriminators) displayed freezing behavior predominantly in Context A, while others (i.e., generalizers) showed significant freezing behavior in Context B as well (>35% freezing time on Day 5). Because the purpose of this experiment was to examine how ‘discriminative’ fear conditioning in Context A affected synaptic strength in Context B vCA1–BA pathway, only discriminators in the FC groups were included in our analysis. Mice in the NS control group were exposed to the contexts without the US and did not show fear responses in Context A or B (Fig. 4h, i). In brain slices, photostimulations in the BA activated ChR2-expressing Context B vCA1 inputs and induced EPSCs, which reflected postsynaptic responses in the Context B vCA1–BA pathway. There was no difference in the AMPA/NMDA ratio (Fig. 4j, k) or the rate of progressive block of NMDAR EPSCs by MK-801 between the FC and NS groups (Supplementary Fig. 9c), indicating that synaptic efficacy in the Context B pathway did not change after fear learning in Context A. When both discriminators and generalizers were included in our analysis, the average AMPA/NMDA ratio in the Context B pathway was positively correlated with freezing score in Context B on Day 5, whereas the ratio was negatively correlated with the discrimination index (Fig. 4l). These results suggest that the synaptic strength in the Context B pathway is inversely correlated with the ability to discriminate between contexts and show selective fear responses to threat-predictive Context A.

We also induced global expression of ChR2 or Chronos in the vCA1 (Supplementary Fig. 11a–b). The AMPA/NMDA ratio was not significantly different between the FC and NS groups (Supplementary Fig. 11c). There was no significant difference between groups in PPR or the rate of progressive block of NMDAR EPSCs by MK-801 (Supplementary Fig. 11d–e). These results suggest that synaptic efficacy in the vCA1–BA pathway was not globally altered in discriminative contextual fear conditioning. Together, our results suggest that discriminative contextual fear learning selectively strengthens the vCA1–BA pathway that convey relevant contextual information to the BA.

### Fear learning strengthens a subset of vCA1–BA synapses

**Fig. 5:**
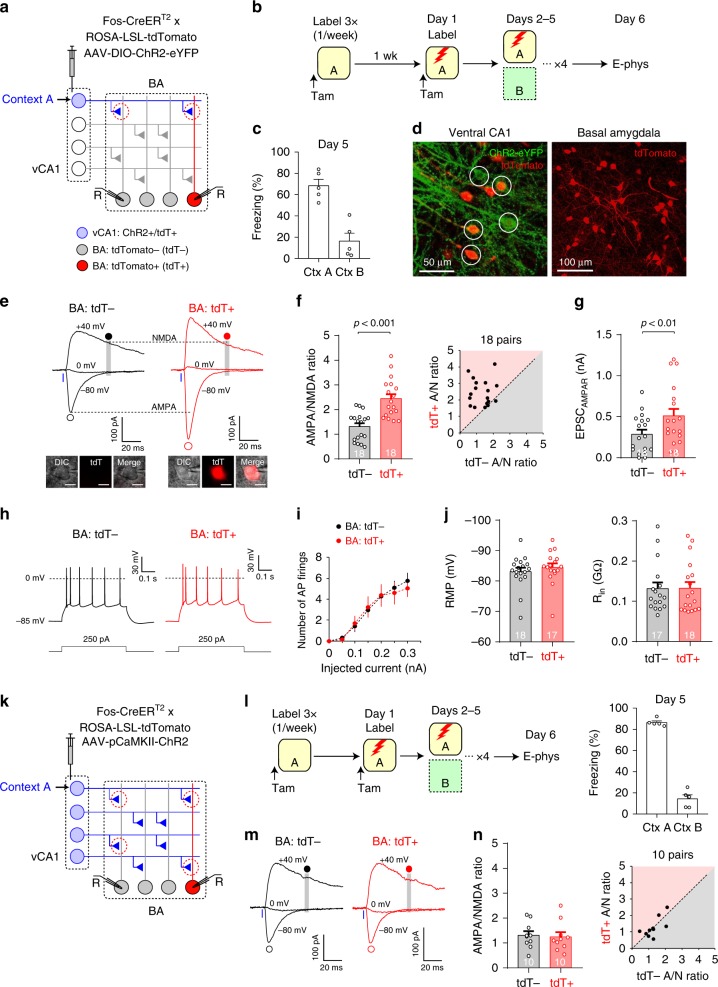
A subset of vCA1–BA synapses was selectively strengthened in discriminative contextual fear conditioning. **a** Experimental setup for **b**–**j**. **b** After surgery, mice received three context labeling sessions with a 1-week interval to induce ChR2 expression in vCA1 neurons active in Context A. After a week, the mice received tamoxifen injection and were fear-conditioned in Context A on Day 1 for tdTomato (tdT) expression in BA fear neurons. On Days 2–5, mice were trained for discriminative fear in Context A as in Supplementary Fig. 1b. **c** Comparison of freezing responses in Contexts (Ctx) A vs. Context B on Day 5 (*p* = 0.001, two-sided paired *t*-test; *n* = 5 mice). **d** Images showing ChR2-eYFP-expressing vCA1 neurons (green, circles; left) and tdT-labeled BA neurons (red; right). **e** Traces of EPSCs recorded in tdT− and tdT+ BA neurons. tdT+ neurons were identified with red fluorescence within the BA (inset; scale bar, 10 μm). EPSCs were induced with photostimulation of Context A vCA1 inputs and recorded as in Fig. 4d. **f** Left: comparison of the AMPA/NMDA (A/N) ratios between tdT− and tdT+ BA neurons. Two-way ANOVA with post hoc comparisons was used to analyze combined data in **f** and **n**. Right: scatter plot of the A/N ratios in 18 pairs of tdT− (*x*-axis) and tdT+ BA neurons (*y*-axis) that were adjacent to each other. **g** Comparison of the amplitude of AMPAR EPSC (EPSC_AMPAR_) induced by photostimulation of the same intensity (6.4 mW/mm^2^) and recorded in tdT− vs. tdT+ BA neurons (two-sided paired *t*-test). **h** Traces of AP firing induced by depolarizing current injection (500 ms long) in tdT− and tdT+ BA neurons. Baseline membrane potential was adjusted to approximate −85 mV. **i** Comparison of AP firing in tdT− (18 cells) and tdT+ BA neurons (17 cells) (*p* = 0.67, two-way ANOVA). **j** Comparison of resting membrane potential (RMP, *p* = 0.45) and input resistance (R_in_; *p* = 0.96, two-sided unpaired *t*-test) in tdT− (18 cells) and tdT+ BA neurons (17 cells). **k** Experimental setup for **l**–**n**. ChR2 was globally expressed in vCA1 neurons. BA fear neurons (tdT+) were labeled with tdT as in **b**. **l** Left: mice received three context labeling sessions and were then fear-conditioned in Context A on Day 1 for tdT expression in BA fear neurons as in **b**. On Days 2–5, the mice were trained for discriminative fear in Context A as in **b**. Right: quantification of freezing responses on Day 5. *n* = 5 mice. **m** Traces of EPSCs induced by global stimulation of vCA1 inputs and recorded in tdT− and tdT+ BA neurons as in **e**. **n** Left: comparison of the AMPA/NMDA ratios between tdT− and tdT+ BA neurons (*p* = 0.35, two-way ANOVA with post hoc comparisons). Right: scatter plot showing the AMPA/NMDA ratio in 10 pairs of tdT− and tdT+ BA neurons. Error bars represent the SEM. Source data are provided as a Source Data file. See also Supplementary Figs. 12–13.

Our results demonstrate that contextual fear learning induces vCA1 input-specific synaptic potentiation, which was detected only in a small subset of BA neurons (Fig. 4f). Synaptic plasticity for fear memory formation may be induced preferentially in synapses consisting of presynaptic vCA1 inputs and postsynaptic BA neurons that are activated during contextual fear conditioning^7,19,20^. To test this possibility, vCA1 neurons active in Context A were labeled with ChR2 during context labeling sessions, whereas BA neurons active during contextual fear conditioning were labeled with tdTomato (tdT) in Fos-CreER^T2^ × ROSA-LSL-tdTomato mice (Fig. 5a, b, d). As a subset of BA neurons labeled during contextual fear conditioning was reactivated during memory recall (see below)^11^, we termed these labeled neurons ‘BA fear neurons’. After labeling, the mice were trained for discriminative fear in Context A (Fig. 5b, c). We then induced EPSCs with photostimulation of Context A vCA1 inputs and compared EPSCs recorded in tdT-labeled BA neurons with EPSCs recorded in adjacent unlabeled neurons (Fig. 5a). Both the AMPA/NMDA ratio and AMPAR EPSC amplitudes were significantly larger in tdT+ neurons than in tdT− neurons (Fig. 5e–g and Supplementary Fig. 12a–d), whereas NMDAR EPSCs or feed-forward inhibition in the vCA1–BA pathway did not differ between these neurons (Supplementary Fig. 12e–h). However, context exposure without the US did not increase the AMPA/NMDA ratio in tdT+ neurons (Supplementary Fig. 13). Together, our results suggest that discriminative fear learning in Context A selectively strengthened Context A vCA1 inputs to BA fear neurons. We also examined whether BA fear neurons have intrinsic membrane properties that facilitate synaptic potentiation^21,22^. There was no significant difference in AP firing, resting membrane potential or input resistance between tdT+ and tdT− BA neurons (Fig. 5h–j), indicating no difference in intrinsic membrane properties between BA fear neurons and other BA neurons 5 days after initial fear conditioning.

We next examined whether discriminative fear learning also induced synaptic strengthening in randomly selected vCA1 inputs to BA fear neurons. We induced global ChR2 expression in the vCA1. For more consistent experimental conditions, the mice received three context labeling sessions as in the previous experiments although these procedures were not necessary for global ChR2 expression in the vCA1. We then labeled BA fear neurons with tdT and trained the mice for discriminative fear in Context A (Fig. 5k, l). There was no significant difference in the AMPA/NMDA ratio between tdT+ and tdT− BA neurons (Fig. 5m, n), suggesting that synapses in randomly selected vCA1 inputs to BA fear neurons were not altered in contextual fear learning. Together, these results suggest that discriminative contextual fear learning selectively strengthened synapses, which connect context-specific vCA1 neurons to BA fear neurons.

### Lack of vCA1–BA potentiation under drug-induced amnesia

**Fig. 6:**
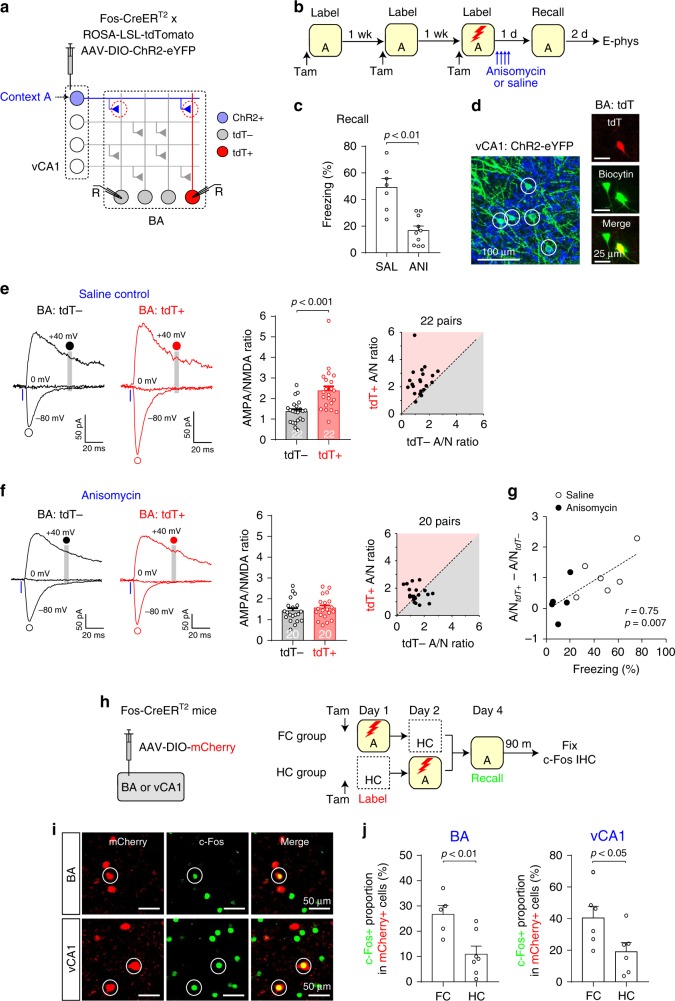
Lack of synaptic potentiation of the vCA1–BA pathway under anisomycin-induced retrograde amnesia. **a** Experimental setup for **b**–**g**. **b** Mice were exposed to Context A to induce ChR2 expression in vCA1 neurons active in Context A. They were then fear conditioned in Context A for tdT expression in BA fear neurons and received anisomycin (ANI, 10 mice) or saline injections (SAL, 7 mice). **c** Comparison of freezing behavior in Context A 24 h after fear conditioning between groups (two-sided unpaired *t*-test). **d** Left: image showing ChR2-eYFP-expressing vCA1 neurons (green, circles). Right: image of a pair of BA neurons, one tdT+ (red) and one tdT−, loaded with biocytin during recording and labeled with streptavidin-Alexa Fluor 633 (green). **e** Left: traces of EPSCs recorded in tdT− and tdT+ BA neurons in the saline control group. EPSCs were induced with photostimulation of Context A vCA1 inputs and recorded as in Fig. 4d. Middle: comparison of the AMPA/NMDA (A/N) ratios between tdT− and tdT+ neurons. Two-way ANOVA with post hoc comparisons was used to analyze combined data in **e** and **f**. Right: scatter plot of the A/N ratios in 22 pairs of tdT− and adjacent tdT+ cells in the saline control group. **f** Left: traces of EPSCs recorded in tdT− and tdT+ BA neurons in the anisomycin group. Middle and Right: comparison of AMPA/NMDA ratios between tdT− and tdT+ neurons in the anisomycin group (*p* = 1.00, two-way ANOVA with post hoc comparisons). **g** The average difference in AMPA/NMDA ratio between tdT+ and tdT− neurons in each mouse positively correlated with freezing behavior during memory recall (Pearson correlation test). **h** Experimental setup for **i** and **j**. mCherry was expressed in BA and vCA1 neurons active during contextual fear conditioning (FC group) or those active in the home cages (HC group). Neurons active during fear memory recall were immunostained for c-Fos. **i** Images showing BA and vCA1 neurons labeled with mCherry (red) and c-Fos (green). Both mCherry+ and c-Fos+ neurons were marked with circles. **j** Comparison of c-Fos+ proportion among all mCherry-labeled BA and vCA1 neurons (two-sided unpaired *t*-test). *n* = 5–7 mice per group. Error bars represent the SEM. Source data are provided as a Source Data file. See also Supplementary Figs. 14–15.

We next examined how inhibition of fear memory consolidation by anisomycin affected synaptic strength in the vCA1–BA pathway^23–25^. As in the previous experiments, we labeled vCA1 neurons active in Context A with ChR2 and BA fear neurons with tdT (Fig. 6a, b). Mice received systemic injections of anisomycin or saline after fear conditioning in Context A (Fig. 6b and Supplementary Fig. 14a). After 24 h, mice in the anisomycin group displayed less freezing behavior in Context A than mice in the saline group did (Fig. 6c), indicating anisomycin-induced retrograde amnesia. Anisomycin injection after fear conditioning decreased the density of c-Fos+ neurons in the vCA1 and BMA (Supplementary Fig. 14b–d), while it did not prevent tdT expression in the BA (Fig. 6d), suggesting that the dosage of anisomycin used in our study inhibited protein synthesis but was insufficient to completely block CreER^T2^ expression in BA fear neurons. To allow for sufficient tdT expression in BA fear neurons, we performed recording experiments 2 days after the memory recall test. In brain slices, we induced EPSCs by photostimulating Context A vCA1 inputs and compared the AMPA/NMDA ratio. In the saline group, the AMPA/NMDA ratio significantly larger in tdT+ BA neurons than in tdT− neurons (Fig. 6e), whereas there was no significant difference in the AMPA/NMDA ratio in the anisomycin group (Fig. 6f). Overall, the average difference in the AMPA/NMDA ratio between tdT+ and tdT− neurons in each mouse correlated with freezing behavior during memory recall (Fig. 6g). These results indicate that synaptic strengthening in the vCA1–BA pathway was inhibited by post-training anisomycin treatment, which also suppressed the consolidation of contextual fear memory. Moreover, the pharmacological inhibition of NMDAR during contextual fear conditioning prevented the acquisition of contextual fear memory and blocked strengthening of the vCA1–BA synapses (Supplementary Fig. 15). Together, our results suggest that the acquisition and consolidation of contextual fear memory involve synaptic potentiation in the vCA1–BA pathway.

We next examined whether vCA1 and BA neurons active during contextual fear conditioning were reactivated during fear memory recall (Fig. 6h). In the FC group, BA and vCA1 neurons active during fear conditioning were labeled with mCherry. Mice in the HC group remained in their home cages for mCherry expression in BA and vCA1 neurons active in the home cages and were then fear conditioned in Context A on the following day. On the test day, mice in both groups displayed freezing behavior in Context A. The brain tissues were fixed 90 min later for c-Fos immunostaining of neurons active during memory recall. The c-Fos+ proportion among all mCherry+ BA and vCA1 neurons was significantly higher in the FC group than in the HC group (Fig. 6i, j), indicating that BA and vCA1 neurons active during fear conditioning were more readily reactivated during memory recall than neurons active in the home cages. These results suggest that a subset of BA and vCA1 neurons active during contextual fear conditioning were reactivated during fear memory recall, thus they likely included memory engram cells in the BA and vCA1^12,13^.

### Synapse-specific encoding of fear memory in vCA1–BA circuit

**Fig. 7:**
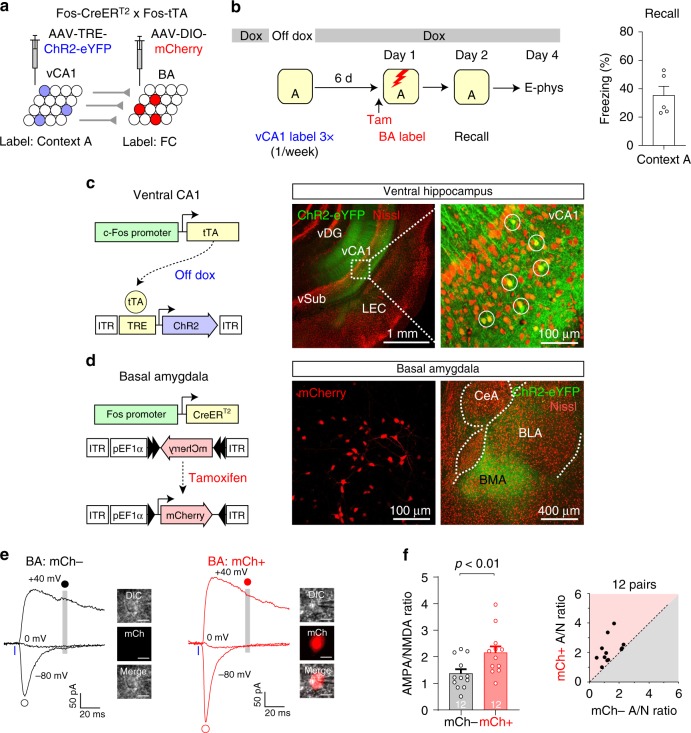
Dual independent labeling revealed selective strengthening of synapses that connect context-specific vCA1 neurons to BA fear neurons in contextual fear conditioning. **a** Experimental setup. **b** Left: mice were taken off Dox and exposed to Context A to induce ChR2 expression in Context A vCA1 neurons. Mice received two additional vCA1 labeling sessions with a 1-week interval (vCA1 label 3×). Mice were put back on Dox immediately after each context labeling session. After tamoxifen injection, the mice were fear-conditioned in Context A on Day 1 for mCherry expression in BA fear neurons (BA label). The mice were tested for freezing behavior on Day 2 (Recall). Recording experiments (E-phys) were performed on Day 4. Right: quantification of freezing responses in Context (Ctx) A on Day 2 (*n* = 5 mice). **c** Left: vCA1 neurons active in Context A express tTA under the control of c-Fos promoter, resulting in ChR2-eYFP expression in the absence of Dox. Right: images showing ChR2-eYFP-expressing vCA1 neurons (green, circles). Red, Nissl stain. **d** Left: BA neurons active during fear conditioning express CreER^T2^ under the control of c-Fos promoter, resulting in tamoxifen-dependent recombination of the DIO and subsequent mCherry expression. Right: images showing mCherry-expressing BA neurons (red, left) and eYFP-labeled vCA1 axons (green, right) in the BA. **e** Traces of EPSCs recorded in mCherry (mCh)− and mCh+ BA neurons. mCh+ neurons were identified with red fluorescence within the BA (inset; scale bar, 10 μm). EPSCs were induced with photostimulation of Context A vCA1 inputs and recorded as in Fig. 4d. **f** Left: comparison of the AMPA/NMDA (A/N) ratios between mCh− and mCh+ BA neurons (two-sided paired *t*-test). Right: scatter plot of the A/N ratios in 12 pairs of mCh− (*x*-axis) and mCh+ BA neurons (*y*-axis) that were adjacent to each other. Error bars represent the SEM. Source data are provided as a Source Data file. See also Supplementary Fig. 16.

Our labeling procedures in the previous experiments induced labeling of vCA1 and BA neurons active during contextual fear conditioning as well as those active during context exposure without the US (Figs. 5b, 6b). To avoid such a caveat, we independently labeled context-specific vCA1 neurons and BA fear neurons using Fos-CreER^T2^ × Fos-tTA double transgenic mice, in which transgene expression in the vCA1 and BA was controlled by different mechanisms. We injected AAV-TRE-ChR2-eYFP into the vCA1 and AAV-DIO-mCherry into the BA (Fig. 7a) in these mice fed with doxycycline (Dox)-containing food. After surgery, the mice were taken off Dox for 48 h and exposed to Context A, which induced the expression of tetracycline transactivator (tTA) under the control of c-Fos promoter and subsequent ChR2-eYFP expression in vCA1 neurons active in Context A^9^ (Fig. 7b, c). We confirmed the context specificity of our vCA1 labeling in Fos-tTA mice (Supplementary Fig. 16). After three context labeling sessions with a 1-week interval, the mice received a tamoxifen injection and were fear conditioned in Context A 24 h later, resulting in mCherry expression in BA fear neurons active during fear conditioning (Fig. 7b, d). The mice showed freezing behavior in Context A 24 h later (Fig. 7b). In brain slices, we photostimulated Context A vCA1 inputs and recorded EPSCs in mCherry+ and adjacent mCherry− BA neurons. The AMPA/NMDA EPSC ratio was significantly higher in mCherry+ neurons than in unlabeled BA neurons (Fig. 7e, f), indicating synaptic potentiation in Context A vCA1 inputs to BA fear neurons. Our results suggest that contextual fear learning induces selective strengthening of synapses that connect presynaptic vCA1 neurons active in threat-predictive context to postsynaptic BA fear neurons recruited during fear conditioning.

### BA fear cells receive abundant context-specific vCA1 inputs

**Fig. 8:**
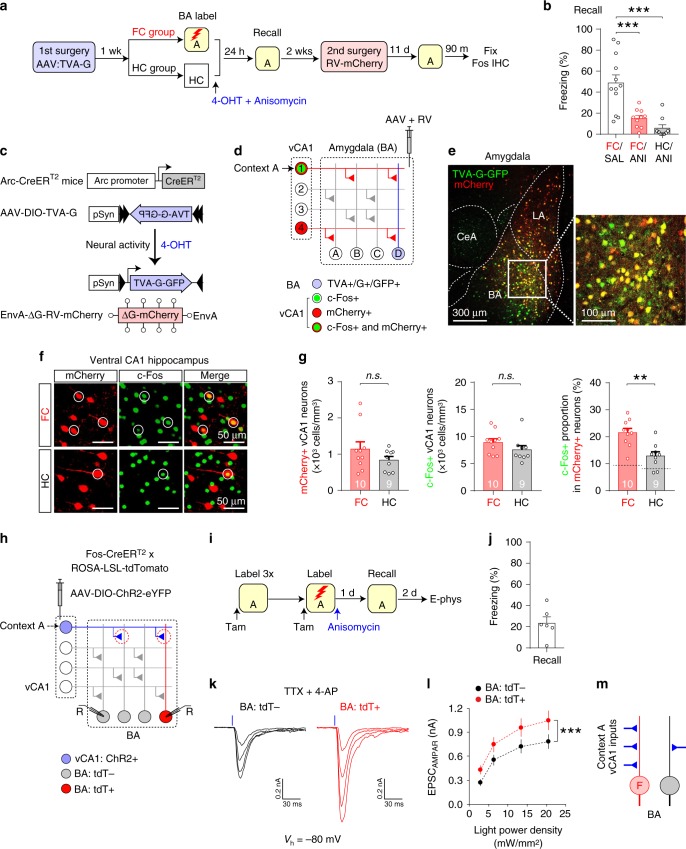
BA fear neurons recruited during fear learning receive more monosynaptic vCA1 inputs conveying threat-predictive contextual information than other BA neurons do. **a** Experimental setup for **b**–**g**. BA neurons active during fear conditioning (FC group) or those active in the home cages (HC group) were labeled with TVA-G-GFP. **b** Comparison of freezing behavior in Context A between groups 24 h after BA labeling. Mice in the FC group were fear conditioned in Context A and received saline (FC/SAL, 12 mice) or anisomycin injections (FC/ANI, 10 mice), whereas mice in the HC/ANI group remained in the home cages and received anisomycin injections (9 mice). ***p < 0.001, one-way ANOVA with post hoc comparisons. Mice in the FC/SAL group were not used for RV-mediated tracing experiments in **c**–**g**. **c** BA fear neurons in the FC group were labeled with TVA-G-GFP in a Cre-dependent manner under the control of the Arc promoter in the presence of 4-OHT in Arc-CreER^T2^ mice. EnvA-ΔG-RV-mCherry infected TVA-G-expressing BA neurons, replicated, and propagated trans-synaptically to presynaptic neurons. **d** Neural circuit diagram showing trans-synaptic labeling of vCA1 neurons projecting to BA fear neurons labeled with TVA-G in the FC group (neuron D). EnvA-ΔG-RV-mCherry infected TVA-G-labeled BA neurons, resulting in mCherry expression in vCA1 neurons (neurons 1 and 4) that projected monosynaptically to BA fear neurons. Context A-encoding vCA1 neurons were immunostained for c-Fos (neuron 1). **e** Images showing TVA-G-GFP-labeled BA neurons (green) and RV-infected BA neurons expressing mCherry (red). **f** Images showing labeled vCA1 neurons in the FC (top) and HC groups (bottom). vCA1 neurons labeled with both mCherry and c-Fos are circled. **g** Comparison of the density of mCherry+ vCA1 neurons (left, *p* = 0.19; n.s., not significant), c-Fos+ cells (middle, *p* = 0.21), and the c-Fos+ proportion among all mCherry+ vCA1 neurons (right; **p = 0.001) between the FC (10 mice) and HC groups (9 mice). Two-sided unpaired *t*-tests were used. Dotted horizontal lines indicate the probability that a randomly selected vCA1 neurons is c-Fos+. **h** Experimental setup for **i**–**l**. **i** Mice were exposed to Context A to label vCA1 neurons active in Context A with ChR2. Mice were then fear conditioned in Context A to label BA fear neurons with tdTomato (tdT) and received anisomycin injections to prevent learning-induced synaptic strengthening in the vCA1–BA pathway. **j** Freezing behavior in Context A 24 h after fear conditioning (6 mice). **k** Representative traces of monosynaptic EPSCs induced by photostimulation of Context A vCA1 inputs and recorded in tdT− and tdT+ BA neurons in the presence of TTX and 4-AP as in Fig. 3m. **l** Plot of the average amplitude of EPSCs recorded in tdT− (16 neurons) and tdT+ neurons (16 neurons) as in **k**. ***p < 0.001, repeated measures two-way ANOVA. **m** BA fear neurons (F, red) receive more vCA1 inputs conveying threat-predictive contextual representations (blue) than other BA neurons (gray) do. Error bars represent the SEM. Source data are provided as a Source Data file. See also Supplementary Figs. 17–19.

We next determined the properties of BA fear neurons that facilitate synaptic potentiation in the vCA1–BA pathway in contextual fear learning. For this, we examined the possibility that BA fear neurons may receive more vCA1 inputs that convey threat-predictive contextual information than other BA neurons do, which would facilitate their activation during contextual fear learning, thereby promoting synaptic potentiation and recruitment into a fear memory trace. To test this, we employed a retrograde trans-synaptic tracing approach with rabies virus (RV)^26^ in Arc-CreER^T2^ mice^8^, in which BA fear neurons were labeled more efficiently than in Fos-CreER^T2^ mice (Supplementary Fig. 17a–c). We first injected AAV-DIO-TVA-G-GFP into the BA (Fig. 8a). In the FC group, BA fear neurons were labeled with TVA-G-GFP, whereas BA neurons active in the home cages were labeled in the HC group (Fig. 8a, c). Fear learning-induced synaptic plasticity, which can affect RV-mediated labeling, was blocked with anisomycin in the FC group (Figs. 6f, 8a, b and Supplementary Fig. 18a). More BA neurons were labeled with TVA-G-GFP in the FC group than in the HC group (Supplementary Fig. 18b–d), indicating the recruitment of BA fear neurons by contextual fear conditioning. We then injected EnvA-expressing and G-deficient RV-mCherry into the BA, which infected TVA-G-expressing BA neurons (Fig. 8a, e) and propagated trans-synaptically, resulting in mCherry expression in vCA1 neurons monosynaptically projecting to TVA-G-GFP-labeled BA neurons (Fig. 8d). After 11 days, vCA1 neurons active in Context A were immunostained for c-Fos (Fig. 8a). The c-Fos+ proportion among all mCherry+ vCA1 neurons was significantly higher in the FC group than in the HC group, whereas there was no difference in the density of mCherry+ or c-Fos+ vCA1 neurons between groups (Fig. 8f, g). Thus, vCA1 neurons projecting to BA fear neurons were more likely to be c-Fos+ than vCA1 neurons projecting to BA neurons active in the home cages. As fear conditioning did not affect the excitability or intrinsic membrane properties of Context A vCA1 neurons (Supplementary Fig. 19), and anisomycin injection after fear learning prevented synaptic changes in the vCA1–BA pathway (Fig. 6f), these results suggest that more vCA1 neurons active in Context A likely project to BA fear neurons than to BA neurons active in the home cages.

We next used an electrophysiological approach to determine more clearly whether BA fear neurons receive more vCA1 inputs conveying threat-predictive contextual signals than other BA neurons do (Fig. 8h). vCA1 neurons active in Context A were labeled with ChR2, and BA neurons active during fear conditioning in Context A were labeled with tdT (Fig. 8i). In brain slices, monosynaptic AMPAR EPSCs were induced with photostimulation of ChR2-labeled vCA1 inputs and recorded in BA neurons. The peak amplitude of EPSCs recorded in tdT+ BA neurons was significantly larger than in adjacent tdT− BA neurons (Fig. 8k, l). As fear learning-induced synaptic strengthening of the vCA1–BA pathway was blocked by anisomycin injected after contextual fear conditioning (Figs. 6f, 8i, j), EPSC amplitude was proportional to the number of Context A vCA1 inputs to each BA neuron. Thus, these results suggest that BA fear neurons receive more vCA1 inputs that convey threat-predictive contextual information than other BA neurons do (Fig. 8m).

### Context-specific vCA1 activity contributes to fear learning

**Fig. 9:**
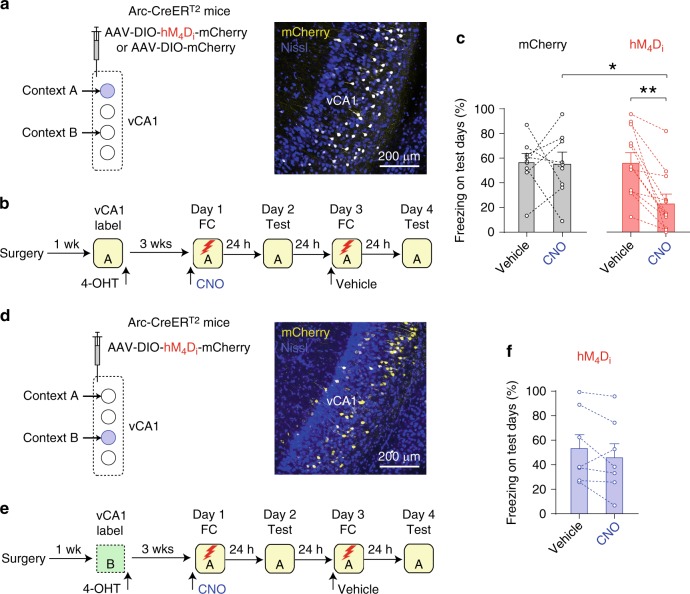
Context-specific vCA1 neuronal activity is involved in the acquisition of contextual fear memory. **a** Left: experimental setup for **b** and **c**. vCA1 neurons active in Context A expressed hM_4_D_i_-mCherry (hM_4_D_i_ group) or mCherry (mCherry group) in Arc-CreER^T2^ mice. Right: images showing mCherry-labeled vCA1 neurons (yellow). **b** Behavioral training and testing protocols for **c**. After labeling Context A vCA1 neurons, mice received a CNO or vehicle injection 30 min before fear conditioning in Context A and were tested for fear memory in Context A 24 h later. **c** Comparison of freezing behavior in Context A on the test days in the hM_4_D_i_ (12 mice; **p = 0.004, CNO vs. vehicle; *p = 0.016) and mCherry control groups (8 mice; *p* = 1.00, CNO vs. vehicle) (two-way ANOVA with post hoc comparisons; group × treatment interaction, *p* = 0.023). **d** Left: experimental setup for **e** and **f**. vCA1 neurons active in Context B were labeled with hM_4_D_i_-mCherry. Right: images showing mCherry-labeled vCA1 neurons (yellow). **e** After labeling Context B vCA1 neurons, mice received a CNO or vehicle injection 30 min before fear conditioning in Context A and were tested for fear memory in Context A 24 h later. **f** Comparison of freezing behavior in Context A on the test days (7 mice; *p* = 0.18, CNO vs. vehicle; two-sided paired *t*-test). Error bars represent the SEM. Source data are provided as a Source Data file. See also Supplementary Figs. 20–21.

To further determine the role of context-specific vCA1 neurons in contextual fear learning, we next examined how silencing vCA1 neurons active in a context affected the acquisition of fear memory for the context. To silence neural activity in a sufficient number of vCA1 neurons active in a context, we used Arc-CreER^T2^ mice, in which more vCA1 neurons were labeled than in Fos-CreER^T2^ mice (Supplementary Fig. 17d–g). We confirmed the context-specificity of vCA1 labeling in Arc-CreER^T2^ mice (Supplementary Fig. 20). vCA1 neurons active in Context A were labeled with hM_4_D_i_-mCherry or mCherry in Arc-CreER^T2^ mice (Fig. 9a, b). After 3 weeks, the mice received CNO or vehicle injections, underwent fear conditioning in Context A and were tested for freezing behavior in the same context 24 h later (Fig. 9b). When mice in the hM_4_D_i_ group were injected with CNO on the training day, they displayed less freezing behavior on the test day as compared with vehicle injection, whereas in the mCherry group, there was no difference in freezing behavior between CNO and vehicle injections (Fig. 9c). The CNO effect in the hM_4_D_i_ group on conditioned fear response was not due to the order of CNO and vehicle injections before fear conditioning (Supplementary Fig. 21). As silencing of Context A vCA1 neuronal activity during fear learning decreased conditioned fear responses to the context, activity in these vCA1 neurons contributes to the acquisition of contextual fear memory. When vCA1 neurons active in different Context B were labeled with hM_4_D_i_-mCherry, however, CNO injection before fear conditioning in Context A did not affect freezing behavior in Context A 24 h later, compared with the vehicle control (Fig. 9d–f), suggesting that silencing vCA1 neurons active in an irrelevant context did not affect contextual fear learning.

**Fig. 10:**
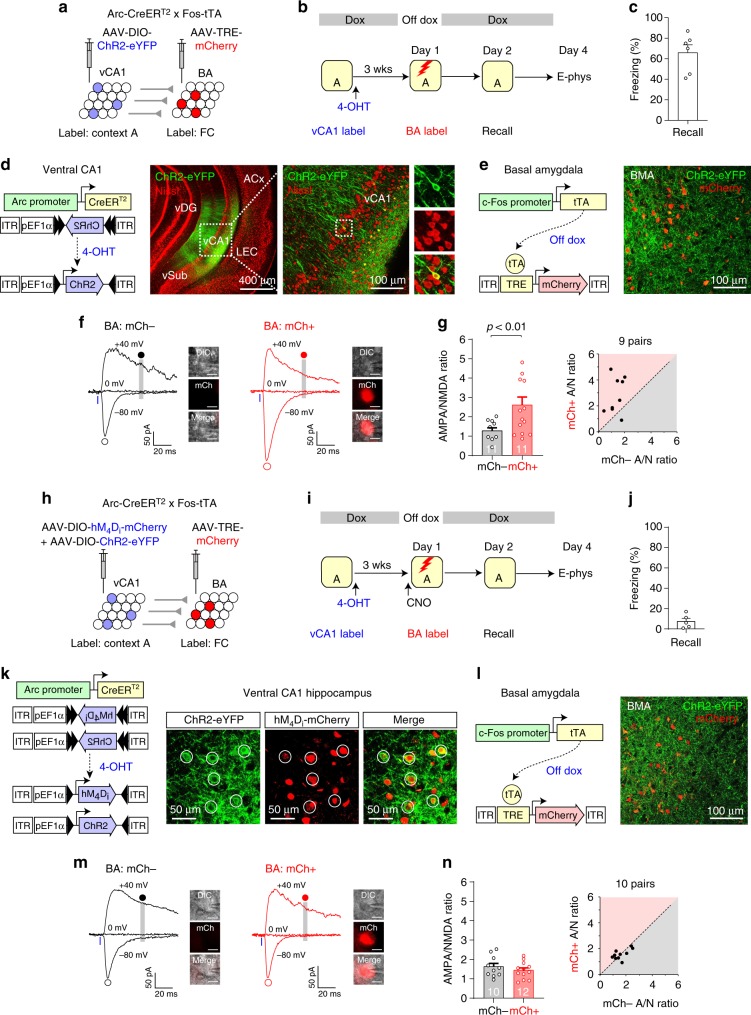
Silencing context-specific vCA1 neurons during fear conditioning inhibited both contextual fear learning and synaptic potentiation of the vCA1–BA pathway. **a** Experimental setup for **b**–**g**. AAV-DIO-ChR2-eYFP was injected into the vCA1, and AAV-TRE-mCherry was injected into the BA in Arc-CreER^T2^ × Fos-tTA mice. **b** Mice were fed with doxycycline (Dox)-containing food (gray horizontal bar). Mice were exposed to Context A and received a 4-OHT injection for ChR2 expression in vCA1 neurons active in Context A. Mice were then taken off Dox for 48 h and fear conditioned in Context A for mCherry expression in BA fear neurons. Mice were put back on Dox immediately after fear conditioning and tested for freezing behavior in Context A on Day 2. Recording experiments (E-phys) were performed on Day 4. **c** Quantification of freezing responses in Context A on Day 2. *n* = 6 mice. **d** Left: vCA1 neurons active in Context A express CreER^T2^ under the control of the Arc promoter, which then induced the recombination of the DIO in the presence of 4-OHT, resulting in permanent ChR2-eYFP expression. Right: images showing ChR2-eYFP-expressing vCA1 neurons (green). Red, Nissl stain. ACx, auditory cortex. **e** Left: BA neurons active during fear conditioning express tTA under the control of the c-Fos promoter, resulting in mCherry expression in the absence of Dox. Right: images showing mCherry+ BA neurons (red) and ChR2-eYFP-labeled vCA1 axons in the BA (green). **f** Traces of EPSCs recorded in mCherry (mCh)− and mCh+ BA neurons (inset; scale bar, 10 μm). EPSCs were induced by photostimulation of Context A vCA1 inputs and recorded in mCh− and mCh+ BA neurons. **g** Comparison of the AMPA/NMDA ratio between mCh− and mCh+ neurons (11 cells per group). ANOVA with post hoc comparisons was used to analyze combined data in **g** and **n**. **h** Experimental setup for **i**–**n**. Both AAV-DIO-hM_4_D_i_-mCherry and AAV-DIO-ChR2-eYFP were bilaterally injected into the vCA1, and AAV-TRE-mCherry was injected into the BA. **i** Mice were exposed to Context A and received a 4-OHT injection for the expression of both hM_4_D_i_ and ChR2 in vCA1 neurons active in Context A. Mice were then taken off Dox and fear conditioned in Context A for mCherry expression in BA fear neurons. The mice received a CNO injection 30 min before fear conditioning to inhibit the activity of Context A vCA1 neurons. They were put back on Dox immediately after fear conditioning and tested for freezing behavior on Day 2. Recording experiments were performed on Day 4. **j** Quantification of freezing responses in Context A on Day 2. **k** Left: vCA1 neurons active in Context A expressed CreER^T2^ under the control of the Arc promoter, which then induced the recombination of the DIO in the presence of 4-OHT, resulting in expression of both hM_4_D_i_-mCherry and ChR2-eYFP. Right: images showing vCA1 neurons (circles) expressing both hM4Di-mCherry (red) and ChR2-eYFP (green). **l** Left: BA neurons active during fear conditioning express tTA under the control of the c-Fos promoter, resulting in mCherry expression in the absence of Dox. Right: images showing mCherry+ BA neurons (red) and ChR2-eYFP-labeled vCA1 axons in the BA (green). **m** Traces of EPSCs induced by photostimulation of Context A vCA1 inputs and recorded in mCh− and mCh+ BA neurons (inset; scale bar, 10 μm) as in **f**. **n** Comparison of the AMPA/NMDA ratio between mCh− (10 cells) and mCh+ BA neurons (12 cells) (*p* = 1.00, two-way ANOVA with post hoc comparisons). Error bars represent the SEM. Source data are provided as a Source Data file. See also Supplementary Fig. 22.

We next examined how silencing of Context A vCA1 neurons during fear conditioning affected synaptic strength in the vCA1–BA pathway (Fig. 10). We first performed control experiments with Arc-CreER^T2^ × Fos-tTA mice, in which we independently induced ChR2-eYFP expression in vCA1 neurons active in Context A and mCherry expression in BA fear neurons active during fear conditioning (Fig. 10a–e). The mice showed freezing behavior in Context A 24 h after fear learning (Fig. 10c). In brain slices, photostimulation of Context A vCA1 inputs induced EPSCs that were recorded in BA neurons. The AMPA/NMDA ratio was significantly higher in BA fear neurons (mCherry+) than in adjacent BA neurons (mCherry−) (Fig. 10f, g), suggesting synaptic potentiation in Context A vCA1 inputs to BA fear neurons, consistent with our previous results in Fig. 7. We next induced the expression of both hM_4_D_i_-mCherry and ChR2-eYFP in vCA1 neurons active in Context A to silence these neurons in vivo and photostimulate their projections to the BA in brain slices (Fig. 10h–k). BA fear neurons active during fear conditioning in Context A were then labeled with mCherry while Context A vCA1 neuronal activity was silenced with CNO (Fig. 10l). Silencing Context A vCA1 neurons during fear conditioning inhibited freezing behavior in Context A 24 h later (Fig. 10j). In brain slices, photostimulation of Context A vCA1 inputs induced EPSCs that were recorded in BA neurons. There was no significant difference in the AMPA/NMDA ratio between mCherry+ and mCherry− BA neurons (Fig. 10m, n), indicating that silencing of Context A vCA1 neurons did not only inhibit contextual fear learning but it also blocked synaptic strengthening in the vCA1–BA pathway. Together, these results suggest the role of synaptic potentiation in the vCA1–BA pathway in encoding contextual fear memories (Supplementary Fig. 22).

## Discussion

In this study, we tested the hypothesis that contextual fear learning involves strengthening of functionally defined synapses, which connect context-encoding hippocampal CA1 neurons to a subset of neurons in the amygdala. The conventional approaches examining randomly selected synapses do not allow the efficient detection of associative learning-induced synaptic changes because associative memory is encoded sparsely in a subset of synapses^7,19,20,25,27^, and functional heterogeneity exists in brain areas orchestrating emotional learning and memory^28–30^. We recently developed an innovative approach by combining neural activity-dependent labeling^31^, optogenetic, and electrophysiological techniques, enabling efficient detection of synaptic changes in associative learning^7^. With this approach, we identified a BA neuronal population that receives more inputs from vCA1 neurons active in a context than other BA neurons do. Our study suggests that heterogeneous populations of BA neurons receive vCA1 inputs conveying different contextual information, while the total number of vCA1 inputs to each BA neuron is uniform. Although extensively used in previous studies^8,9,11,15^, neural activity-dependent labeling approaches have limitations including background labeling. As the labeling window (hours) is substantially longer than the labeling events (e.g., context exposure and contextual fear learning) (minutes), our labeling procedures likely tagged neurons active in the hours before and after the events, including those active in the home cages^31^. Thus, it is noteworthy that only a fraction of vCA1 and BA neurons labeled with our approaches represents neurons active during context exposure or contextual fear conditioning.

Contextual fear learning requires coordinated activity in the hippocampus and the amygdala^10,23,32,33^. Contextual representations encoded in the dorsal CA1 (dCA1) hippocampus are conveyed through the entorhinal and perirhinal cortices to the BA^33^, where they are integrated with aversive signals for fear memory formation^10^. Contextual information is also relayed to the BA through monosynaptic inputs from the vCA1^4,34^, which encodes a context with larger place fields and lower spatial selectivity than the dCA1^28,35^. Previous studies have implicated the vCA1 in contextual fear learning^3,6,36–38^. We found that silencing vCA1–BA activity during fear conditioning decreased conditioned fear responses 24 h later, indicating that vCA1–BA activity is involved in the acquisition of contextual fear memory, consistent with a previous report^14^. Moreover, optogenetic activation of vCA1 neurons projecting to the BA induced conditioned fear responses after it was paired with the US in a different context. Together, our results support the role of the vCA1–BA pathway in contextual fear learning.

As the vCA1 conveys contextual information directly to the amygdala, which generates conditioned fear responses^5^, contextual fear memory can be encoded by long-term potentiation (LTP) of vCA1–BA synapses, which subsequently facilitates the activation of the amygdala during fear memory recall in a threat-predictive context^6,39^. Our study demonstrates that synaptic potentiation was induced selectively in the vCA1 inputs conveying threat-predictive contextual information to the BA in discriminative contextual fear conditioning. Notably, LTP was not detected in vCA1 inputs conveying irrelevant contextual information to the BA or randomly selected vCA1–BA synapses in discriminative fear learning. Thus, the input specificity of LTP could confer the selectivity of contextual fear memory, enabling adaptive fear responses only to the relevant context, together with a well-documented role of the medial prefrontal cortex (mPFC)^40^. Therefore, the input specificity of synaptic potentiation may serve as the universal mechanism for selective fear responses to a context or sensory cue that predicts danger^7,19,20,25^.

We also found that input-specific LTP in contextual fear learning was confined to a subset of BA neurons active during contextual fear conditioning, which we termed BA fear neurons. vCA1 neurons active in a context are monosynaptically connected to BA fear neurons. Oure results suggest that LTP was selectively induced in context-specific vCA1 inputs to BA fear neurons in contextual fear conditioning, which is analogous to a previous report that contextual fear learning strengthens a subset of dorsal hippocampal CA3–CA1 synapses^19^. Thus, the locus of LTP for contextual fear memory is determined by both presynaptic vCA1 inputs and postsynaptic BA neurons, supporting the Hebbian model of associative learning and memory^41^. Moreover, strengthening of these vCA1–BA synapses was prevented by either post-training anisomycin or pretraining MK-801 injection. Notably, both anisomycin and MK-801 treatment also prevented conditioned fear responses to the context 24 h later, consistent with previous reports^23,24,42,43^. Therefore, impaired acquisition and consolidation of fear memory by MK-801 and anisomycin correlate with the lack of LTP in the vCA1–BA pathway, further supporting the role of LTP in the vCA1–BA pathway in contextual fear learning.

Which BA neuronal population is recruited into a contextual fear memory trace, and which neuronal or synaptic properties determine the allocation of contextual fear memory into a subset of BA neurons? A subset of BA neurons may receive more hippocampal inputs conveying threat-predictive contextual information and be recruited into a fear memory trace. Our trans-synaptic tracing and electrophysiological studies suggest that BA fear neurons active during fear conditioning receives more vCA1 inputs conveying threat-predictive contextual information than other BA neurons do. Although these results are correlational, our results raise the possibility that a BA neuronal population that receives more inputs from vCA1 neurons encoding relevant contextual representations could be more readily activated during contextual fear learning and, thus, would be preferentially recruited into a fear memory trace. Therefore, the number of vCA1 inputs to each BA neuron could contribute to determining which BA neurons are recruited into the contextual fear memory trace, together with a well-documented role of the transient changes in neuronal excitability in the selection of memory engram cells^21,22^. We did not detect increase in the membrane excitability in BA fear neurons 5 days after initial fear conditioning. This suggests that the neuronal excitability of BA fear neurons may be transiently increased during fear conditioning and then return to baseline once these cells have been recruited into a fear memory trace. This possibility is supported by previous reports that the excitability state of engram cells is dynamic, and a transient increase in neuronal excitability contributes to memory allocation and a more precise and effective retrieval of contextual fear memory^44,45^.

Previous studies suggest that functional heterogeneity exists in the vCA1. Depending upon their projection targets, vCA1 neuronal populations play distinct roles in various behavioral tasks^28^. vCA1 neurons projecting to the mPFC or CeA contribute to context-dependent expression of extinguished fear memory for an auditory cue^4,46^. Moreover, vCA1 neurons projecting to the nucleus accumbens play a role in social memory^47^, goal-directed behaviors^28^, and conditioned place preference for cocaine^20^. Previous studies also implicate granule cells in the ventral dentate gyrus and vCA1 neurons projecting to the lateral hypothalamus in innate anxiety and avoidance behavior^14,48^. In our study, silencing of context-specific vCA1 neurons during fear conditioning inhibited both the acquisition of contextual fear memory and synaptic strengthening of the vCA1–BA pathway, suggesting the role of the vCA1 in contextual fear learning. Our findings also implicate strengthening of the vCA1–BA pathway in the formation of contextual fear memory. Thus, our study provides important insights into the mechanism by which an associative fear memory for a particular context is encoded in the hippocampal–amygdala circuit.

## Methods

### Subjects

We obtained the heterozygous Fos-CreER^T2^ mice used in this study by crossing wild-type C57BL6/J (Jackson Laboratory Stock # 000664) and Fos-CreER^T2^ (+/−) mice (Jackson Laboratory Stock # 021882). We obtained heterozygous Arc-CreER^T2^ mice by crossing wild-type C57BL6/J and Arc-CreER^T2^ (+/−) mice (Jackson Laboratory Stock # 022357). We obtained heterozygous Fos-tTA/Fos-shGFP (+/−) mice by crossing wild-type C57BL6/J and Fos-tTA/Fos-shGFP (+/−) mice (Jackson Laboratory Stock # 018306). Fos-CreER^T2^ (+/−) and Ai9 ROSA-LSL-tdTomato (+/+) mice (Jackson Laboratory Stock # 007909) were crossed to generate Fos-CreER^T2^ (+/−) × ROSA-LSL-tdTomato (+/−) mice . Fos-CreER^T2^ (+/−) and Fos-tTA/Fos-shGFP (+/−) mice were crossed to generate Fos-CreER^T2^ (+/−) × Fos-tTA/Fos-shGFP (+/−) mice. Arc-CreER^T2^ (+/−) and Fos-tTA/Fos-shGFP (+/−) mice were crossed to generate Arc-CreER^T2^ (+/−) × Fos-tTA/Fos-shGFP (+/−) mice. GAD2-IRES-Cre (+/+) mice were obtained from the Jackson Laboratory (Stock # 010802). Mice were singly housed in home cages on a 12-h light/dark cycle with food and water continuously available. The light cycle was from 8 AM to 8 PM. Six- to eight-week-old mice of both sexes underwent stereotaxic brain surgery. All of the animal procedures were approved by the Institutional Animal Care and Use Committee of the University of California, Riverside.

### Virus constructs

The recombinant adeno-associated viruses (AAVs) were packaged by the Vector Core at the University of North Carolina. The AAV titers were 4.3 × 10^12^ genome copies (GC)/mL for AAV5-pCaMKIIα-eYFP, 6.5 × 10^12^ GC/mL for AAV5-pEF1α-DIO-eYFP, 5.3 × 10^12^ GC/mL for AAV5-pEF1α-DIO-mCherry, 4.0 × 10^12^ GC/mL for AAV5-pEF1α-DIO-hM_4_D_i_-mCherry, 5.5 × 10^12^ GC/mL for AAV5-pSyn-DIO-hM_4_D_i_-mCherry, 4.6 × 10^12^ GC/mL for AAV5-pCaMKIIα-hChR2(H134R)-eYFP, 4.2–7.0 × 10^12^ GC/mL for AAV5-pEF1α-DIO-hChR2(H134R)-eYFP, 5.7 × 10^12^ GC/mL for AAV5-pSyn-Chronos-GFP, 3.6 × 10^12^ GC/mL for AAV5-pEF1α-DIO-Chronos-GFP, and 3.9 × 10^12^ GC/mL for AAV1-pSyn-DIO-TVA-G-GFP. AAV9-TRE-hChR2(H134R)-eYFP and AAV9-TRE-mCherry constructs were obtained from Dr. Susumu Tonegawa at MIT and was packaged by Dr. Joung-Hun Kim’s laboratory at POSTECH (serotype 9, titer: 8 × 10^13^ and 8 × 10^12^ GC/mL for AAV-TRE-hChR2(H134R)-eYFP and AAV-TRE-mCherry, respectively). Herpes simplex virus (HSV-pEF1α-mCherry) for the retrograde tracing experiments was packaged by Dr. Rachael Neve at the Gene Delivery Technology Core of Massachusetts General Hospital, and the titer was >3.5 × 10^9^ infectious units/mL. Canine adenovirus (CAV2-pCMV-Cre) was obtained from Dr. Eric Kremer at the Montpellier Vector Platform of the Institute of Molecular Genetics of Montpellier, and the titer was 3.3 × 10^12^ viral particles/mL. Rabies virus (EnvA-ΔG-RV-mCherry) was obtained from Dr. John Naughton at the Gene Transfer, Targeting and Therapeutics Core of the Salk Institute for Biological Studies, and the titer was 1.4–2.3 × 10^8^ transducing units/mL.

### Surgery

Six- to eight-week-old mice underwent stereotaxic surgery. Prior to surgery, general anesthesia was induced by placing the mice in a transparent anesthetic chamber filled with 5% isoflurane with intramuscular injection of ketamine and xylazine (30 mg/kg and 2 mg/kg body weight, respectively). The anesthesia was maintained during surgery with 1% isoflurane applied to the nostrils of the mice using a precision vaporizer. Mice were checked for the absence of the tail-pinch reflex as a sign of sufficient anesthesia. The mice were then immobilized in a stereotaxic frame with non-rupture ear bars (David Kopf Instruments), and ophthalmic ointment was applied to prevent eye drying. After an incision was made along the midline of the scalp, small unilateral or bilateral craniotomies were performed using a microdrill with 0.5-mm burrs. The tips of glass capillaries loaded with AAV were placed into the vCA1 (3.4 mm caudal to bregma, 3.7 mm lateral to the midline, and 3.2 mm ventral to the pial surface) or BA (1.5 mm caudal to bregma, 3.2 mm lateral to the midline, and 3.5 mm ventral to the pial surface). AAV-containing solution was injected at a rate of 0.1 μL/minute using a 10 μL Hamilton microsyringe and a syringe pump. The total volume of injected virus-containing solution was 0.15 μL for AAV5-pCaMKIIα-eYFP, 0.5 μL for AAV5-pEF1α-DIO-eYFP, 1.0 μL for AAV5-pEF1α-DIO-mCherry, 1.0 μL for AAV5-pEF1α-DIO-hM_4_D_i_-mCherry, 1.0 μL for AAV5-pSyn-DIO-hM_4_D_i_-mCherry, 0.15 μL for AAV5-pCaMKIIα-ChR2(H134R)-eYFP, 1.0 μL for AAV5-pEF1α-DIO-ChR2(H134R)-eYFP, 0.5 μL for AAV5-pSyn-Chronos-GFP, 0.5 μL for AAV5-pEF1α-DIO-Chronos-GFP, 0.5 μL for AAV1-pSyn-DIO-TVA-G-GFP, 0.5 μL for AAV9-TRE-hChR2(H134R)-eYFP, 0.5 μL for AAV9-TRE-mCherry 1.0 μL for HSV-pEF1α-mCherry, 1.0 μL for CAV2-pCMV-Cre, and 0.5-0.8 μL for EnvA-ΔG-RV-mCherry. In Fig. 10h–n, a mixture of 0.5 μL AAV5-pEF1α-DIO-ChR2(H134R)-eYFP and 0.5 μL AAV5-pSyn-DIO-hM_4_D_i_-mCherry was bilaterally injected to the vCA1. After injection, the capillary was left in place for an additional 5 min to allow diffusion of the virus solution and then withdrawn. The scalp incision was closed with surgical sutures, and the mice were given buprenorphine-containing saline (1 mL, 0.13 mg buprenorphine/kg body weight) for postoperative analgesia and hydration.

For the experiments described in Fig. 2, an optical cannula (200 μm in diameter, numerical aperture of 0.53, Doric Lenses) was implanted above the left vCA1 (3.4 mm caudal to bregma, 3.8 mm lateral to the midline, and 2.15 mm ventral to the pial surface) and secured with dental cement. To minimize light leakage during photostimulation, which can act as a visual cue, we painted all optical pathways, including the dental cement securing the cannula, with black nail polish. We verified the cannula implantation site in each animal (Fig. 2b).

### Activity-dependent neuronal labeling

For functional labeling of vCA1 and BA neurons, we used Fos-CreER^T2^, Arc-CreER^T2^, and Fos-tTA/Fos-shGFP mice or Fos-tTA mice for short. To open labeling window, we intraperitoneally injected tamoxifen (T5648, Sigma-Aldrich) into Fos-CreER^T2^ mice and 4-hydroxytamoxifen (4-OHT; H6278, Sigma-Aldrich) into Arc-CreER^T2^ mice. Tamoxifen was dissolved in corn oil (C8267, Sigma-Aldrich) at 20 mg/mL with nutation for 6 h in the dark at room temperature (22–24 °C). 4-OHT was dissolved in DMSO (40 mg/mL) and further dissolved in saline containing 2% TWEEN 80 at 2 mg/mL in a water bath at 37 °C. The 4-OHT solution was then diluted with an equal volume of saline, resulting in 1 mg/mL 4-OHT solution. Fos-tTA mice were continuously fed with doxycycline (Dox)-containing food (200 mg doxycycline/kg food pellet; Bio-Serv, Cat# S3888) starting from 7 days before virus injection surgery. The mice were taken off Dox for 48 h to open labeling window and put back on Dox immediately after labeling event.

Functional labeling of vCA1 neurons active in a context: For labeling of vCA1 neurons active in a context in Fos-CreER^T2^ mice in Figs. 3, 4, 5a–g, 6a–g and Supplementary Figs. 4, 5, 6, 7a–b, 9, 10, 11, 12, 13, 15, 17d, 19, mice received an intraperitoneal injection of tamoxifen (150 mg/kg of body weight, 0.2–0.3 mL) 1 week after surgery. The mice were exposed to novel Context A (dimension: 30 cm × 24 cm × 21 cm; stainless steel grid floor, white light illumination, and benzaldehyde odor) or Context B (dimension: 30 cm × 24 cm × 21 cm; acrylic plate floor, dim red light illumination, and acetic acid odor) within a standard fear conditioning chamber (Med Associates) three times for 12 min each at 14, 19, and 24 h after tamoxifen injection. In some experiments, the mice received 1 or 2 additional labeling procedures with a 1-week interval. To minimize neuronal labeling by background noise, mice were kept in the dark in their home cage in a quiet place with minimal traffic within a satellite animal care facility for 36 h after tamoxifen injection. Our context labeling procedure induced the most abundant transgene expression in the vCA1, the virus injection site, compared with other subregions of the hippocampus (ChR2-eYFP-labeled CA1 neurons/mm^3^: 0.02 ± 0.01, 0.34 ± 0.11, 0.97 ± 0.17, and 0.66 ± 0.18 for the dorsal CA1, intermediate CA1, ventral CA1, and ventral subiculum, respectively; average ± SEM; 10 mice).

To label vCA1 neurons active in a context in Arc-CreER^T2^ mice in Figs. 9, 10 and Supplementary Figs. 17e, 20, 21, mice were exposed to Context A or Context B once for 12 min and returned to their home cages. After 10–20 min, the mice received an intraperitoneal injection of 4-hydroxytamoxifen (4-OHT, 15–30 mg/kg of body weight). To minimize neuronal labeling by background noise, mice were kept in the dark in their home cages in a quiet place for 12 h before and after context exposure. To avoid the confounding effect of the 4-OHT injection on fear memory formation, we injected 4-OHT 10–20 min after context exposure or fear conditioning, which induces neuronal labeling with a sensitivity and specificity comparable to that observed when 4-OHT is administered before the labeling event^49^.

To label context-specific vCA1 neurons in Fos-tTA mice in Fig. 7 and Supplementary Fig. 16, the mice were taken off Dox and exposed to novel Context A within a fear conditioning chamber three times for 12 min each at 48, 53, and 58 h after the start of Off-Dox. Immediately after the last context exposure, the mice were then put back on Dox. To minimize neuronal labeling by background noise, mice were kept in the dark in their home cage in a quiet place from 2 h before the first context exposure to 2 h after the last context exposure. In Fig. 7, the mice received two additional labeling procedures with a 1-week interval.

Labeling BA fear neurons for electrophysiological recording: To label BA fear neurons, which refer to BA neurons active during fear conditioning, in Fos-CreER^T2^ mice in Figs. 5, 7 and Supplementary Figs. 12, 15, we injected the mice intraperitoneally with tamoxifen (150 mg/kg of body weight). After 24 h, the mice were placed in Context A within the fear conditioning chamber and given 3 or 5 unconditioned stimuli (US, 0.5 mA electric footshocks for 2 s) at 120 s intervals. To minimize neuronal labeling by background noise, mice were kept in their home cage in a quiet place for 36 h after tamoxifen injection.

To label BA fear neurons in Fig. 6a–g, we injected the mice intraperitoneally with tamoxifen (150 mg/kg of body weight). After 14 h, the mice were fear conditioned in Context A as in Supplementary Fig. 14a. Immediately after fear conditioning, the mice received an intraperitoneal injection of anisomycin (150 mg/kg of body weight) to inhibit the consolidation of contextual fear memory in the experimental group (ANI group) or were injected with the same volume of saline (SAL group). Mice received 3 more injections of anisomycin (50 mg/kg body weight) or saline at 2-hour intervals. Anisomycin solution (10 mg/mL, Sigma-Aldrich) was prepared freshly each day by dissolving 20 mg of anisomycin in 2 mL of saline containing 75 μL of 1 M HCl and adjusting the pH to approximately 7.0 with 1 M NaOH.

To label BA fear neurons in Arc-CreER^T2^ × Fos-tTA mice in Fig. 10, the mice were taken off Dox for 48 h and fear-conditioned in Context A. Mice were put back on Dox immediately after fear conditioning and fed with Dox.

Labeling of BA fear neurons for trans-synaptic tracing study: To label BA fear neurons in the FC group in Arc-CreER^T2^ mice in Fig. 8a–g, the mice were placed in Context A within the fear conditioning chamber and given 3 US (0.5 mA electric footshocks for 2 s) at 60 s intervals as in Supplementary Fig. 18a. Immediately after fear conditioning, the mice were injected intraperitoneally with anisomycin (150 mg/kg of body weight) to inhibit protein synthesis-dependent synaptic changes induced by fear conditioning. Ten minutes after contextual fear conditioning, the mice also received an intraperitoneal injection of 4-OHT (15 mg/kg of body weight). Mice received 3 more injections of anisomycin (50 mg/kg body weight) or saline at 2-hour intervals. To label BA neurons active in the home cage, mice in the home cage (HC) group were kept in their home cages and were given an anisomycin injection as in the FC group. Ten minutes later, the mice also received a 4-OHT injection and 3 more injections of anisomycin (50 mg/kg body weight) at 2-hour intervals as in the FC group. To minimize neuronal labeling by background noise, mice were kept in the dark in their home cages in a quiet place for 12 h before and after 4-OHT injection.

### Single-trial contextual fear conditioning

In the experiments described in Figs. 1, 6h–j, 9, 10 and Supplementary Figs. 17a–c, 19, mice were singly housed in their home cages on a 12-h light/dark cycle starting a week before behavioral training, with food and water continuously available. Mice were randomly assigned to behavioral groups. On the training day, mice were placed in Context A between 9 AM and 10 AM. After 3 min, the mice received the first US (electric footshock, 0.5 mA, 2 s duration) and were given 4 more US with a 2-minute interval as in Supplementary Fig. 1a. The temperature in the fear conditioning chamber was 22–24 °C. On the test day, freezing behavior was quantified as the percentage of time immobile in the first 5 min in Context A. The movement of the mice in the fear conditioning chamber was recorded using a near-infrared camera and analyzed in real-time with EthoVision XT 11 software (Noldus). Freezing score was calculated as the percentage of time for which the mice remained immobile. Immobility for more than 2 s was counted as freezing behavior.

In Figs. 6a–g, 8a–g and Supplementary Figs. 14, 18b–d, mice were placed in Context A on the training day. After 3 min, the mice received the first US (electric footshock, 0.5 mA, 2 s duration) and were given 2 more US with a 1-min interval. After fear conditioning, the mice received intraperitoneal injections of anisomycin or saline. In Fig. 8a–g and Supplementary Fig. 18b–d, mice in the HC group remained in their home cages on the training day. In Supplementary Fig. 15, mice in the MK-801 group received an intraperitoneal injection of MK-801 (0.3 mg/kg body weight) or saline 30 min before fear conditioning with three US in Context A.

### Discriminative contextual fear conditioning

In the experiments described in Fig. 4 and Supplementary Figs. 9, 10, 11, mice were singly housed in their home cages on a 12-h light/dark cycle starting a week before behavioral training, with food and water continuously available. Mice were randomly assigned to either the fear conditioning (FC) group or the no shock (NS) control group. On the training days (Days 1–5), mice in the FC group were exposed to Context B for 5 min between 9 AM and 10 AM (see Supplementary Fig. 1b). After 1 h, the mice were placed in Context A and given a US (electric footshock, 0.5 mA, 2 s duration) 4 min later. On the same days, the mice received a US in Context A between 6 PM and 7 PM and then were exposed to Context B without the US an hour later. In each training session, mice were tested for freezing behavior for the first 4 min in Contexts A or B. On Day 6, brain slices were prepared from these mice for electrophysiological recording experiments. Mice in the NS control group were similarly exposed to Contexts A and B on Days 1–5 but never received the US. Freezing behavior in Contexts A and B was quantified as % of time immobile in the first 4 min in Context A and B. Freezing score was calculated as the percentage of time for which the mice remained immobile. Immobility for more than 2 s was counted as freezing behavior. The freezing scores for Context A and B were averaged for each day. Mice with generalized contextual fear responses in the FC group (i.e., freezing score in Context B > 35%) were excluded from analysis in Fig. 4g–k, in which we examined how the input specificity of synaptic strengthening in context-specific vCA1–BA pathway contributes to the selectivity of conditioned fear responses to a context that predicts danger (i.e., Context A).

For discriminative contextual fear conditioning in Fig. 5 and Supplementary Fig. 12, mice were injected with tamoxifen and given 5 US in Context A 24 h later (Day 1). On Days 2–5, mice were trained for discriminative fear learning as described above for the FC group. On Day 6, brain slices were prepared for electrophysiological recording experiments.

### In vivo chemogenetic silencing of vCA1 activity

For experiments in Fig. 1e–h, retrograde CAV2-pCMV-Cre was bilaterally injected into the basal amygdala (BA), and AAV-pEF1α-DIO-hM_4_D_i_-mCherry (hM_4_D_i_ group) or AAV-pEF1α-DIO-mCherry (mCherry group) was bilaterally injected into the vCA1. Four weeks after surgery, mice received an intraperitoneal injection of clozapine N-oxide (CNO, 10 mg/kg body weight) on Day 1 and were given 5 shocks in Context A 30 min later as in Supplementary Fig. 1a. CNO was dissolved in DMSO (10 mg/mL) and then further dissolved in saline at 1 mg/mL. After 24 h, the mice were tested for freezing behavior in Context A on Day 2. On Day 3, the mice received an intraperitoneal injection of vehicle (the same volume of saline containing 10% DMSO as in the CNO injection on Day 1) and were given 5 shocks in Context A 30 min later as in Supplementary Fig. 1a. After 24 h, the mice were tested for freezing behavior in Context A on Day 4. In Supplementary Fig. 2d–e, mice received a vehicle injection 30 min before fear conditioning in Context A on both Day 1 and Day 3.

For the experiments presented in Figs. 9 and 10h–n, AAV-pEF1α-DIO-hM_4_D_i_-mCherry (hM_4_D_i_ group) or AAV-pEF1α-DIO-mCherry (mCherry group) was bilaterally injected into the vCA1 in Arc-CreER^T2^ mice. A week after surgery, the mice were exposed to Context A or Context B for 12 min and received an intraperitoneal injection of 4-OHT (15–30 mg/kg of body weight) to label vCA1 neurons active in Context A or Context B. Three weeks after behavioral labeling, mice received an intraperitoneal injection of CNO (Day 1) or vehicle (Day 3) and fear conditioned in Context A 30 min later as in Supplementary Fig. 1a. A day after contextual fear conditioning, the mice were tested for freezing behavior in Context A on Days 2 and 4. In Supplementary Fig. 21, after surgery and labeling of vCA1 neurons in Context A, mice received a vehicle injection 30 min before fear conditioning in Context A on both Day 1 and Day 3.

### In vivo optogenetic stimulation for fear memory formation

To activate vCA1 neurons projecting to the BA (vCA1: BA projectors) in Fig. 2a–e, we injected retrograde CAV2-pCMV-Cre into the BA and AAV-DIO-Chronos-GFP (Chronos group) or AAV-DIO-eYFP (eYFP group) into the vCA1. An optical cannula was implanted dorsal to the vCA1 to illuminate vCA1: BA projectors expressing Chronos-GFP or eYFP. Three weeks after surgery, mice were habituated to connection of the optical cable and photostimulation of the vCA1 in Context C (dimension: 30 cm × 24 cm × 21 cm; acrylic plate floor, no illumination, and acetic acid odor) for 3 days (Days 1–3). After a 3-minute acclimatization period and baseline recording of freezing behavior for 1 min, 20 Hz pulses of blue light illumination (450 nm laser, 22–25 mW/mm^2^, 1 ms pulses) were applied to the vCA1 through an optical cannula for 1 min. On Day 4, the mice received 6 pairings of the same 20 Hz photostimulation (20 s duration) and an electric footshock (0.5 mA, 2 s duration, co-terminating with photostimulation) in Context A. On Days 5–6, freezing behavior was monitored in Context C in the presence and absence of the 20 Hz photostimulations (1-minute duration). Each mouse underwent the freezing test once per day for 2 days. Freezing scores in the presence or absence of blue light illumination during habituation and test sessions were calculated separately on each test day and averaged. For each mouse, we then calculated the difference in the average freezing scores in the presence and absence of photostimulation (ON – OFF freezing) during habituation (Days 1–3) and testing (Days 5–6). In the Chronos: unpaired group in Fig. 2f, g, we injected CAV2-pCMV-Cre into the BA and AAV-DIO-Chronos-GFP into the vCA1 and implanted an optical cannula dorsal to the vCA1 as in the Chronos: paired group. After surgery, the mice were habituated in Context C as in the Chronos: paired and eYFP group on Days 1–3. On training Day 4, the mice received 20 Hz photostimulation (20 s duration) six times at 120 s intervals in Context A and were returned to their home cages. After 30 min, the mice were placed in Context A again and received 6 shocks (0.5 mA, 2 s duration, 120 s intervals) without photostimulation. On Days 5–6, the mice were tested for freezing behavior in Context C in the presence and absence of the 20 Hz photostimulation.

### Trans-synaptic viral tracing

We first injected AAV-DIO-TVA-G-GFP into the BA in Arc-CreER^T2^ mice in Fig. 8a–g. A week after surgery, mice in the fear conditioning (FC) group were placed in Context A within the fear conditioning chamber and given 3 US (0.5 mA electric footshocks for 2 s each) at 60 s intervals. Immediately after fear conditioning, the mice were injected intraperitoneally with anisomycin (150 mg/kg of body weight) to inhibit protein synthesis-dependent synaptic changes induced by fear conditioning (see Supplementary Fig. 18a). Ten minutes after contextual fear conditioning, the mice also received an intraperitoneal injection of 4-OHT (15 mg/kg of body weight). Mice in the home cage (HC) control group remained in their home cages until they received anisomycin and 4-OHT as the FC group did. Mice in both groups received three additional anisomycin injections (50 mg/kg of body weight each) 2, 4, and 6 h later. Two weeks later, we injected the BA with EnvA-expressing and G-deficient RV-mCherry (EnvA-ΔG-RV-mCherry), which infected TVA/G-labeled BA neurons and propagated trans-synaptically, resulting in mCherry expression in neurons monosynaptically projecting to labeled BA neurons. After 11 days, the mice in both groups were exposed to Context A, and brain tissue was fixed 90 min later such that vCA1 neurons active in Context A were labeled by c-Fos immunostaining (see below).

### Histology, microscopic imaging, and cell counting

Acute brain slices (300 μm thick) were prepared with a vibratome (VT-1000S, Leica Biosystems) and fixed in 4% paraformaldehyde in phosphate buffered saline (PBS, 137 mM NaCl, 2.7 mM KCl, 11.9 mM phosphate, pH 7.4) at 4 °C overnight. After fixation, slices were washed twice in PBS containing 0.3% Triton X-100 (PBS-T) at room temperature for 10 min and permeabilized in PBS-T at 4 °C overnight. For Nissl staining, slices were incubated with Neurotrace fluorescent Nissl stain (1:40 diluted in PBS, Thermo Fisher Scientific) for 3 h at room temperature and washed in PBS-T three times for 10 min each. After a final wash in PBS-T, Vectashield mounting medium (Vector Laboratories) was applied to the slices, which were then covered with coverslips. Microscopic images were captured using the Leica TCS SP5 confocal system (Leica Microsystems). Images captured with different fluorescent channels were merged using ImageJ software (National Institute of Mental Health). For each mouse, the virus injection site was verified by the expression of fluorescent markers in the vCA1 and BA. Mice in which the target area was missed were excluded from the analysis.

To quantify the proportion of labeled vCA1 neurons (eYFP+) among all vCA1: BA projectors in Fig. 3a–c, we captured confocal microscopic images of 3-4 representative fields (0.56 mm^2^ each) per mouse within the vCA1, where mCherry+ neurons were distributed most densely. The confocal images were then Z-stacked using ImageJ software. vCA1: BA projectors (mCherry+) and functionally labeled neurons (eYFP+) were identified based on the fluorescence labeling of cell bodies. The proportions of eYFP-labeled vCA1 among all vCA1: BA projectors (mCherry+) were calculated in each field of the vCA1 and averaged for each mouse.

The proportion of ChR2-labeled vCA1 neurons among all vCA1 cells in Fig. 3i, was calculated by dividing the number of ChR2-eYFP+ cells by the total number of DAPI+ cells within the pyramidal layer of the vCA1. ChR2-eYFP + vCA1 neurons were counted manually, and DAPI+ cells in the vCA1 were counted using the spot detection function of Imaris 9 software (Bitplane).

For fluorescent labeling of the recorded neurons in Figs. 3j, 6d, neurons were loaded with the pipette solution (see below) containing 5 mM biocytin for 20 min. The pipette was then withdrawn slowly, and the brain slices were fixed at 4 °C overnight with 4 % paraformaldehyde. After fixation, slices were washed with PBS-T twice for 10 min each and incubated with streptavidin-Alexa Fluor 633 conjugate (20 μg/mL in PBS, Thermo Fisher Scientific) for 2 h at room temperature. The unbound streptavidin was then washed out with PBS three times for 20 min each, and the slices were mounted onto slides. Images of the labeled neurons were taken using a Leica TSC SP5 confocal microscope, and neuronal morphology and location within the BA were analyzed.

To examine what proportion of BA and vCA1 neurons active during contextual fear conditioning were reactivated during recall of the fear memory in Fig. 6h–j, we identified BA and vCA1 neurons active during contextual fear conditioning or in the home cages with mCherry expression. BA and vCA1 neurons active during fear memory recall were immunostained for c-Fos (see below). We captured confocal microscopic images of 3–4 representative fields (0.56 mm^2^ each) of the BA and vCA1, where mCherry-labeled neurons were most abundant. The proportions of c-Fos+ neurons among all mCherry+ BA or vCA1 neurons within the field of view were calculated and averaged for each mouse.

To examine how many vCA1 neurons projecting to behaviorally labeled BA neurons are specific to Context A in Fig. 8g, we calculated the c-Fos+ proportion among all RV-mCherry-labeled vCA1 neurons. We captured confocal microscopic images of 4 representative fields (0.56 mm^2^ each) in the vCA1, where mCherry-labeled neurons were most abundant within the vCA1. The proportion of c-Fos+ neurons among all mCherry+ neurons within the field of view was calculated and averaged for each mouse. mCherry+ or c-Fos + vCA1 neurons were counted manually. The probability of a randomly selected vCA1 neuron being c-Fos+ was calculated by dividing the number of c-Fos+ cells by the total number of DAPI+ cells within the pyramidal layer of the vCA1. Fos+ and DAPI+ cells in the vCA1 were counted using the spot detection function of Imaris 9 software (Bitplane).

### c-Fos immunohistochemistry and analysis

Brain slices of the vCA1 or the BA (100 μm thick) were prepared with a vibratome and fixed 90 min after the last behavioral test in Figs. 1c, d, 3d–f, 6h–j, 8a–g and Supplementary Figs 5c–e, 14b–d, 16. After fixation in 4% paraformaldehyde in PBS for an hour, the slices were permeabilized in PBS-T at room temperature for 4 days. Brain sections were then blocked with PBS containing 5% goat serum at 4 °C for an hour. The slices were washed with PBS-T for 10 min and incubated with a polyclonal affinity purified rabbit anti-c-Fos antibody (1:200 dilution of 0.1 mg/mL stock antibody in PBS-T, 226033/Synaptic Systems) at 4 °C overnight. The slices were then washed with PBS-T three times for 10 min each and incubated with goat anti-rabbit IgG antibody-Alexa Fluor 647 (1:200 in PBS-T) at 4 °C overnight. The slices were then washed three times with PBS-T for 10 min each and mounted on glass slides for confocal microscopic imaging.

For each mouse, we captured Z-series confocal microscopic images of 4 representative fields (0.56 mm^2^ each) of the vCA1 or the BA and Z-stacked the images using ImageJ software. We identified vCA1: BA projectors (labeled with mCherry) in Fig. 1c, d, vCA1 neurons active in Context A (labeled with tdTomato or mCherry) in Fig. 3d–f and Supplementary Figs 5c–e, vCA1 and BA neurons active during fear conditioning or active in the home cage (labeled with mCherry) in Fig. 6h–j, and vCA1 neurons trans-synaptically labeled with mCherry in Fig. 8a–g. We manually counted these labeled neurons and c-Fos+ neurons in the BA and vCA1 except Fig. 8f, g, in which c-Fos+ cells in the vCA1 were counted using Imaris 9 software. We then calculated the proportion of c-Fos+ neurons among all labeled neurons in each vCA1 or BA field and averaged the proportions for each mouse. In Supplementary Fig. 14b–d, we manually counted c-Fos+ cells in Z-stacked confocal microscopic images of 4 representative fields (0.56 mm^2^ each) of the vCA1, BMA or BA and calculated the density of c-Fos+ cells by dividing total number of c-Fos+ cells with the volume of interest.

### Verification of the specificity of neuronal labeling

To examine the specificity of behavioral labeling of the vCA1 in Fos-CreER^T2^ x ROSA-LSL-tdTomato mice in Fig. 3d–f, we labeled vCA1 neurons at two different time points such that tdTomato+ cells reflected neurons labeled during the first Context A exposure, whereas c-Fos+ cells reflected neurons labeled during the second context exposure to either Context A or B one week later. We captured confocal microscopic images of 3-4 representative fields (0.56 mm^2^ each) in the vCA1, where tdTomato+ neurons were most abundant within the vCA1. tdTomato+ or c-Fos+ vCA1 neurons were counted manually. The proportion of c-Fos+ neurons among all tdTomato+ neurons within the field of view was calculated and averaged for each mouse.

To examine the context-specificity of labeling of the vCA1 in Fos-tTA mice in Supplementary Fig. 16, we labeled vCA1 neurons at two different time points such that ChR2-eYFP+ cells reflected vCA1 neurons active during the first Context A exposure, whereas c-Fos+ cells reflected vCA1 neurons active during the second Context A exposure in the A–A group or vCA1 neurons active in the home cages in the A–HC group 9 days after the first context exposure. We captured confocal microscopic images of 3–4 representative fields (0.56 mm^2^ each) in the vCA1, where ChR2-eYFP+ neurons were most abundant within the vCA1. The proportion of c-Fos+ cells among all ChR2-eYFP + vCA1 neurons within the field of view was calculated and averaged for each mouse.

To examine the context-specificity of behavioral labeling of the vCA1 in Arc-CreER^T2^ × Fos-tTA/Fos-shGFP mice in Supplementary Fig. 20, we labeled vCA1 neurons at two different time points such that mCherry+ cells indicated neurons labeled during the first Context A or B exposure, whereas short half-life (2 h) GFP+ (shGFP+) cells indicated neurons labeled during the second Context A exposure 7–11 days later. We captured confocal microscopic images of 3–4 representative fields (0.56 mm^2^ each) of the vCA1, where mCherry+ neurons were most abundant. The proportion of shGFP+ neurons among all mCherry+ neurons within the field of view was calculated and averaged for each mouse.

### Whole-cell patch-clamp recording in brain slices

For electrophysiological recording in brain slices, mice were deeply anesthetized with 5 % isoflurane and decapitated. Brains were dissected quickly and chilled in ice-cold artificial cerebrospinal fluid (ACSF) containing 130 mM NaCl, 2.5 mM KCl, 2.5 mM CaCl_2_, 1 mM MgSO_4_, 1.25 mM NaH_2_PO_4_, 26 mM NaHCO_3_, and 10 mM glucose with 95% O_2_ and 5% CO_2_. Coronal brain slices containing the amygdala (300 μm thick) were prepared with the vibratome. After a 1-hour recovery period, slices were placed in the recording chamber and continuously perfused with ACSF at a rate of 1 mL per minute. The patch electrodes (2–3 MΩ resistance) were filled with pipette solution containing 140 mM Cs-methanesulfonate, 5 mM NaCl, 1 mM MgCl_2_, 10 mM HEPES, 0.2 EGTA, 2 mM MgATP, 0.5 mM NaGTP, and 5 mM QX-314 chloride (290 mOsm, adjusted to pH 7.3 with CsOH). For experiments in Fig. 5h–j and Supplementary Figs 2a–c, 3, 19, we replaced 140 mM Cs-methanesulfonate with 150 mM K-gluconate without QX-314 added. We recorded EPSCs in putative principal neurons in the BA with a membrane capacitance larger than 100 pF (see Supplementary Fig. 8). For morphological analysis of recorded neurons in Figs. 3j, 6d, we added biocytin (5 mM) to the pipette solution. Whole-cell patch clamp recordings were performed at 30–32 °C using a Multiclamp 700B amplifier, a Digidata 1550 A or 1320 A digitizer and Clampex 10 software (Molecular Devices). As the temperature affects the kinetics of EPSCs, the temperature of recording chamber was carefully monitored to be 30–32 °C. The membrane potential was held constant at −80 mV in voltage-clamp mode unless otherwise indicated. The liquid junction potentials of 8.9 mV and 15.5 mV were corrected for the Cs-based pipette solution and for the K-gluconate-based pipette solution, respectively. Series (access) resistance was not compensated. Offline data analysis was performed using the Clampfit 10 (Molecular Devices). Results from electrophysiological experiments are summarized in Supplementary Tables 3 and 4.

Photostimulation in brain slices: A blue collimated light-emitting diode (LED) with a peak wavelength of 470 nm (M470L3, Thorlabs) was used for photostimulation of ChR2- or Chronos-expressing axons. The LED was connected to the amplifier and digitizer through an LED driver (LEDD1B, Thorlabs). Brain slices in the recording chamber were illuminated through a 40× water-immersion objective lens (Olympus LUMPLFLN 40XW or Leica HCX APO L40x #506155) and a 450–490 nm filter (Chroma). The illumination area was 0.17 mm^2^ and was centered at the soma of the neuron patched for recording. The intensity and duration of photostimulation were controlled using a Digidata 1550 A or 1320 A digitizer and Clampex 10 software (Molecular Devices). Light power in milliwatts (mW) was measured at 470 nm using a power meter (PM100A, Thorlabs) placed under the objective lens, and light power density (mW/mm^2^) was calculated by dividing light power by illumination area. To evoke synaptic responses in the BA by photostimulation of vCA1 axons, we illuminated the slices every 20 s with blue light pulses of 1 ms duration (2.8–20 mW/mm^2^). When apparent polysynaptic activity was detected in EPSC recordings, we reduced the photostimulation intensity to prevent polysynaptic components from contributing to our measurement of AMPAR and NMDAR EPSC amplitudes. When we could not eliminate polysynaptic activity by adjusting the stimulation intensity, we terminated the experiments for the recorded neurons.

AMPA/NMDA EPSC ratio: AMPAR EPSCs were recorded at −80 mV, and NMDAR EPSCs were recorded at +40 mV in voltage-clamp mode. SR-95531 (10 μM), a GABA-A receptor antagonist, was added to the ACSF to prevent contamination from inhibitory postsynaptic currents in the feed-forward inhibitory circuit. Photostimulation intensity was adjusted such that the peak amplitude of AMPAR EPSCs was 50–250 pA. For each BA neuron, the same photostimulation intensity and duration were used to record AMPAR and NMDAR EPSCs. To calculate the AMPA/NMDA EPSC ratio, we recorded the first set of AMPAR EPSCs (3–5 traces) at −80 mV and then recorded NMDAR EPSCs (3–5 traces) at +40 mV. Then, the holding potential was returned to −80 mV to record the second set of AMPAR EPSCs (3–5 traces). We also recorded EPSCs at 0 mV. This recording protocol minimized the effect of time-dependent EPSC changes on the AMPA/NMDA ratio. To quantify AMPAR EPSCs, we averaged the first and second sets of AMPAR EPSCs recorded before and after the recording of NMDAR EPSCs and calculated the peak amplitude of averaged AMPAR EPSCs. To quantify NMDAR EPSCs, we averaged NMDAR EPSC traces and measured the mean amplitudes of the averaged NMDAR EPSCs between 47.5 ms and 52.5 ms after the onset of photostimulation. As AMPAR EPSCs completely disappeared 50 ms after photostimulation, EPSCs recorded at +40 mV 50 ms after stimulation onset were not contaminated with AMPAR EPSCs and reflected NMDAR EPSCs^7^. Then, we calculated the amplitude ratio of AMPAR EPSCs to NMDAR EPSCs.

Paired-pulse ratio: In order to calculate the paired-pulse ratio (PPR), AMPAR EPSCs were evoked by paired photostimulation (50 ms interval, 0.5 ms duration) of Chronos-expressing presynaptic axons and recorded in BA neurons at −80 mV in voltage-clamp mode. PPR was calculated as the peak amplitude ratio of the first to the second EPSC. As the PPR was affected by photostimulation intensity, we induced EPSCs with photostimulation at different light intensities and calculated the PPR for each photostimulation intensity in each BA neuron.

Progressive block of NMDAR EPSCs by MK-801: To compare presynaptic release probability in the vCA1 inputs to the BA between behavioral groups, we recorded NMDAR EPSCs at +40 mV in voltage-clamp mode in the presence of NBQX (10 μM) and SR-95531 (10 μM). Photostimulation intensity was adjusted such that the peak amplitude of NMDAR EPSC was 200–500 pA. After baseline recording, the brain slice was perfused with ACSF containing MK-801 (10 μM) for 10–15 min. Then, we recorded NMDAR EPSCs evoked by photostimulation applied every 10 s. To quantify the rate of NMDAR EPSC decay by MK-801, we calculated decay constant (τ) in stimulus number for each BA neuron by fitting the curve of NMDAR EPSC decrease to a single-exponential equation, *I*(*n*) = *I*_1_ *exp*(−*n*/τ), where *n* is stimulus number, *I*(*n*) is the peak amplitude of the *n*th NMDAR EPSC, and *I*_1_ is the peak amplitude of the first NDMAR EPSC recorded in the presence of MK-801.

### Reproducibility

Micrographic images presented in figures are representative ones from experiments repeated independently: Fig. 1b (10 times), 1d (18 times), 1f (9 times), 2b (13 times), 3b (5 times), 3e (6 times per group), 3h (8 times), 3j (5 times), 5d (5 times), 5e (18 times per group), 6d (3 times), 6i (11 times/BA; 13 times/vCA1), 7c–d (5 times), 7e (12 times per group), 8e–f (10 times/FC; 9 times/HC), 9a (12 times), 9d (7 times), 10d–e (6 times), 10f (11 times per group), 10k–l (5 times), 10m (10 times/mCh−; 12 times/mCh+), Supplementary Figs 2b (12 times), 3b (6 times), 4c–4d (5 times), 5a (3 times), 7a (5 times), 7c (3 times), 8a (5 times), 8b (22 times), 8c (5 times), 10d (12 times), 14c–d (5 times per group), 16a (6 times), 17b (6 times/Fos-CreERT2; 3 times/Arc-CreERT2), 17e (3 times), 17f (4 times per group), 18c (10 times/FC; 9 times/HC), 19b (3 times), 19c (17 times per group), and 20b (5 times).

### Statistical analysis

Data are presented as the means ± the standard error of the mean (SEM) unless indicated otherwise. For statistical comparisons, we used Welch’s *t*-test or ordinary or repeated measures ANOVA. For post hoc analysis, we used Bonferroni’s simultaneous comparisons. In Fig. 4e, k and Supplementary Figs. 6f, 10e, 11c, nonparametric statistics were used as data did not follow a normal distribution (*p* < 0.05, Anderson-Darling test). All statistical tests were two-sided. Statistical analysis was performed with Minitab 18 software (Minitab), and *p* < 0.05 was considered statistically significant. Details of the statistical analyses are summarized in Supplementary Tables 1 and 2.

### Reporting Summary

Further information on research design is available in the Nature Research Reporting Summary linked to this article.

## Supplementary information

Supplementary Information

Reporting Summary

## Data Availability

All data reported in this study are available from the corresponding authors upon request. The source data underlying all Figures and Supplementary Figures are provided as a Source Data file.

## Source data

Source Data
