# Supplementary material for: Encoding of contextual fear memory in hippocampal–amygdala circuit
Source: Nat Commun. 2020 Mar 13;11:1382. doi: 10.1038/s41467-020-15121-2 (PMC7069961; doi:10.1038/s41467-020-15121-2)
Supplement: Supplementary file 1 — Supplementary Information [file 41467_2020_15121_MOESM1_ESM.pdf]

SUPPLEMENTARY INFORMATION

Encoding of Contextual Fear Memory in Hippocampal–Amygdala Circuit

Woong Bin Kim and Jun-Hyeong Cho*

Nature Communications

SUPPLEMENTARY FIGURES AND LEGENDS

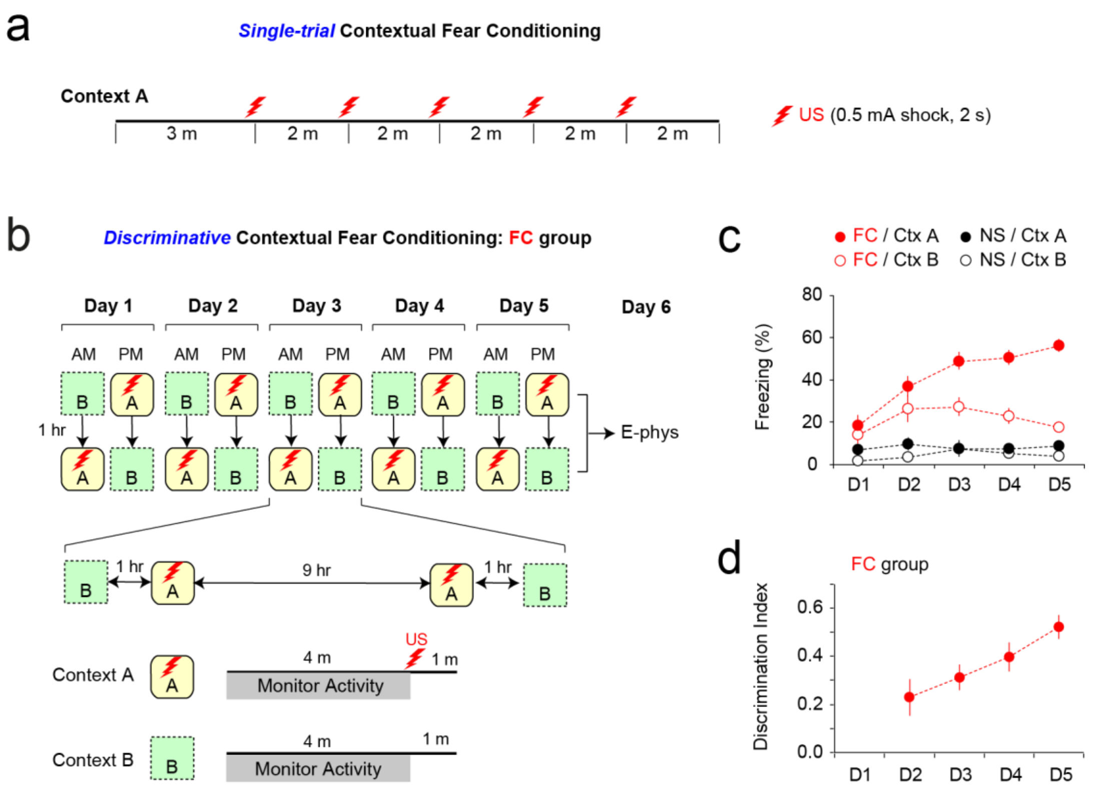

Supplementary Figure 1. Behavioral protocols and quantification of freezing behavior during discriminative contextual fear conditioning

(a) Diagram showing a behavioral protocol used for ***single-trial*** contextual fear conditioning in **Fig. 1, 6h-j, and 9** and for labeling BA fear neurons in **Fig. 5, 7, and 10**. Mice received five electric footshocks (0.5 mA, 2 s duration) at 2-minute intervals in Context A.

(b) Diagrams showing a behavioral protocol used for ***multiple-trial*** discriminative contextual fear conditioning in **Fig. 4 and 5**. On the training days (Days 1-5), mice in the fear conditioning (FC) group were exposed to Context B for 5 minutes between 9 AM and 10 AM. After 1 hour, the mice were placed in Context A and given a US (electric footshock, 0.5 mA, 2 s duration) 4 minutes later. On the same days, the mice received a US in Context A between 6 PM and 7 PM and then were exposed to Context B without the US an hour later. In each training session, mice were tested for freezing behavior for the first 4 minutes in Contexts A or B. On Day 6, brain slices were prepared from these mice for electrophysiological recording experiments. Mice in the no shock (NS) control group were similarly exposed to Contexts A and B on Days 1-5 but never received the US.

(c) Freezing behavior in Context (Ctx) A and Context B during discrimination fear conditioning as in (b). Mice in the no shock (NS) control group were exposed to the contexts as in the FC group but without a shock. n = 11 mice per group.

(d) Plot of the discrimination index on Days 2-5 in the FC group during multiple-trial discriminative contextual fear conditioning as in (b). The fear discrimination index (DI) was calculated using the following equation: DI = (Context A freezing – Context B freezing) / (Context A freezing + Context B freezing). n = 11 mice.

Error bars represent standard error of the mean (SEM). Source data are provided as a Source Data file.

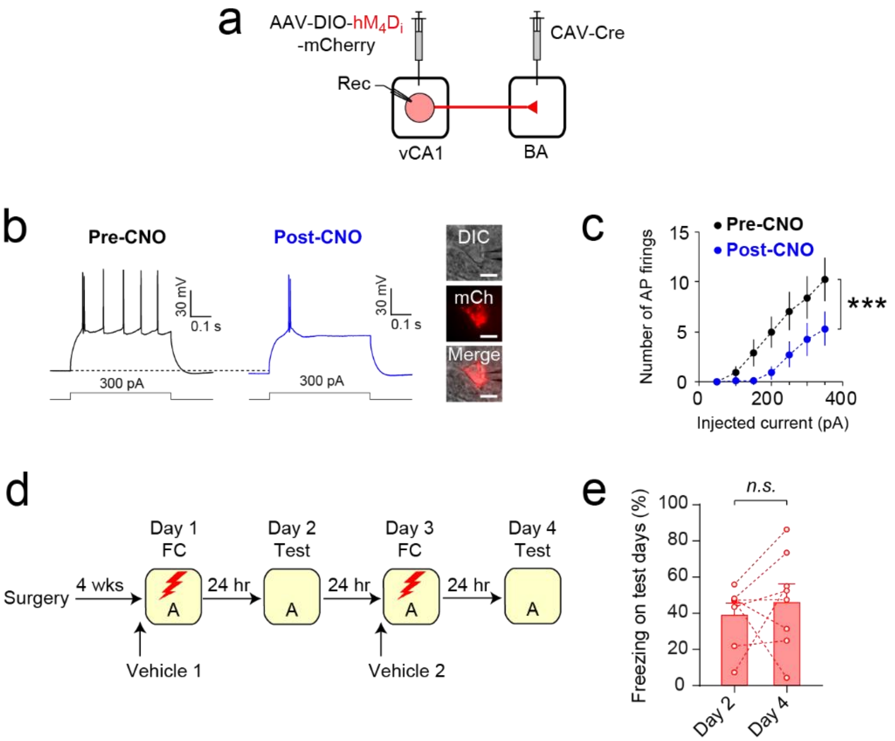

Supplementary Figure 2. Chemogenetic silencing of vCA1 neurons projecting to the BA: control experiments

(a) Experimental setup for (b)-(c). vCA1 neurons projecting to the BA (vCA1: BA projectors) expressed hM_4D_i-mCherry. Retrograde CAV2-Cre was bilaterally injected into the BA. AAV-DIO-hM_4D_i-mCherry was bilaterally injected into the vCA1.

(b) Traces of AP firing before (pre-CNO) and 5 minutes after CNO application (10 μM, post-CNO). AP firing was induced by depolarizing current injection (500 ms long) and recorded in an hM_4D_i-mCherry(mCh)-expressing vCA1 neuron (inset; scale bar, 10 μm) in current-clamp mode. A dotted line indicates membrane potential before CNO application. Note the hyperpolarization after CNO application.

(c) Summary plot of AP firing in hM4Di-expressing vCA1 neurons (n = 12 cells). *** ***p*** < 0.001 (two-way ANOVA with ***post hoc*** comparisons).

(d) Behavioral training and testing protocols for (e). vCA1: BA projectors expressed hM_4D_i-mCherry as in (a). Four weeks after surgery, the mice received a vehicle injection 30 minutes before fear conditioning in Context A on Day 1 (vehicle 1) and Day 3 (vehicle 2). The mice were tested for fear memory in Context A on Days 2 and 4.

(e) Comparison of freezing behavior in Context A on Day 2 versus Day 4. There was no significant difference in freezing behavior on Day 2 versus Day 4 (***n.s.***, ***p*** = 0.50, two-sided paired ***t***-test; 8 mice), suggesting that the CNO effect in the hM_4D_i group in **Fig. 1g-h** was not due to the order of CNO and vehicle injections before fear conditioning.

Error bars indicate the SEM. Source data are provided as a Source Data file.

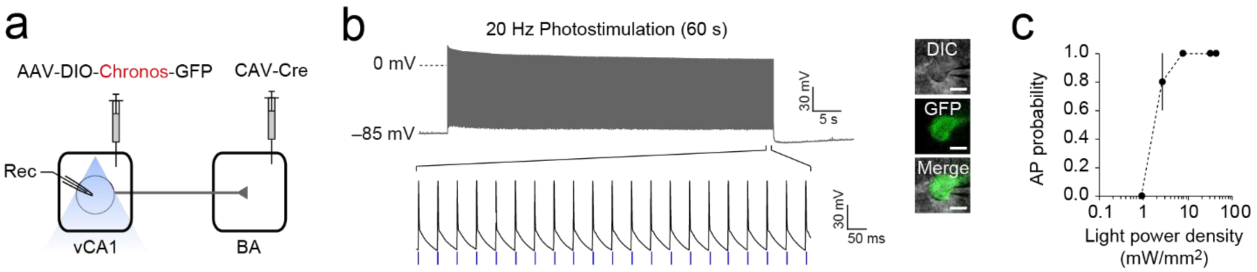

Supplementary Figure 3. Optogenetic stimulation of vCA1 neurons projecting to the BA

(a) For Chronos-GFP expression in vCA1 neurons projecting to the BA, retrograde CAV2-Cre was injected into the BA, and AAV-DIO-Chronos-GFP was injected into the vCA1.

(b) ***Top***: AP firing induced by photostimulation at 20 Hz (1 ms pulses) and recorded in a Chronos-GFP-expressing vCA1 neuron (inset; scale bar, 10 μm) in a brain slice. ***Bottom***: a magnified view of the AP trace for the last 1 s of 60 s photostimulations (blue vertical bars).

(c) Quantification of AP firing probability in Chronos-expressing vCA1 neurons (n = 6 cells). AP firings was induced as in (b).

Error bars indicate the SEM. Source data are provided as a Source Data file.

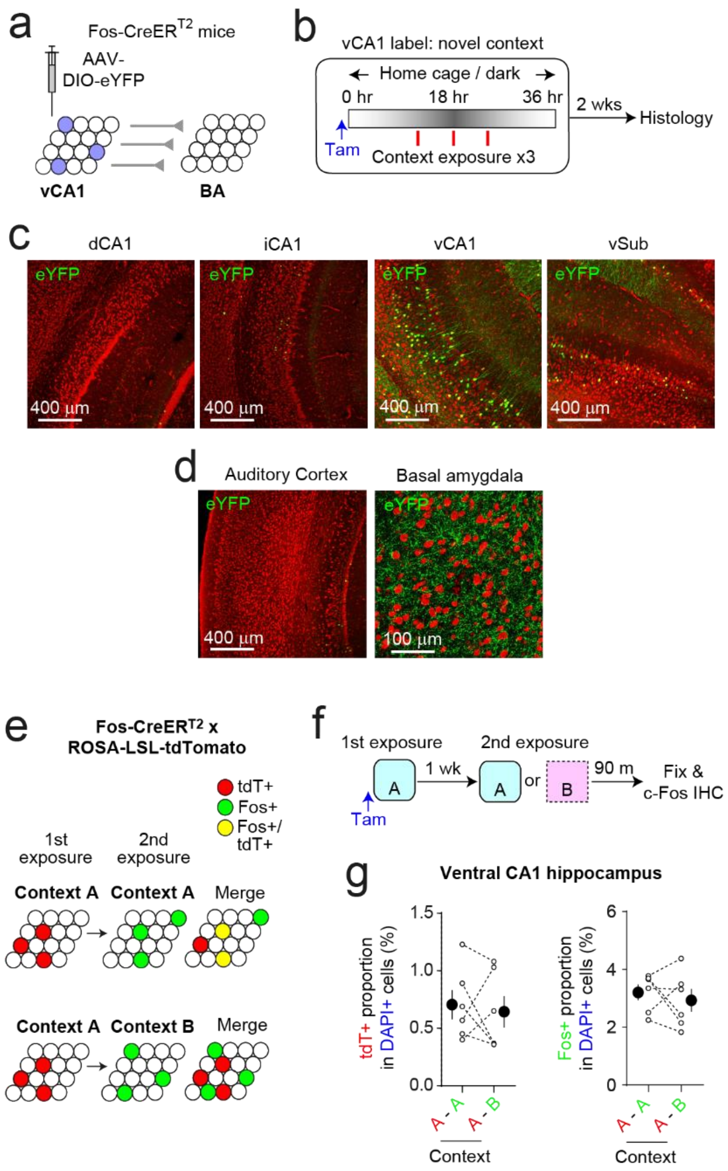

Supplementary Figure 4. **Transgene expression in CA1 hippocampal neurons in Fos-CreER^{T2} mice.**

(a) Experimental setup for (b)-(d). AAV-DIO-eYFP was injected targeting the vCA1 in Fos-CreER^{T2} mice.

(b) Mice were exposed to a novel context (red bars) after tamoxifen injection (Tam) to label vCA1 neurons.

(c) eYFP+ neurons (green) in subdivisions of hippocampal CA1 areas. eYFP+ neurons were predominantly found in the vCA1. Red, Nissl stain. dCA1, iCA1, vCA1, and vSub: the dorsal, intermediate, ventral CA1 hippocampus, and ventral subiculum, respectively.

(d) Images showing the auditory cortex with no eYFP-labeled soma (left) and the basal amygdala with eYFP+ axons of labeled vCA1 neurons (right). Red, Nissl stain.

(e)-(f) Experimental setup for (g). After tamoxifen injection, mice were exposed to Context A to label with tdTomato vCA1 neurons active in Context A as in **Fig. 3e**. Mice were then exposed to Context A (A–A group) or Context B (A–B group) before brain fixation for c-Fos immunostaining (c-Fos IHC).

(g) Comparisons of the proportion of tdTomato+ cells among all DAPI+ vCA1 cells ($\boldsymbol{p}$ = 0.69) and the proportion of c-Fos+ cells among all DAPI+ vCA1 cells ($\boldsymbol{p}$ = 0.53). Two-sided paired $\boldsymbol{t}$-tests were used. n = 6 mice per group.

Error bars indicate the SEM. Source data are provided as a Source Data file.

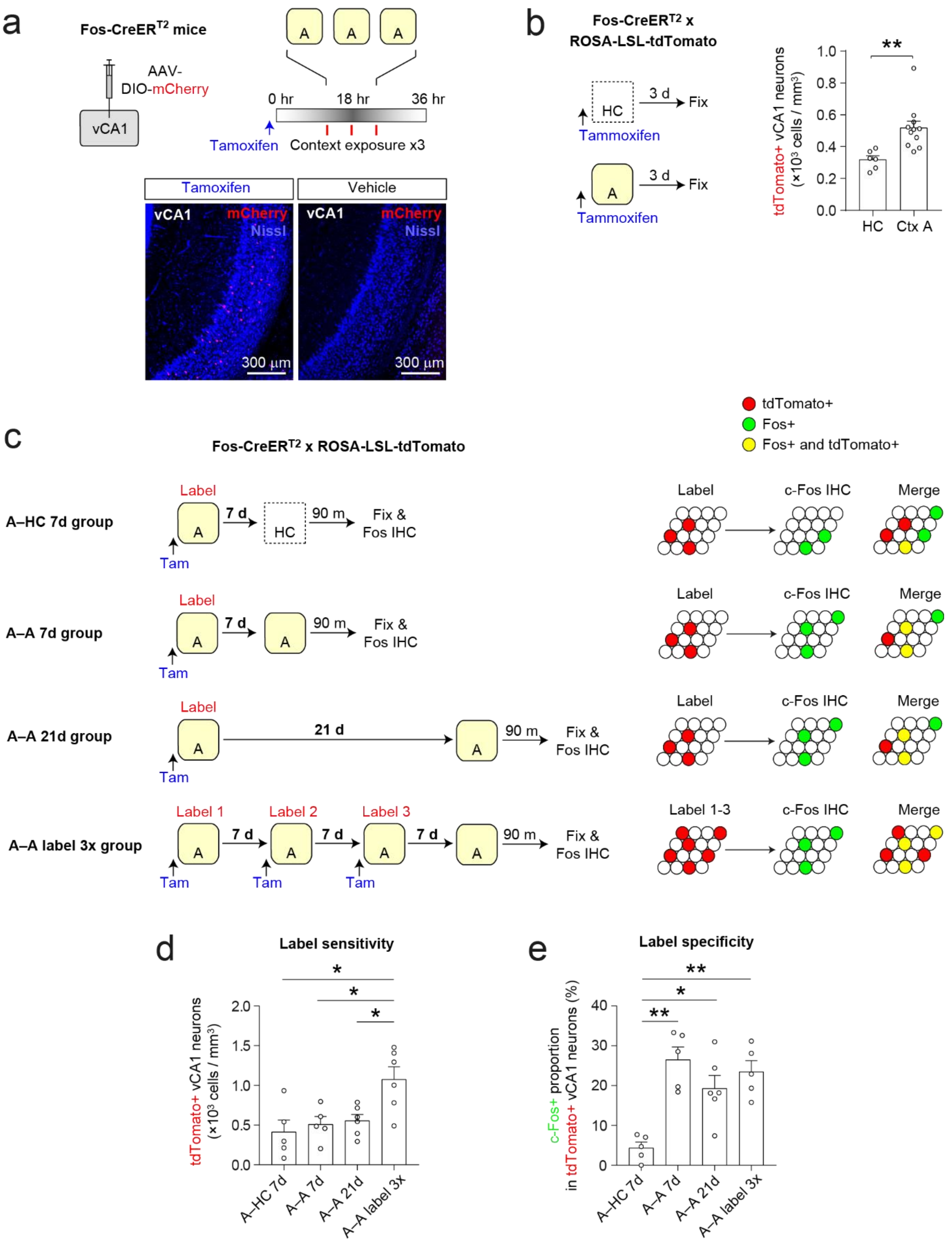

Supplementary Figure 5. **Neural activity-dependent labeling of vCA1 neurons in Fos-CreER^{T2} mice**

(a) ***Top***: AAV-DIO-mCherry was injected into the vCA1 in Fos-CreER^{T2} mice. After intraperitoneal injection of tamoxifen or vehicle, mice were exposed to novel Context A three times for 12 minutes each to label vCA1 neurons active in Context A as in **Fig. 3b**.

Bottom: microscopic images showing mCherry expression (red) in a vCA1 neuronal population in mice that received tamoxifen before the labeling procedure but not in mice that were injected with vehicle.

(b) More vCA1 neurons were labeled in mice exposed to a novel context after tamoxifen injection than in mice that remained in the home cages. ***Left:*** after tamoxifen injection, Fos-CreER^{T2} x Rosa-LSL-tdTomato mice in the Ctx A group (11 mice) were exposed to Context A for tdTomato expression in vCA1 neurons active in Context A as in **Fig. 3b**. Mice in the HC group (6 mice) remained in the home cages for tdTomato expression in vCA1 neurons active in the home cages. After 3 days, the brain tissue was fixed for microscopic imaging. ***Right***: the density of tdTomato+ neurons in the vCA1 was significantly higher in the Ctx A group than in the HC group (** p = 0.001, two-sided unpaired t-test).

(c) ***Left:*** experimental setup for (d)-(e). After tamoxifen injection, Fos-CreER^{T2} x Rosa-LSL-tdTomato mice were exposed to Context A to label with tdTomato vCA1 neurons active in Context A as in **Fig. 3b**. Seven days after labeling, mice in the A–A 7d group (5 mice) was exposed to Context A, whereas mice in the A–HC 7d group (5 mice) remained in the home cages. The brain tissue was fixed 90 minutes later for c-Fos immunohistochemistry. Mice in the A–A 21d group (6 mice) were exposed to Context A 21 days after initial labeling, and the brain tissue was fixed 90 minutes later for c-Fos immunostaining. Mice in the A–A label 3x group (6 mice) received two additional context labeling sessions with a 1-week interval (label 2 and 3). One week after the last labeling session, they were exposed to the same Context A 90 minutes before brain fixation for c-Fos immunostaining.

Right: vCA1 neurons active in Context A were labeled with tdTomato during the first context exposure in the A–HC 7d, A–A 7d, and A–A 21d groups and during the first three context labeling sessions in the A–A label 3x group (tdTomato+, red). The last Context A exposure in the A–A 7d, A–A 21d, and A–A label 3x group activated a subset of vCA1 neurons, which were immunostained for c-Fos, whereas vCA1 neurons active in the home cages were immunostained for c-Fos in the A–HC 7d group (Fos+, green).

(d) Comparison of the density of tdTomato-labeled vCA1 neurons (i.e., label sensitivity) between groups. Note that more vCA1 neurons were labeled with tdTomato in the A–A label 3x group than in other groups (* $p < 0.05$, one-way ANOVA with ***post hoc*** comparisons).

(e) Comparison of the proportion of c-Fos+ proportion among all tdTomato-labeled vCA1 neurons (i.e., label specificity) between groups. Note a higher proportion of reactivated vCA1 neurons during the last Context A exposure in the A–A 7d, A–A 21d group, and A–A/label 3x groups as compared with the A–HC 7d group (* $p <$ 0.05, ** $p < 0.01$, one-way ANOVA with ***post hoc*** comparison). There was no significant difference in the proportion between the A–A 7d, A–A 21d, and A–A label 3x groups.

Error bars represent the SEM. Source data are provided as a Source Data file.

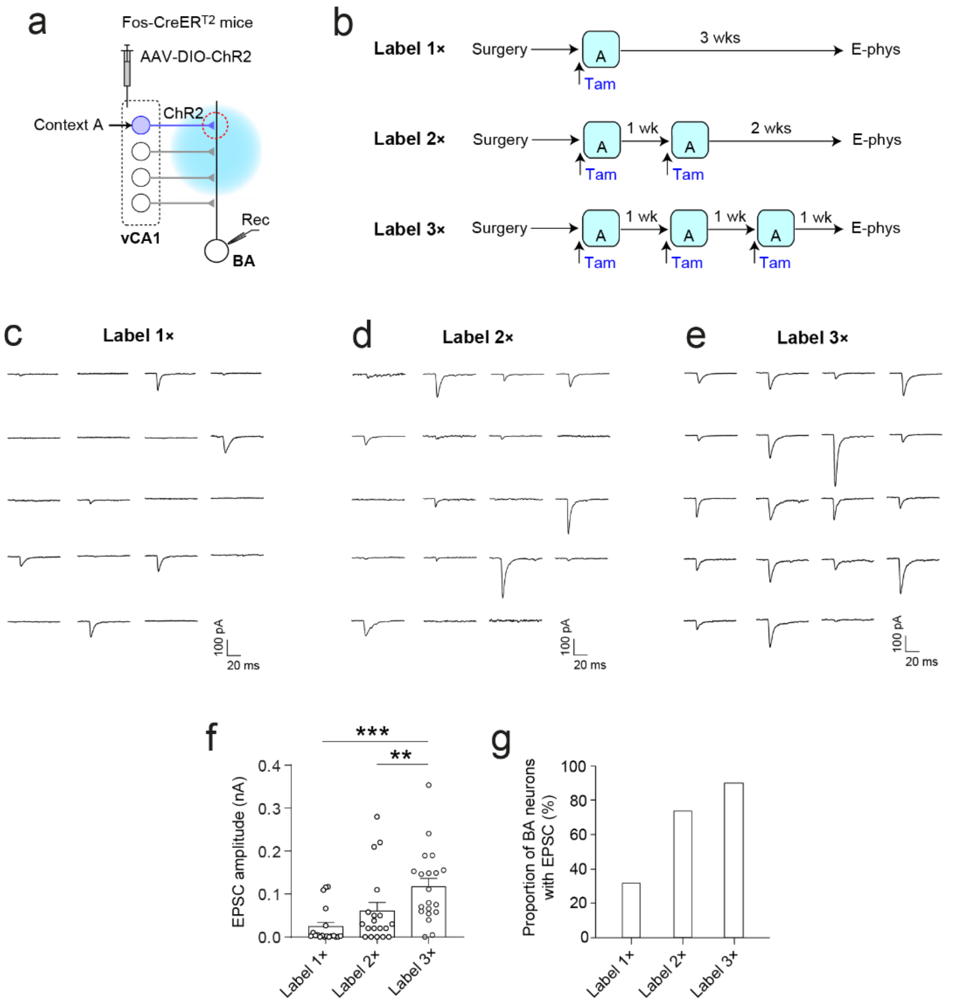

Supplementary Figure 6. Comparison of EPSCs in Context A vCA1–BA pathway after single versus multiple context labeling sessions in Fos-CreER^{T2} mice

(a) Experimental setup. AAV-DIO-ChR2-eYFP was injected into the vCA1 in Fos-CreER^{T2} mice. Context A exposure after tamoxifen injection induced ChR2-eYFP expression in vCA1 neurons active in Context A. Photostimulation activated ChR2-expressing axons and induced postsynaptic responses in BA neurons (Rec).

(b) A week after virus injection surgery, mice in Label 1x group received a tamoxifen injection and exposed to Context A as in **Fig. 3b**. Mice in Label 2x group underwent one additional context labeling session, whereas mice in Label 3x group underwent two additional labeling sessions with a 1-week interval as in **Fig. 3h**. Recording experiments were performed three weeks after the first labeling session.

(c) EPSC traces in the Label 1x group. Each EPSC trace indicates synaptic responses recorded in individual BA neurons (n = 19 neurons). EPSCs were induced by photostimulation of the same intensity (20.0 mW/mm^2) and recorded in randomly selected BA neurons.

(d) EPSC traces in the Label 2x group (n = 19 neurons).

(e) EPSC traces in the Label 3x group (n = 19 neurons).

(f) Comparison of the average EPSC amplitude between groups (** $\boldsymbol{p}$ = 0.008, *** $\boldsymbol{p}$ =0.00008, two-sided Kruskal-Wallis multiple comparisons). Open circles indicate the peak amplitude of EPSC recorded in each BA neuron. n = 19 cells for Label 1x and Label 2x groups. n = 20 cells for Label 3x group.

(g) Comparison of the proportion of BA neurons with EPSC among all examined BA neurons. n = 19 cells for Label 1x and Label 2x groups. n = 20 cells for Label 3x group.

Error bars represent the SEM. Source data are provided as a Source Data file.

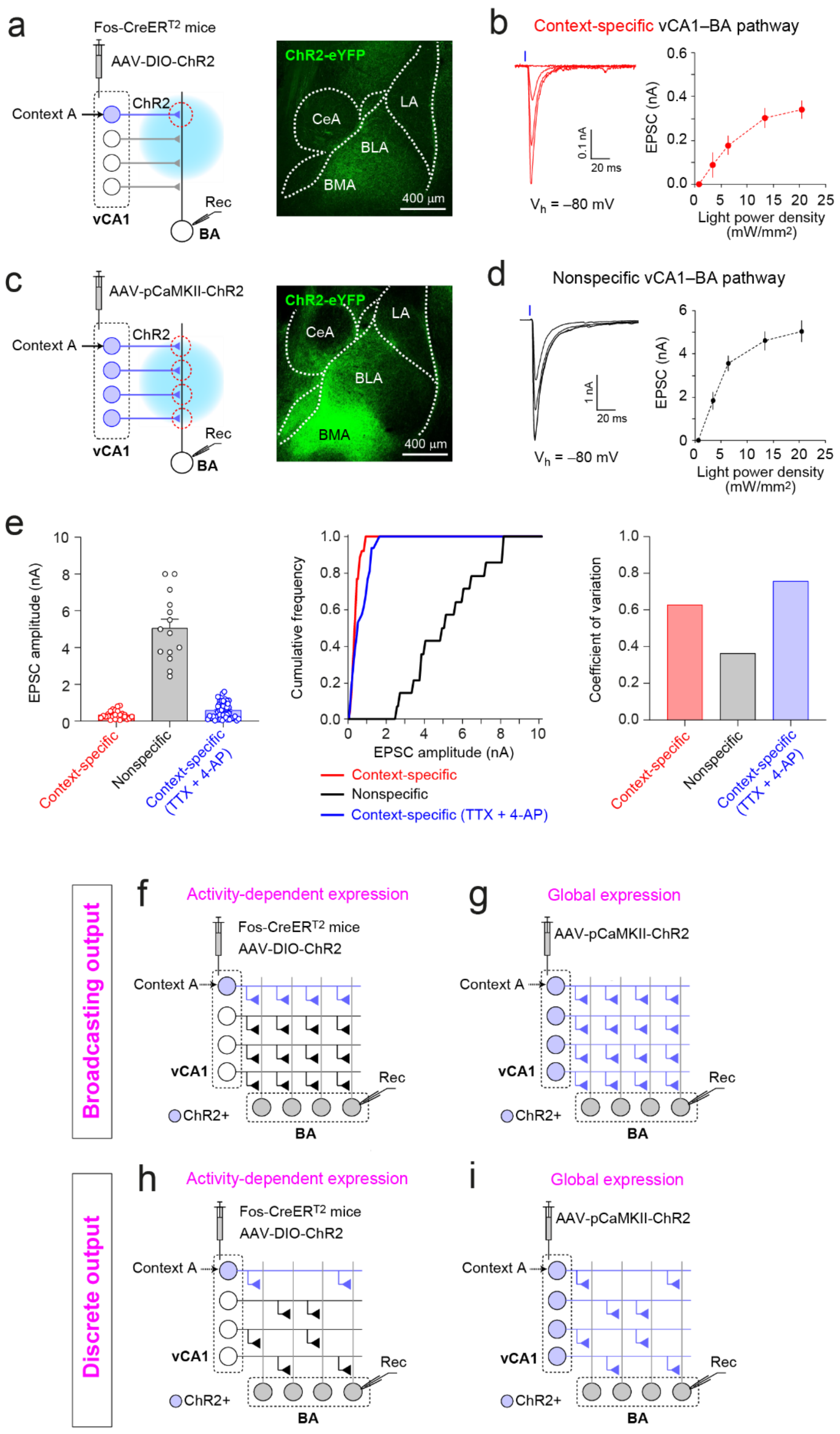

Supplementary Figure 7. Comparison of EPSCs induced by stimulation of context-specific versus randomly selected vCA1 inputs

(a) ***Left***: experimental setup for (b). AAV-DIO-ChR2-eYFP was injected into the vCA1 in Fos-CreER^{T2} mice. Context A exposure after tamoxifen injection induced ChR2-eYFP expression in vCA1 neurons active in Context A. ***Right***: microscopic image showing ChR2-eYFP-expressing axons of labeled vCA1 neurons in the BA (green).

(b) ***Left***: representative traces of EPSCs induced by selective photostimulation of the Context A vCA1 inputs and recorded in a BA principal neuron at −80 mV. EPSCs were induced by photostimulation at four light intensities (3.4, 6.4, 13.4, and 20.0 mW/mm^2). ***Right***: plot of the average amplitude of EPSCs in the Context A vCA1–BA pathway versus photostimulation intensity. BA neurons, in which with photostimulation did not induced EPSC, were excluded from analysis. n = 26 neurons from 5 mice.

(c) ***Left***: experimental setup for (d). AAV-pCaMKIIα-ChR2-eYFP was injected into the vCA1, which globally expressed ChR2-eYFP under the control of the CaMKIIα promoter. ***Right***: microscopic image showing ChR2-eYFP-labeled vCA1 projections to the BA (green).

(d) ***Left***: representative traces of EPSCs induced by global photostimulation of the vCA1–BA pathways and recorded in a BA neuron as in (c). ***Right***: plot of the average amplitude of EPSCs in nonspecific vCA1–BA pathways versus photostimulation intensity. n = 14 neurons from 3 mice.

(e) ***Left***: comparisons of EPSC amplitude between groups. EPSCs in the context-specific vCA1–BA pathway were induced with photostimulation (20.0 mW/mm^2) and recorded as in (c) (n = 26 neurons), whereas EPSCs in nonspecific vCA1–BA synapses induced with the same photostimulation and were recorded as in (d) (n = 14 neurons). In the context-specific (TTA + 4-AP) group, EPSC were recorded in the context-specific vCA1–BA pathway in the presence of TTX and 4-AP as in **Fig. 3m-n** (n = 47 neurons). ***Middle***: cumulative histograms of the EPSC amplitude. ***Right***: comparison of the coefficient of variation (the ratio of the standard deviation to the mean).

(f-g) ***Broadcasting output model***. Context-specific vCA1 neurons project diffusely to most BA neurons with axon collaterals. ChR2 is expressed selectively in vCA1 neurons active in Context A (f) or globally expressed in the vCA1 (g). Although EPSCs recorded in BA neurons (Rec) are smaller in (f) than in (g), the variability of EPSCs in (f) is comparable to EPSC variability in (g) as most BA neurons receive inputs from Context A vCA1 neurons to the same extent.

(h-i) ***Discrete output model***. Context-specific vCA1 neurons project only to a subset of BA neurons, although the total number of vCA1 inputs is uniform in each BA neuron. ChR2 is selectively expressed in vCA1 neurons active in Context A in (h), whereas ChR2 is expressed globally in the vCA1 in (i). EPSCs recorded in BA neurons are smaller in (h) than in (i). The variability of EPSCs recorded in different BA neurons is larger in (h) than in (i) as only a subset of BA neurons receives inputs from Context A vCA1 neurons. Our results in (a)-(e) support the discrete output model.

Error bars are the SEM. Source data are provided as a Source Data file.

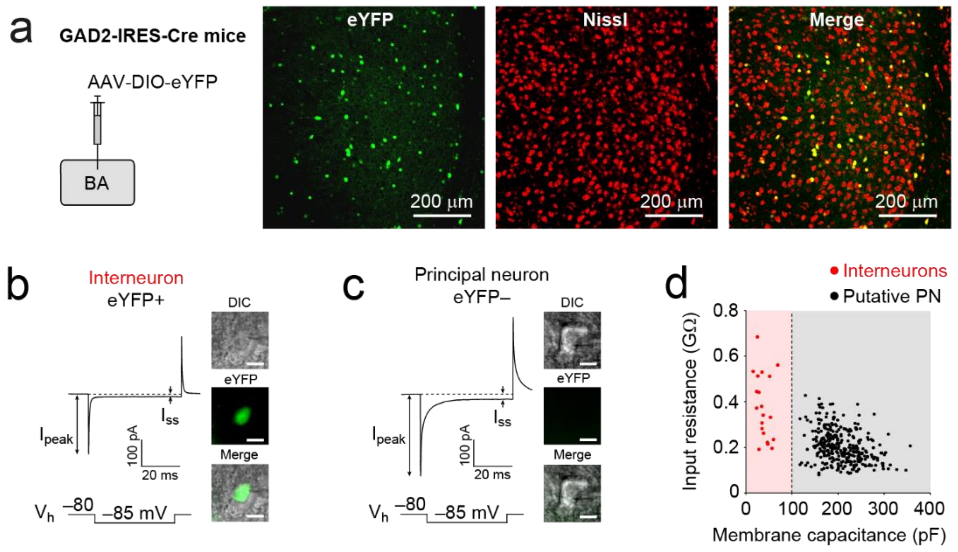

Supplementary Figure 8. Passive membrane properties of BA neurons

(a) Microscopic image showing eYFP-labeled GABAergic interneurons (green) in the BA in GAD2-IRES-Cre mice, which received AAV-DIO-eYFP injection into the BA.

(b) Trace representing the average of 50 current traces induced by hyperpolarizing voltage pulses and recorded in voltage-clamp mode with a sampling rate of 100 kHz in an eYFP-labeled interneuron in the BA (inset; scale bar, 10 μm). The peak amplitude of the transient capacitive current ($\boldsymbol{I}_{peak}$) was used to estimate apparent access resistance ($\boldsymbol{R}_a$) using the following equation: $\boldsymbol{R}_a = -5\ \text{mV} / \boldsymbol{I}_{peak}$. Steady-state current ($\boldsymbol{I}_{ss}$) is the average current from the baseline for the last 5 ms of the voltage pulse and was used to calculate input resistance ($\boldsymbol{R}_{in}$) using the following equation: $\boldsymbol{R}_{in} = (-5\ \text{mV} / \boldsymbol{I}_{ss}) - \boldsymbol{R}_a$. Transient capacitive current was fitted with a double exponential function, $\boldsymbol{I}(\boldsymbol{t}) = \boldsymbol{I}_f \exp(-\boldsymbol{t}/\tau_f) + \boldsymbol{I}_s \exp(-\boldsymbol{t}/\tau_s)$, where $\boldsymbol{I}_f/\tau_f$ and $\boldsymbol{I}_s/\tau_s$ are the fast and slow components of the peak amplitudes and the decay time constants of the transient capacitive current. The weighted mean decay time constant (τ_w) was calculated with the equation $\tau_w = \tau_f [\boldsymbol{I}_f/(\boldsymbol{I}_f + \boldsymbol{I}_s)] + \tau_s [\boldsymbol{I}_s/(\boldsymbol{I}_f + \boldsymbol{I}_s)]$ and was used to calculate membrane capacitance ($\boldsymbol{C}_m$) with the equation $\boldsymbol{C}_m = \tau_w \times (\boldsymbol{R}_a + \boldsymbol{R}_{in}) / (\boldsymbol{R}_a \times \boldsymbol{R}_{in})$.

(c) Trace representing the average of 50 current traces induced by hyperpolarizing voltage pulses as in (b) and recorded in a putative principal neuron in the BA (eYFP–, inset; scale bar, 10 μm).

(d) Scatter plot of membrane capacitance and input resistance calculated in eYFP-labeled GABAergic interneurons (red, 22 cells) and putative principal neurons (PN) examined in **Fig. 4 and 5** (black, 349 cells). As membrane capacitance was less than 100 pF (dotted vertical line) in all the interneurons examined, recorded neurons with a membrane capacitance larger than 100 pF were regarded as principal neurons.

Source data are provided as a Source Data file.

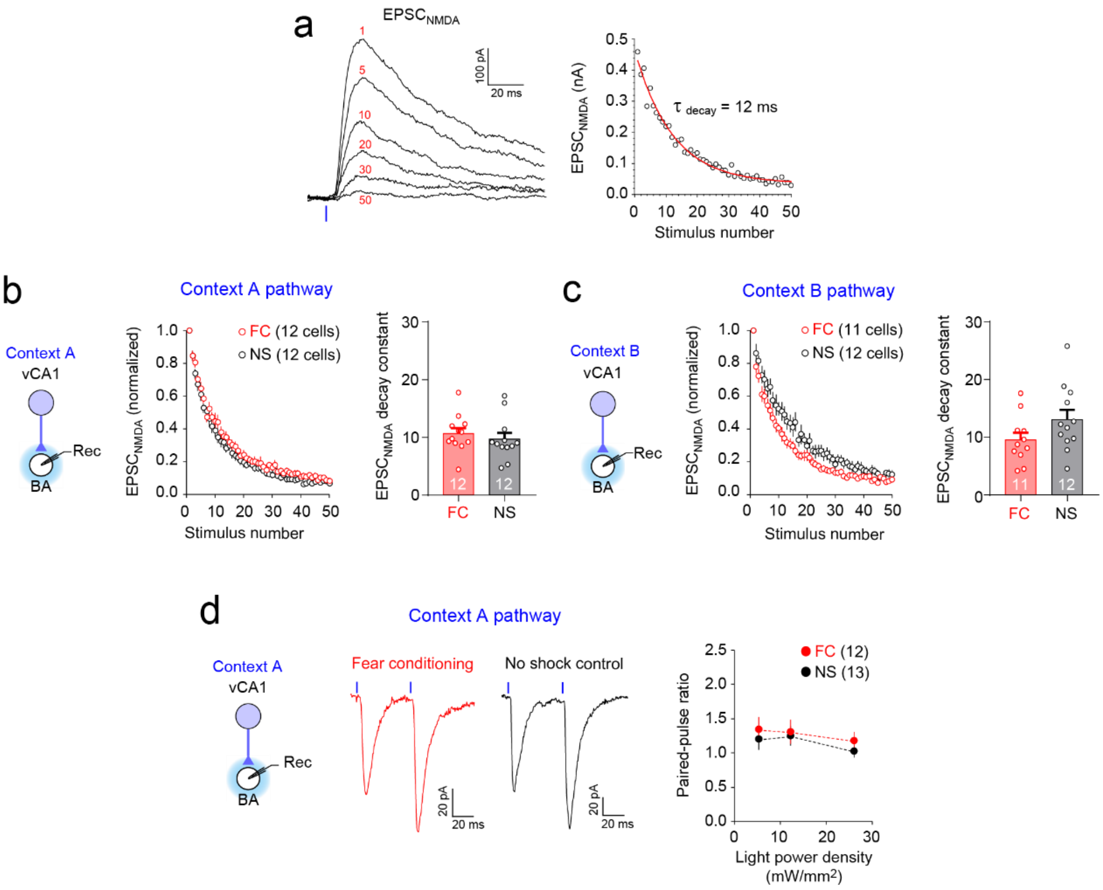

Supplementary Figure 9. Comparison of presynaptic function in the vCA1–BA pathway between groups

(a) ***Left***: representative traces of NMDA receptor (NMDAR)-mediated EPSCs evoked by the ***n***th photostimulation after MK-801 application (10 μM) for 10-15 minutes, showing progressive block of NMDAR EPSCs by MK-801. EPSCs were induced every 10 s by photostimulation (blue bar) of ChR2-expressing vCA1 axons and recorded in a BA neuron at +40 mV in voltage-clamp mode in the presence of NBQX (10 μM) and SR-95531 (10 μM). ***Right***: plot showing a gradual decrease in the peak amplitude of NMDAR EPSCs. The peak amplitude of NMDAR EPSCs was plotted against stimulation number (open circles). The decay constant (τ_{decay}) in stimulus number was calculated by fitting the curve of NMDAR EPSC decrease to single-exponential equations (red curve): $I(n) = I_1 \exp(-n/\tau)$, where ***n*** is stimulus number, $I(n)$ is the peak amplitude of the ***n***th NMDAR EPSC, and I_1 is the peak amplitude of the first NMDAR EPSC recorded in the presence of MK-801.

(b) ***Left***: NMDAR EPSCs were induced by photostimulations of ***Context A*** vCA1 inputs as in **Fig. 4a-b** and recorded in BA neurons as in (a). ***Middle***: plot showing a gradual decrease in NMDAR EPSC amplitude. The peak amplitude of NMDAR EPSCs was normalized to the first EPSC induced after MK-801 application. ***Right***: quantification of the rate of NMDAR EPSC decay by MK-801. The decay constant (τ) was calculated in each BA neuron (open circles) as in (a). $p = 0.48$, two-sided unpaired ***t***-test.

(c) ***Left***: NMDAR EPSCs were induced by photostimulations of the ***Context B*** vCA1 inputs as in **Fig. 4g-h** and recorded in BA neurons as in (a). ***Middle***: plot showing a gradual decrease in NMDAR EPSC amplitude. ***Right***: quantification of the rate of NMDAR EPSC decay by MK-801. $p = 0.10$, two-sided unpaired ***t***-test.

(d) ***Left***: AAV-DIO-Chronos was injected into the vCA1 in Fos-CreER^{T2} mice. vCA1 neurons active in Context A were labeled with Chronos. EPSCs were induced by photostimulations of ***Context A*** vCA1 inputs and recorded in BA neurons. ***Middle:*** traces of EPSCs induced by paired photostimulations with a 50 ms interval. ***Right:*** there was no significant difference in paired-pulse ratio between the FC (12 cells) and NS groups (13 cells) (main effect of groups, $p = 0.21$; group × intensity interaction, $p = 0.81$; two-way ANOVA).

Error bars represent the SEM. Source data are provided as a Source Data file.

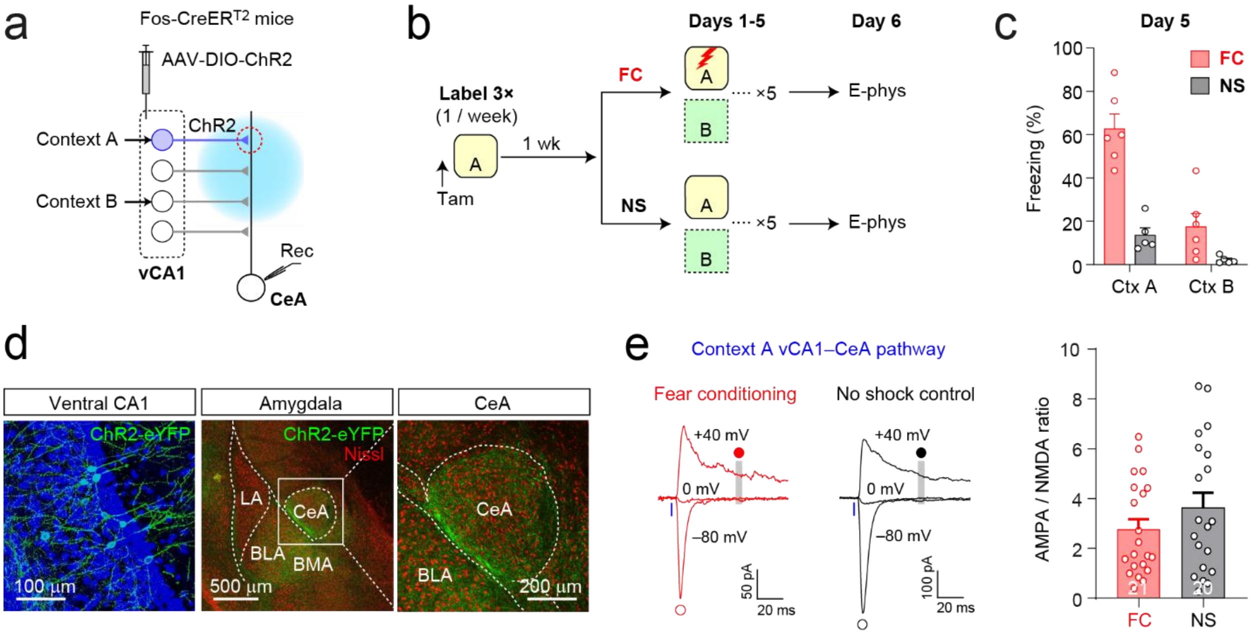

Supplementary Figure 10. Comparison of synaptic efficacy in Context A vCA1 inputs to CeA pathway

(a) Experimental setup (b)-(e). AAV-DIO-ChR2-eYFP was injected into the vCA1 in Fos-CreER^{T2} mice. EPSCs in Context A vCA1 inputs to the CeA were recorded and compared between groups.

(b) ***Left:*** mice were exposed to Context A to label vCA1 neurons active in the context as in **Fig. 4b**. After labeling, mice in the FC group were trained for discriminative fear in Context A on Days 1-5 as in **Supplementary Figure 1b**. Mice in the NS control group were exposed to the contexts as in the FC group but did not receive the US.

(c) Quantification of freezing behavior in the FC (6 mice) and NS groups (5 mice) on Day 5.

(d) Microscopic images showing ChR2-eYFP-expressing vCA1 neurons and their projections (green) in the amygdala. Blue, DAPI stain. Red, Nissl stain.

(e) ***Left:*** representative traces of EPSCs recorded in the Context A vCA1–CeA pathway in the FC (red traces) and NS groups (black traces). AMPAR and NMDAR EPSCs were induced and recorded as in **Fig. 4d**. ***Right***: comparison of the AMPA/NMDA ratio between groups. There was no significant difference in the AMPA/NMDA ratio in the Context A vCA1–CeA pathway between the FC (21 cells) and NS groups (20 cells) (p = 0.35, two-sided Mann-Whitney test).

Error bars represent the SEM. Source data are provided as a Source Data file.

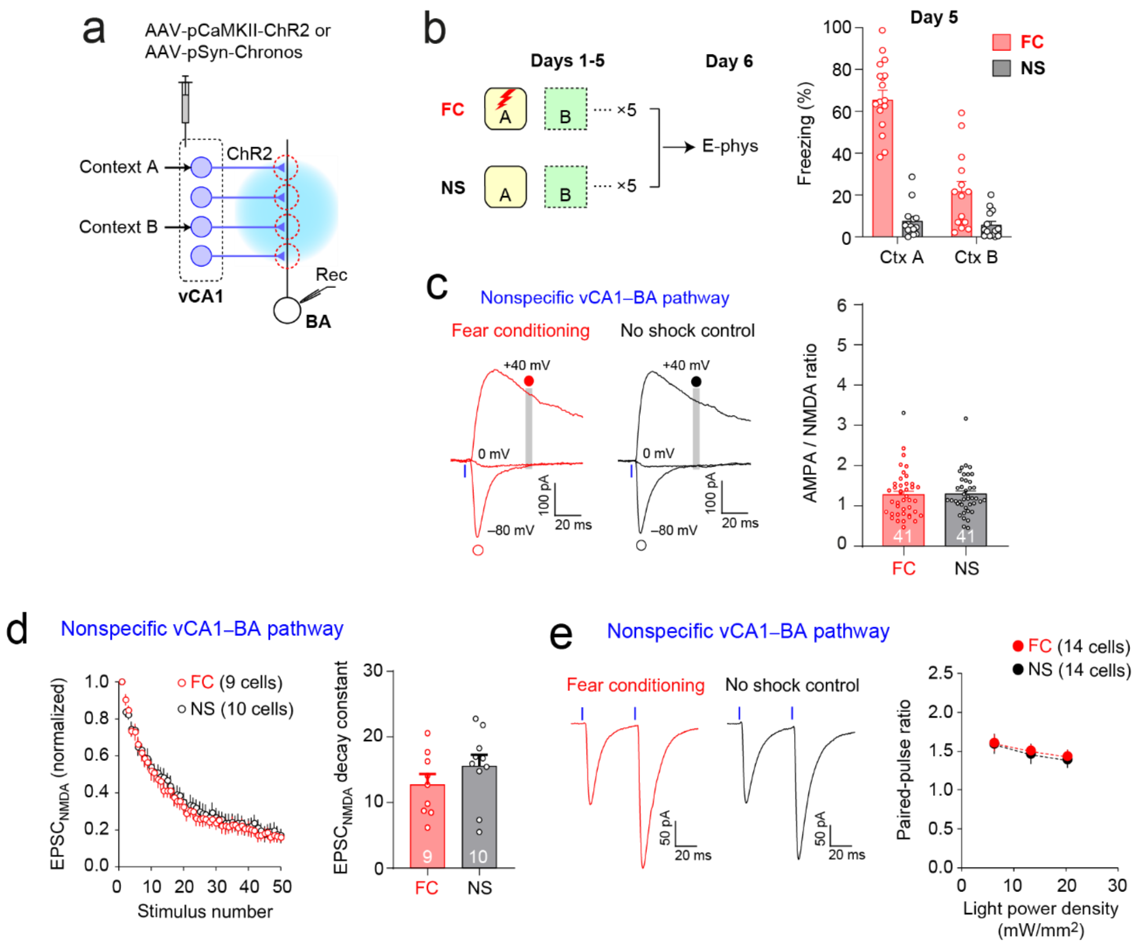

Supplementary Figure 11. Comparison of synaptic efficacy in nonspecific vCA1–BA pathway between behavioral groups

(a) Experimental setup. The vCA1 globally expressed ChR2 or Chronos. AAV-pCaMKII-ChR2 was injected into the vCA1 in (c)-(d), whereas AAV-pSyn-Chronos was injected into the vCA1 in (e). Photostimulation activated vCA1 axons and induced postsynaptic responses in BA neurons (Rec).

(b) ***Left:*** mice in the fear conditioning group (FC) were trained for discriminative fear in Context A as in **Supplementary Figure 1b**. Mice in the no shock control group (NS) were exposed to Contexts A and B as in the FC group but did not receive shocks. ***Right***: freezing behavior in Context (Ctx) A and Context B on Day 5. n = 14 mice per group.

(c) ***Left:*** EPSCs were induced by blue light illumination (1 ms pulses, blue bars), which globally activated vCA1 axons. EPSCs were recorded as in **Fig. 4d**. ***Right***: there was no significant difference in the AMPA/NMDA EPSC ratio between groups (***p*** = 0.66, two-sided Mann-Whitney test; n = 41 neurons per group).

(d) ***Left***: plot showing a gradual decrease in NMDAR EPSC amplitude in nonspecific vCA1−BA pathway in the FC and NS groups. NMDAR EPSCs were induced by photostimulations of nonspecific vCA1 axons. NMDAR EPSCs were recorded in BA neurons as in **Supplementary Figure 9a**. The peak amplitude of NMDAR EPSCs was normalized to the first EPSC induced after MK-801 application. ***Right***: quantification of the rate of NMDAR EPSC decay by MK-801. The decay constant (τ) in stimulus number was calculated in each BA neuron (open circles) as in **Supplementary Figure 9a**. There was no significant difference in the NMDAR EPSC decay constant between groups (***p*** = 0.25, two-sided unpaired ***t***-test).

(e) ***Left:*** traces of EPSCs induced by paired photostimulations of nonspecific vCA1 axons with a 50 ms interval. n = 14 cells from 3 mice for each group. ***Right:*** there was no significant difference in the paired-pulse ratio between groups (main effect of groups, ***p*** = 0.10; group × intensity interaction, ***p*** = 0.67, two-way ANOVA).

Error bars represent the SEM. Source data are provided as a Source Data file.

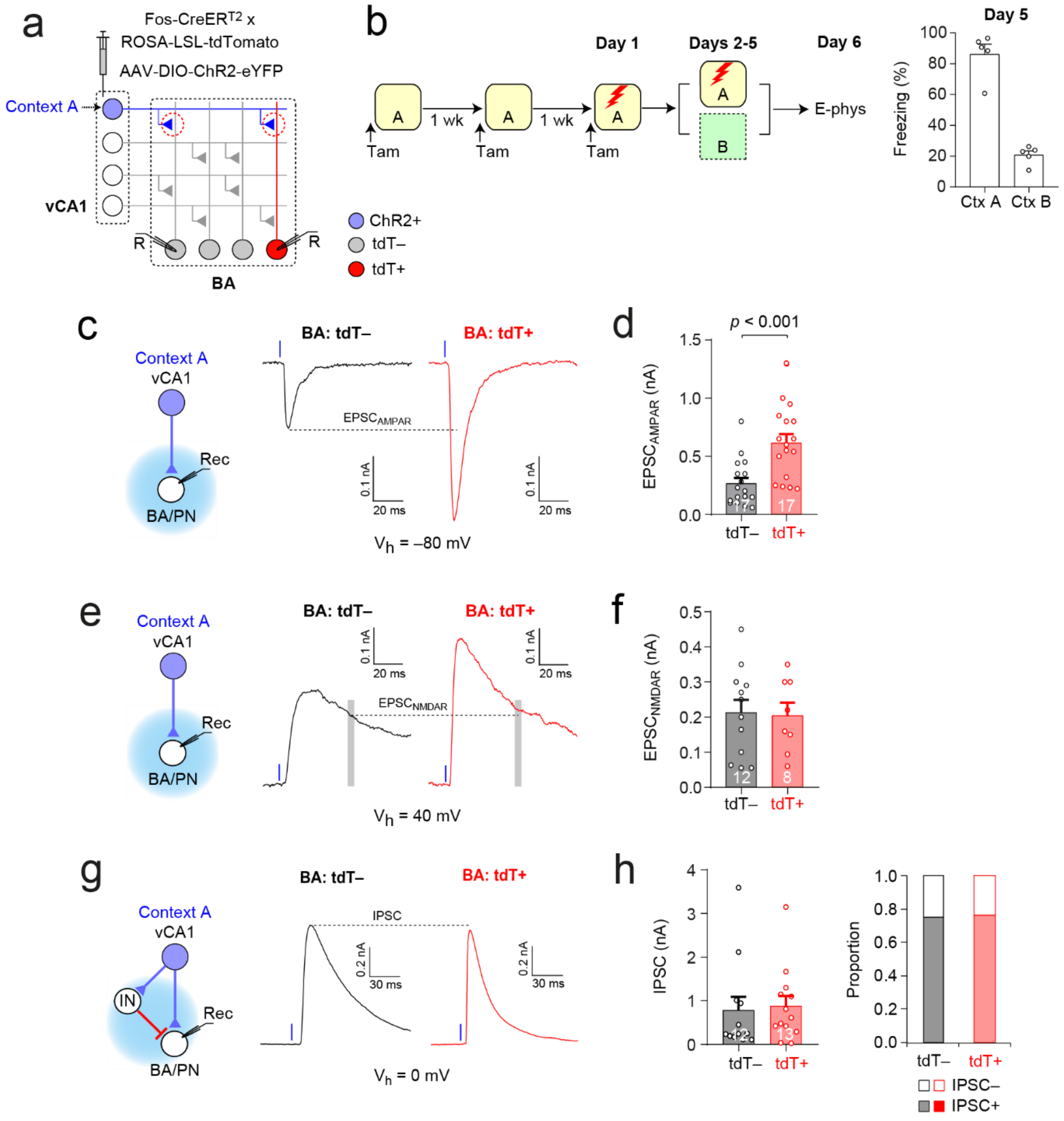

Supplementary Figure 12. Comparison of excitatory and inhibitory synaptic responses in Context A vCA1 inputs to BA fear neurons versus other BA neurons

(a) Experimental setup for (b)-(h). AAV-DIO-ChR2 was injected into the vCA1 in Fos-CreER^{T2} x ROSA-LSL-tdTomato mice.

(b) ***Left***: mice were exposed to Context A for ChR2 expression in vCA1 neurons active in Context A. On Day 1, the mice were fear conditioned in Context A for tdTomato (tdT) expression in BA fear neurons. On Days 2-5, mice were trained for discriminative fear in Context A as in **Fig. 5b**. ***Right***: quantification of freezing behavior in Contexts (Ctx) A and B on Day 5. n = 5 mice.

(c) Traces of AMPA receptor-mediated EPSCs induced by photostimulation of Context A vCA1 inputs (20.0 mW/mm^2; 1 ms duration; blue vertical bars) and recorded in tdT– and tdT+ BA neurons at holding potentials (V_h) of −80 mV. EPSCs were recorded in the presence of SR-95531.

(d) Comparison of the average amplitude of AMPAR EPSCs recorded in tdT– (17 cells) and tdT+ neurons (17 cells, two-sided unpaired ***t***-test) as in (c).

(e) Traces of NMDA receptor-mediated EPSCs induced by photostimulation of Context A vCA1 inputs (20.0 mW/mm^2; 1 ms duration) and recorded in tdT– and tdT+ BA neurons at V_h = +40 mV. EPSCs were recorded in the presence of SR-95531.

(f) Plot of the average amplitude of NMDAR EPSCs recorded in tdT– neurons (12 cells) and tdT+ neurons (8 cells) as in (e). ***p*** = 0.79, two-sided unpaired ***t***-test.

(g) Traces of inhibitory postsynaptic currents (IPSC) in the vCA1–BA feed-forward inhibitory circuit. IPSCs were evoked with photostimulation of Context A vCA1 inputs (20.0 mW/mm^2; 1 ms duration) and recorded in tdT+ and tdT– BA neurons at V_h = 0 mV. IPSCs were recorded in the absence of SR-95531.

(h) ***Left***: comparison of the average amplitude of IPSCs recorded in tdT– (12 cells) versus tdT+ BA neurons (13 cells) (***p*** = 0.81, two-sided unpaired ***t***-test). ***Right***: the proportion of BA neurons with feed-forward IPSC (***p*** = 0.92, Chi-squared test).

Error bars represent the SEM. Source data are provided as a Source Data file.

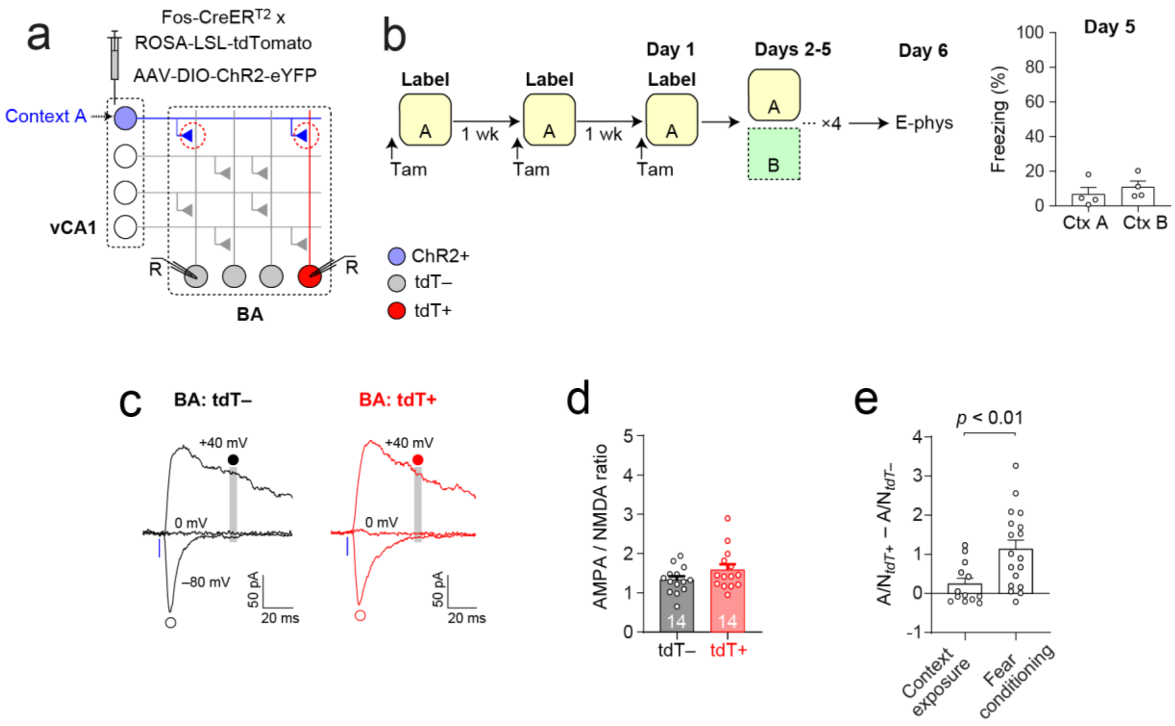

Supplementary Figure 13. The vCA1–BA pathway was not strengthened after context exposure without an aversive stimulus.

(a) Experimental setup for (b)-(d). AAV-DIO-ChR2 was injected into the vCA1 in Fos-CreER^{T2} x ROSA-LSL-tdTomato mice.

(b) ***Left***: after tamoxifen injection, mice were exposed to Context A for 12 minutes without the unconditioned stimulus (US, shock) three times at 1-week intervals to induce ChR2 and tdT expression in vCA1 and BA neurons active in Context A, respectively. On Days 2-5, mice were exposed to Contexts (Ctx) A and B without the US. ***Right***: quantification of freezing responses on Day 5. n = 4 mice.

(c) Traces of EPSCs recorded in tdT– and tdT+ BA neurons. EPSCs were induced and recorded as in **Fig. 4d**.

(d) Comparison of the AMPA/NMDA ratio between tdT– and tdT+ BA neurons (***p*** = 0.14, two-sided unpaired ***t***-test). n = 14 neurons per group.

(e) Quantification of difference in the AMPA/NMDA ratio between tdT+ and tdT– neurons in mice with context exposure alone as in (b) (13 pairs) versus mice fear conditioned in Context A in **Fig. 5a-g** (18 pairs) (two-sided unpaired ***t***-test).

Error bars represent the SEM. Source data are provided as a Source Data file.

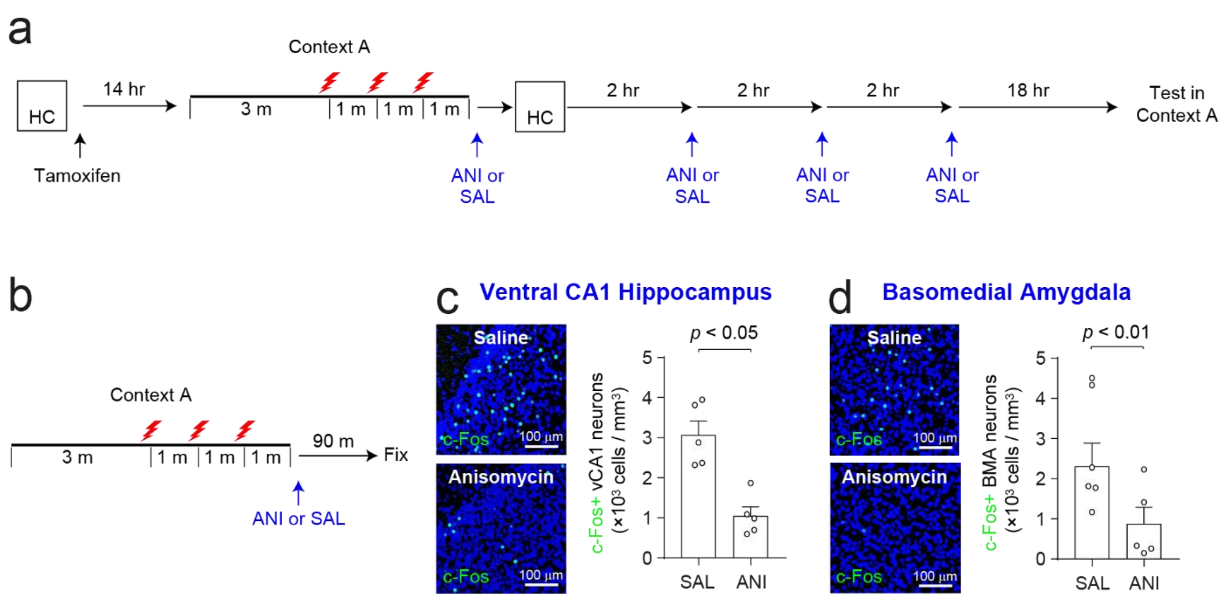

Supplementary Figure 14. Inhibition of protein synthesis by anisomycin

(a) Diagram showing a behavioral protocol used in **Fig. 6a-g**. Mice received three footshocks (0.5 mA, 2 s duration) in Context A 14 hours after tamoxifen administration. Immediately after fear conditioning, mice received an intraperitoneal injection of anisomycin (150 mg/kg body weight, ANI) or saline (SAL) and returned to their home cages. Mice received 3 more injections of anisomycin (50 mg/kg body weight) or saline at 2-hour intervals. Eighteen hours after the last injection, mice were tested for freezing behavior in Context A.

(b) Diagram showing a behavioral protocol for (c)-(d). Mice received three footshocks in Context A. Immediately after fear conditioning, mice received an intraperitoneal injection of anisomycin (150 mg/kg body weight, ANI) or saline (SAL) and returned to their home cages. Ninety minutes after fear conditioning, the brain tissue was fixed for c-Fos immunohistochemistry.

(c) ***Left***: representative images showing vCA1 neurons immunostained with c-Fos (green, circles). Blue, DAPI stain. ***Right***: comparison of the density of c-Fos+ cells in the vCA1 between the ANI and SAL groups. Two-sided unpaired ***t***-test. n = 5 mice per group.

(d) ***Left***: representative images showing BMA neurons immunostained with c-Fos (green, circles). Blue, DAPI stain. ***Right***: comparison of the density of c-Fos+ cells in the BMA between the SAL (6 mice) and ANI groups (5 mice). Two-sided unpaired ***t***-test.

Error bars represent the SEM. Source data are provided as a Source Data file.

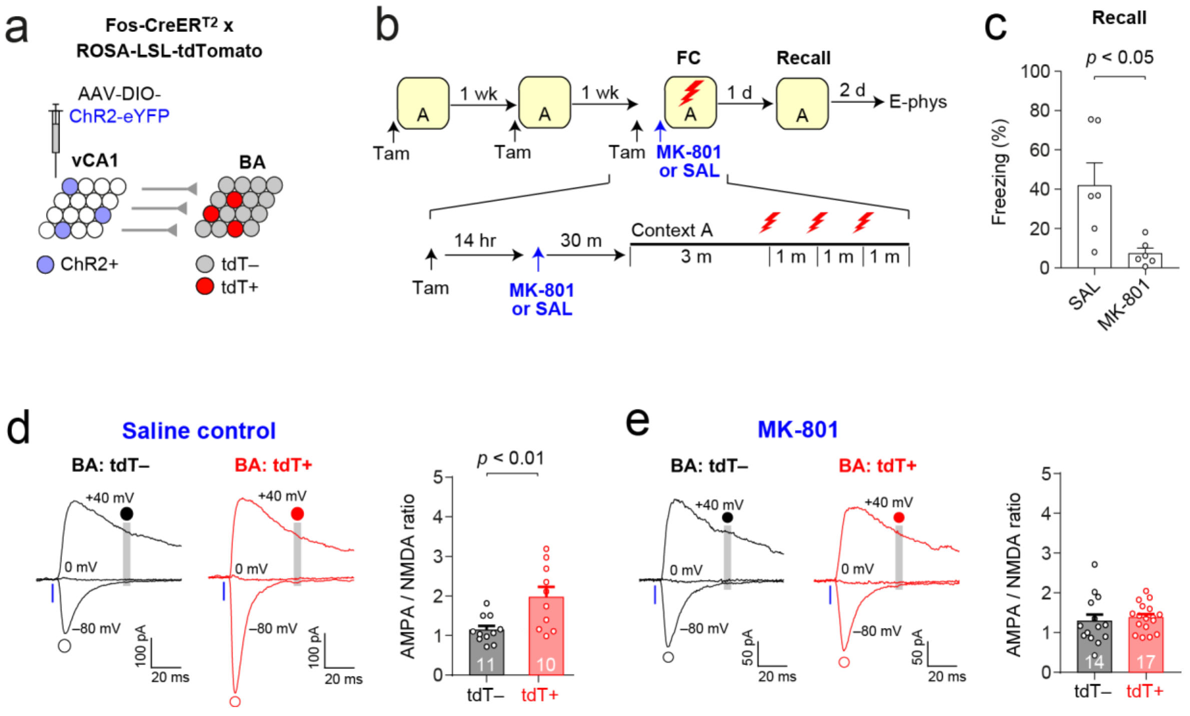

Supplementary Figure 15. Pretraining MK-801 treatment inhibits contextual fear learning and synaptic potentiation in the vCA1–BA pathway.

(a) Experimental setup for (b)-(e). AAV-DIO-ChR2-eYFP was injected into the vCA1 in Fos-CreER^{T2} x ROSA-LSL-tdTomato mice.

(b) Mice were given tamoxifen (Tam) injection and exposed to Context A twice with a 1-week interval to induce ChR2 expression in vCA1 neurons active in Context A. After a week, the mice received the third tamoxifen injection. Fourteen hours after tamoxifen administration, the mice received an intraperitoneal injection of MK-801 or saline (SAL) and fear conditioned in Context A 30 minutes later, which induced tdTomato (tdT) expression in BA neurons active during contextual fear conditioning.

(c) Comparison of freezing behavior in Context A 24 hours after fear conditioning. Two-sided unpaired ***t***-test. n = 6 mice per group.

(d) ***Left***: traces of EPSCs recorded in tdT– and tdT+ BA neurons in the saline control group (SAL). EPSCs were induced by photostimulation of Context A vCA1 inputs and recorded as in tdT– and tdT+ BA neurons. ***Right***: comparison of the AMPA/NMDA ratio in tdT– (11 neurons) and tdT+ neurons (10 neurons) in the saline control group ($p < 0.01$). Two-way ANOVA with ***post hoc*** comparisons was used to analyze combined data in (d) and (e).

(e) ***Left***: traces of EPSCs recorded in tdT– and tdT+ BA neurons in the MK-801 group. ***Right***: comparison of the AMPA/NMDA ratio in tdT– (14 neurons) and tdT+ neurons (17 neurons) in the MK-801 group ($p = 1.00$, Two-way ANOVA with ***post hoc*** comparisons).

Error bars represent the SEM. Source data are provided as a Source Data file.

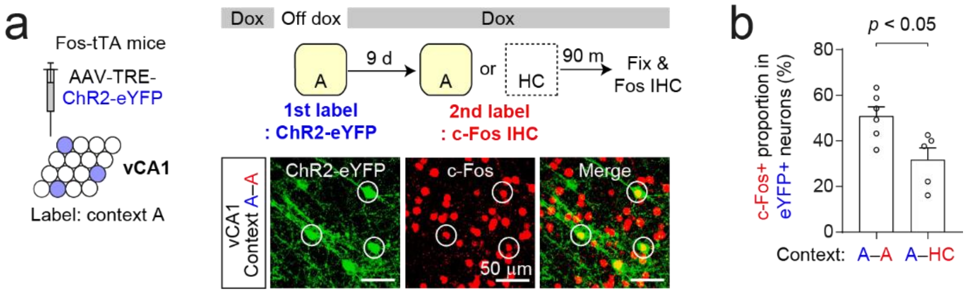

Supplementary Figure 16. Context-specific labeling of vCA1 neurons in Fos-tTA mice.

(a) ***Left***: experimental setup. AAV-TRE-ChR2-eYFP was injected into the vCA1 in Fos-tTA mice fed with Dox. ***Right top***: mice were taken off Dox for 48 hours and exposed to Context A. After 9 days, mice in the A–A group (6 mice) were exposed to the Context A, whereas mice in the A–HC group (5 mice) remained in the home cages. The brain tissue was fixed 90 minutes later for c-Fos immunohistochemistry (IHC). ***Right bottom***: images showing ChR2-eYFP+ (green) and c-Fos+ (red) vCA1 neurons. Some vCA1 neurons labeled with both ChR2-eYFP and c-Fos are circled.

(b) Comparison of c-Fos+ proportion among all ChR2-eYFP+ vCA1 neurons (two-sided unpaired ***t***-test).

Error bars represent the SEM. Source data are provided as a Source Data file.

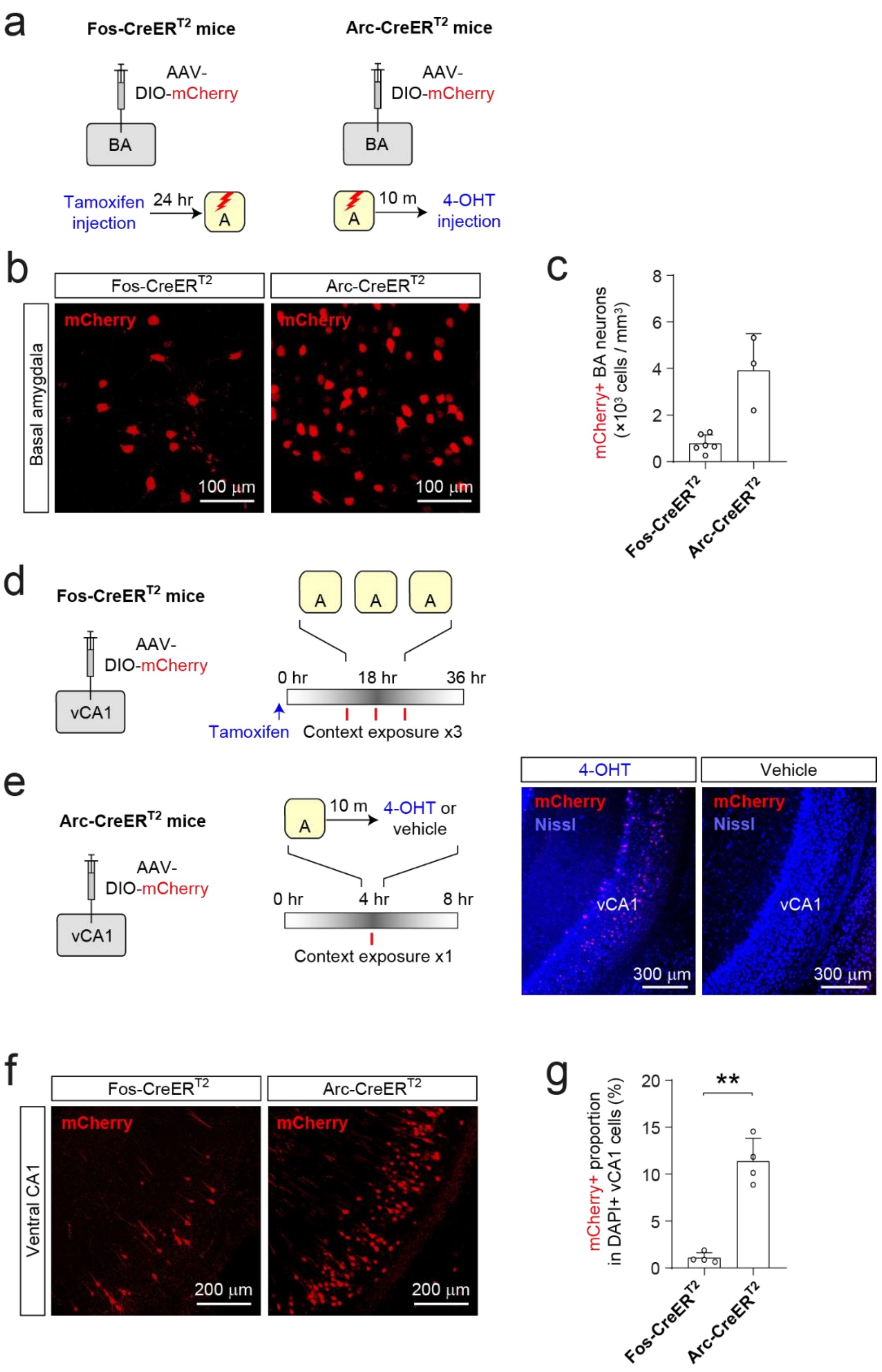

Supplementary Figure 17. Comparison of neural activity-dependent labeling of BA and vCA1 neurons in Fos-CreER^{T2} versus Arc-CreER^{T2} mice.

(a) Experimental setup for (b)-(c). AAV-DIO-mCherry was injected into the BA in Fos-CreER^{T2} mice (left) and Arc-CreER^{T2} mice (right). BA neurons active during fear conditioning in Context A were labeled with mCherry.

(b) Z-stacked confocal microscopic images showing mCherry expression in a subset of BA neurons in Fos-CreER^{T2} (left) and Arc-CreER^{T2} mice (right).

(c) Comparison of mCherry+ BA neuron density in Fos-CreER^{T2} versus Arc-CreER^{T2} mice. $\boldsymbol{p} = 0.07$ (two-sided unpaired ***t***-test.

(d) Experimental setup for (f)-(g). AAV-DIO-mCherry was injected into the vCA1 in Fos-CreER^{T2} mice. After an intraperitoneal injection of tamoxifen, mice were exposed to novel Context A three times for 12 minutes each to label vCA1 neurons active in Context A as in **Fig. 3b**.

(e) ***Left***: experimental setup for (f)-(g). AAV-DIO-mCherry was injected into the vCA1 in Arc-CreER^{T2} mice. After surgery, mice were exposed to novel Context A once for 12 minutes and received an intraperitoneal injection of 4-hydroxytamoxifen (4-OHT) or vehicle 10 minutes later. ***Right***: microscopic images showing mCherry expression (red) in a population of vCA1 neurons in mice that received 4-OHT injection after context exposure but not in mice that received vehicle injection. Blue, Nissl stain.

(f) Z-stacked confocal microscopic images showing mCherry expression in a subset of vCA1 neurons in Fos-CreER^{T2} (left) and Arc-CreER^{T2} mice (right). AAV-DIO-mCherry was injected into the vCA1. vCA1 neurons active in Context A were labeled with mCherry as in (d)-(e).

(g) Comparison of mCherry+ proportion among all DAPI+ vCA1 cells in Fos-CreER^{T2} versus Arc-CreER^{T2} mice. ** $\boldsymbol{p} < 0.01$, two-sided unpaired ***t***-test.

Error bars represent the SEM. Source data are provided as a Source Data file.

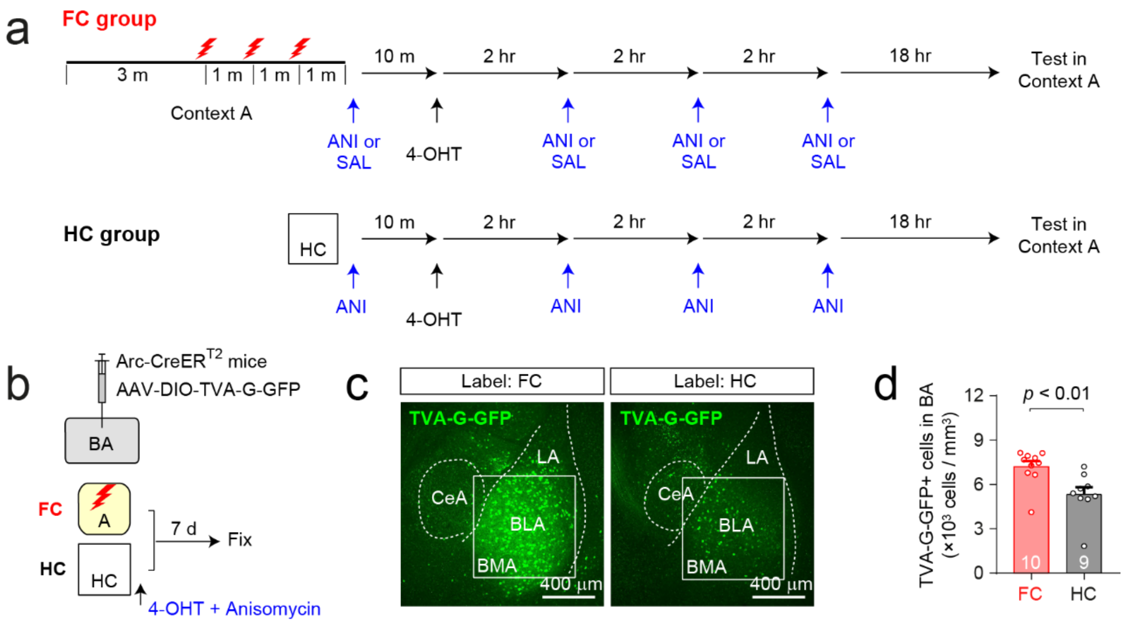

Supplementary Figure 18. Labeling of BA neurons active during fear conditioning and those active in the home cages.

(a) Diagram showing a behavioral protocol used in **Fig. 8a-g**. Immediately after fear conditioning in Context A, mice in the FC group received an intraperitoneal injection of anisomycin (150 mg/kg body weight, ANI) or saline (SAL) and returned to their home cages. The mice also received an intraperitoneal injection of 4-OHT (15 mg/kg body weight) 10 minutes after fear conditioning. Mice then received 3 more injections of anisomycin (50 mg/kg body weight) or saline at 2-hour intervals. Eighteen hours after the last injection, mice were tested for freezing behavior in Context A. Mice in the home cage (HC) group remained in their home cages and received anisomycin and 4-OHT as in the FC group.

(b) Experimental setup for (c)-(d). AAV-DIO-TVA-G-GFP was injected into the BA in Arc-CreER^{T2} mice as in **Fig. 8a-g**. After surgery, mice in the FC and HC groups were trained and received injections of anisomycin and 4-OHT as in (a). TVA-G-GFP was expressed in BA neurons active during contextual fear conditioning in the FC group (10 mice), whereas it was expressed in BA neurons active in the home cages in the HC group (9 mice) under the control of the Arc promoter in the presence of 4-OHT.

(c) Representative images showing TVA-G-GFP-labeled neurons in the BA. Squares indicate the BA areas, in which labeled BA neurons were counted to calculate the density of labeled neurons.

(d) Comparison of the density of TVA-G-GFP-labeled BA neurons between the FC and HC groups. Two-sided unpaired ***t***-test.

Error bars represent the SEM. Source data are provided as a Source Data file.

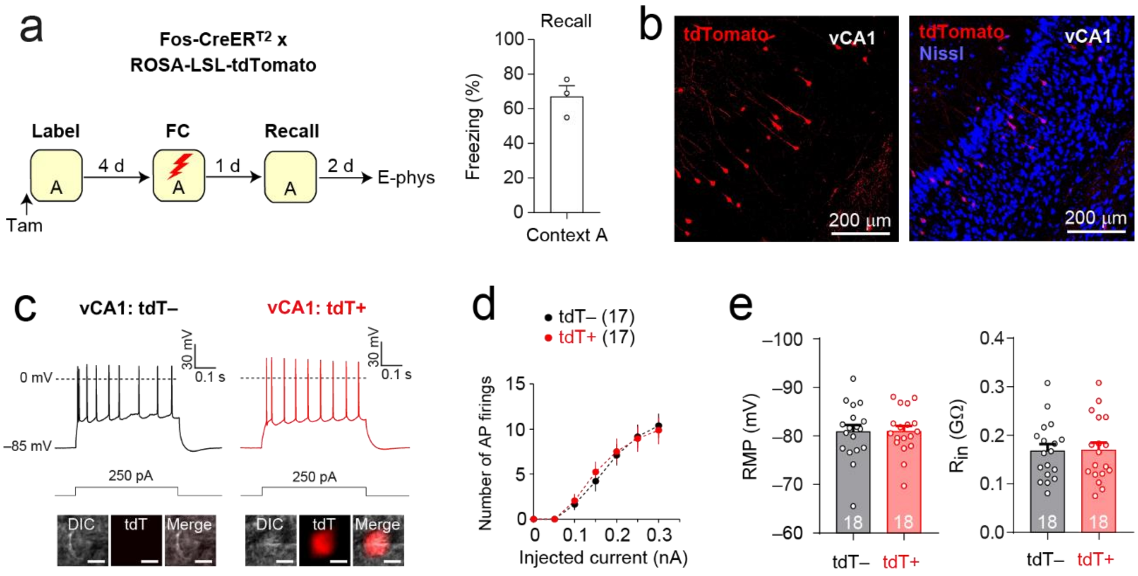

Supplementary Figure 19. Contextual fear conditioning did not affect neuronal excitability or intrinsic membrane properties of context-specific vCA1 neurons.

(a) ***Left***: experimental setup. After tamoxifen injection (Tam), Fos-CreER^{T2} × ROSA-LSL-tdTomato mice were exposed to Context A to label with tdTomato (tdT) vCA1 neurons active in Context A. Four days after vCA1 labeling, the mice were fear conditioned in Context A. The mice were tested for freezing behavior in Context A 24 hours later (Recall). Electrophysiological recordings (E-phys) were performed 2 days after memory recall test. ***Right***: quantification of freezing behavior in Context A 24 hours after contextual fear conditioning. n = 3 mice.

(b) Microscopic images showing tdT-labeled vCA1 neurons (red). Blue, Nissl stain.

(c) Representative traces of action potential (AP) firing induced by depolarizing current injection (500 ms long) and recorded in tdT– and tdT+ vCA1 neurons in current-clamp mode. Baseline membrane potential was adjusted to approximate –85 mV. tdT+ neurons were identified with red fluorescence (inset; scale bar, 10 μm).

(d) Summary plot of AP firing in tdT– and tdT+ vCA1 neurons. The average number of APs was plotted against injected currents. There was no significant difference in AP firings between tdT– and tdT+ vCA1 neurons (***p*** = 0.67; repeated measures two-way ANOVA). n = 17 cells per group from 3 mice.

(e) Comparison of resting membrane potential (RMP, left) and input resistance (R_{in}, right) in tdT– and tdT+ vCA1 neurons. Input resistance was calculated as in **Supplementary Figure 8**. There was no significant difference in RMP (***p*** = 0.94, two-sided unpaired ***t***-test) or R_{in} between tdT– and tdT+ vCA1 neurons (***p*** = 0.91, two-sided unpaired ***t***-test). n = 18 cells per group from 3 mice.

Error bars represent the SEM. Source data are provided as a Source Data file.

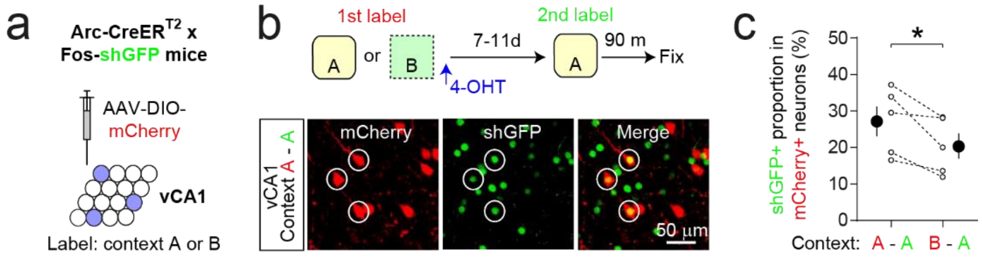

Supplementary Figure 20. Context-specific labeling of vCA1 neurons in Arc-CreER^{T2} mice.

(a) Experimental setup for (b)-(c). AAV-DIO-mCherry was injected into the vCA1 in Arc-CreER^{T2} × Fos-shGFP mice, which express CreER^{T2} and short half-life GFP (shGFP, half-life 2 hours) under the control of the neural activity-dependent Arc and Fos promoters, respectively.

(b) ***Top***: mice were exposed to Context A or B and injected with 4-OHT. After 7-11 days, mice were exposed to Context A, and the brain tissue was fixed 90 minutes later. ***Bottom***: images showing vCA1 neurons labeled with mCherry (red) and shGFP (green). vCA1 neurons active during both the first and second context exposures expressed both mCherry and shGFP (circled).

(c) Comparison of the proportion of shGFP+ cells among all mCherry+ vCA1 neurons (* $p < 0.05$, two-sided paired t-test, $n = 5$ pairs of mice). Open circles indicate the proportion in each mouse, and closed circles are the average proportion.

Error bars represent the SEM. Source data are provided as a Source Data file.

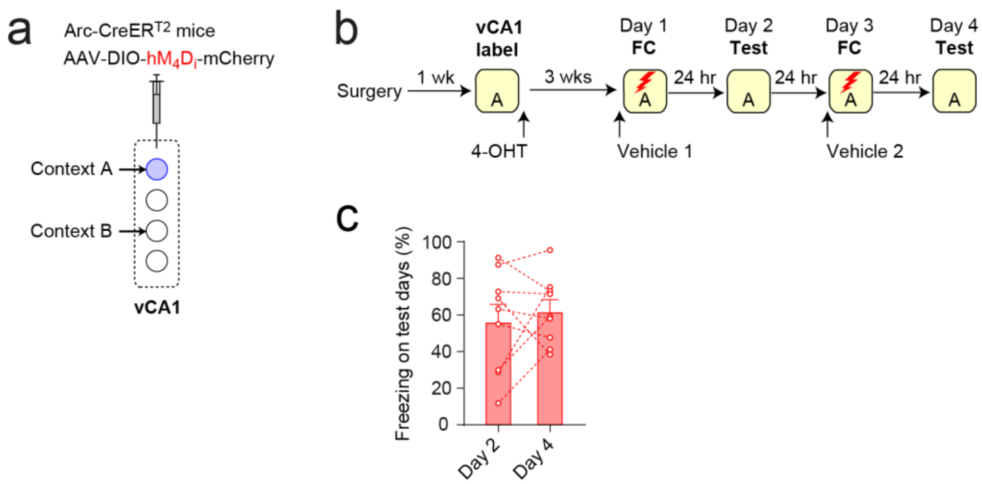

Supplementary Figure 21. Vehicle injection did not affect contextual fear learning in mice with hM$_4$D$_i$ expression in Context A vCA1 neurons.

(a) vCA1 neurons active in Context A expressed hM$_4$D$_i$-mCherry.

(b) Behavioral training and testing protocols for (c). Three weeks after labeling vCA1 neurons active in Context A with hM$_4$D$_i$-mCherry, mice received a vehicle injection 30 minutes before fear conditioning in Context A on Day 1 (vehicle 1) and Day 3 (vehicle 2). The mice were tested for fear memory in Context A on Days 2 and 4.

(c) Comparison of freezing behavior in Context A on Day 2 versus Day 4. There was no significant difference in freezing behavior on Day 2 versus Day 4 (***p*** = 0.52, two-sided paired ***t***-test; 9 mice), suggesting that the CNO effect in the hM$_4$D$_i$ group in **Fig. 9a-c** was not due to the order of CNO and vehicle injections before fear conditioning.

Error bars indicate the SEM. Source data are provided as a Source Data file.

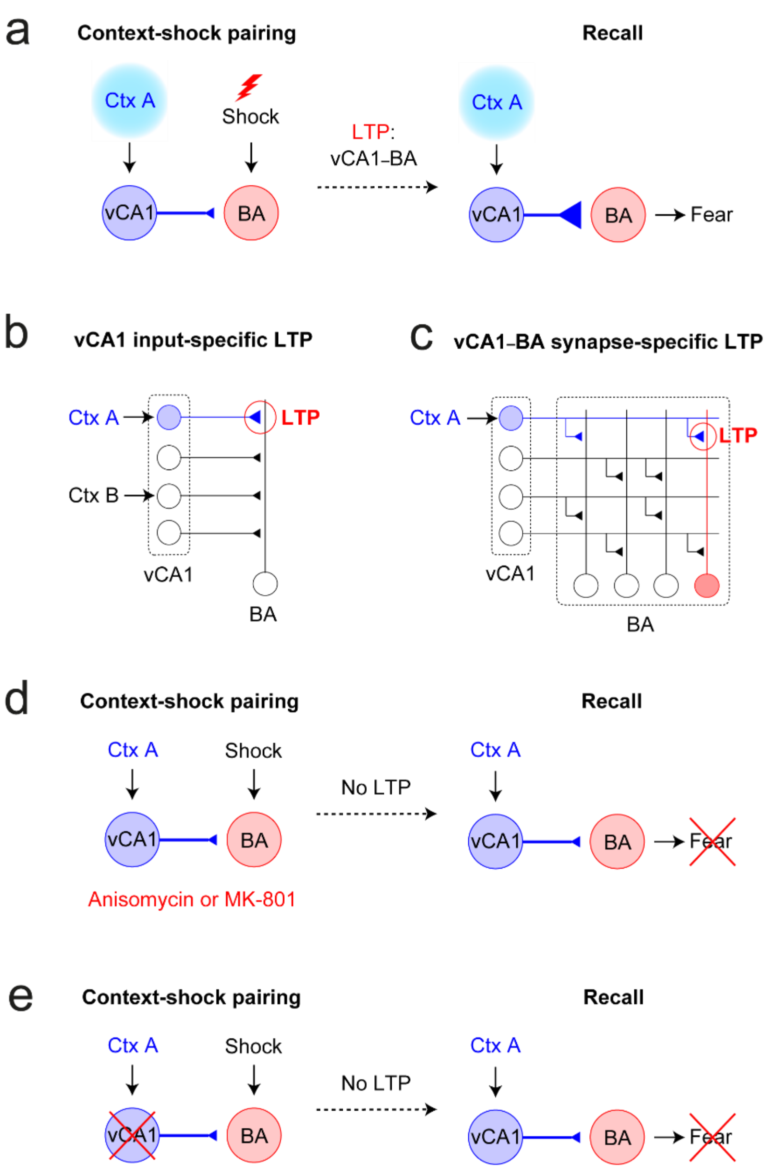

Supplementary Figure 22. Summary diagrams

(a) The context-shock pairing during contextual fear conditioning induces long-term synaptic potentiation (LTP) in the vCA1–BA pathway. A subset of vCA1 neurons is active in a specific context (e.g., Context A or Ctx A), whereas a neuronal population in the BA responds to the aversive stimulus (e.g., shock). During memory recall, vCA1 neurons responding to the threat-predictive context activate BA neurons more readily through the strengthened vCA1–BA pathway, inducing fear responses to the context.

(b) As demonstrated in **Fig. 4**, contextual fear conditioning induces selective strengthening in vCA1 inputs that convey threat-predictive context (Ctx A) signals to the BA (red open circle, LTP).

(c) As demonstrated in **Fig. 5, 7, and 10a-g**, contextual fear conditioning selectively strengthens synapses (red open circle, LTP) that consist of presynaptic vCA1 neurons active in the threat-predictive context (Ctx A, blue filled circle) and postsynaptic BA fear neurons active during fear conditioning (red filled circle).

(d) Synaptic potentiation in the vCA1–BA pathway is blocked under anisomycin-induced retrograde amnesia or MK-801-induced anterograde amnesia as demonstrated in **Fig. 6a-g and Supplementary Figure 15**.

(e) Silencing of context-specific vCA1 neurons during fear conditioning inhibits both synaptic potentiation in the vCA1–BA pathway and fear memory formation as shown in **Fig. 10h-n**.

Supplementary Table 1. Summary of Statistical Analysis: ANOVA

Data	ANOVA	Response variable	Factors	DF*	F value	P value
**Figure 1d**	One-way	c-Fos+ proportion	Behavioral groups	(2,15)	15.19	< 0.001
			HC group (n = 6 mice)			
			Context group (n = 6 mice)			
			FC group (n = 6 mice)			
			** Post hoc comparisons			
			HC versus Context group: P = 0.001			
			HC versus FC group: P < 0.001			
			Context versus FC group: P = 0.861			
**Figure 1h**	Repeated measures two-way	Freezing time (%)	Behavioral groups	(1,16)	3.71	0.072
			hM4Di (n = 10 mice)			
			mCherry (n = 8 mice)			
			Treatment	(1,16)	9.10	0.008
			CNO			
			Vehicle			
			Interaction	(1,16)	6.01	0.026
			Subject	(16,16)	4.13	0.004
			** Post hoc comparisons			
			hM4Di-CNO versus hM4Di-vehicle: P = 0.005			
			hM4Di-CNO versus mCherry-CNO: P = 0.042			
**Figure 2e**	Repeated measures two-way	ON - OFF freezing (%)	Behavioral groups	(1,11)	5.68	0.036
			Chronos (n = 7 mice)			
			eYFP (n = 6 mice)			
			Behavioral session	(1,11)	4.15	0.066
			Habituation (HB)			
			Test			
			Interaction	(1,11)	10.89	0.007
			Subject	(11,11)	2.41	0.080
			** Post hoc comparisons			
			Chronos-HB versus Chronos-test: P = 0.014			
			Chronos-test versus eYFP-test: P = 0.012			
**Figure 5f, 5n**	Two-way	AMPA/NMDA ratio	vCA1 inputs	(1,52)	10.87	0.002
			Context A-specific (Fig. 5f)			
			Nonspecific (Fig. 5n)			
			BA cell type	(1,52)	8.80	0.005
			tdTomato- cells tdTomato + cells			
			Interaction	(1,52)	10.88	0.002
			** Post hoc comparisons			
			Context A inputs/tdT+ cells versus Context A inputs/tdT- cells: P < 0.001			
			Context A inputs/tdT+ cells versus Nonspecific inputs/tdT+ cells: P < 0.001			
			Nonspecific inputs/tdT+ cells versus Nonspecific inputs/tdT- cells: P = 1.000			
**Figure 5i**	Repeated measures two-way	AP firing number	BA cell type	(1,165)	0.18	0.671
			tdTomato- (n = 18 cells)			
			tdTomato+ (n = 17 cells)			
			Current injection	(5,165)	34.65	< 0.001
			50, 100, 150, 200, 250, and 300 pA			
			Interaction	(5,165)	0.39	0.858
			Cell	(33,165)	8.27	< 0.001
**Figure 6e-f**	Two-way	AMPA/NMDA ratio	Treatment	(1,80)	6.03	0.016
			Saline control (SAL, Fig. 6e)			
			Anisomycin (ANI, Fig. 6f)			
			BA cell type	(1,80)	13.63	< 0.001
			tdTomato- cells tdTomato + cells			
			Interaction	(1,80)	7.93	0.006

Data	ANOVA	Response variable	Factors	DF*	F value	P value
			** Post hoc comparisons<br>SAL/tdT- versus SAL/tdT+: P < 0.001 SAL/tdT + versus ANI/tdT+: P = 0.002 ANI/tdT- versus ANI/tdT+: P = 1.000			
Figure 8b	One-way	Freezing time (%)	Behavioral groups<br>FC/SAL group (n = 12 mice) FC/ ANI group (n = 10 mice) HC/ANI group (n = 9 mice)	(2,29)	17.36	< 0.001
			** Post hoc comparisons<br>FC/SAL versus FC/ANI group: P < 0.001 FC/ SAL versus HC/ANI group: P < 0.001 FC/ANI versus HC/ANI group: P = 0.891			
Figure 8l	Repeated measures two-way	AMPAR EPSC	BA cell type<br>tdTomato- (n = 16 cells)<br>tdTomato+ (n = 16 cells)	(1,90)	80.32	< 0.001
			Photostimulation intensity<br>2.8, 6.3, 13.4, and 20.5 mW/mm2	(3,90)	111.38	< 0.001
			Interaction	(3,90)	0.85	0.469
			Cell	(30,90)	23.85	< 0.001
Figure 9c	Repeated measures two-way	Freezing time (%)	Behavioral groups<br>hM4Di (n = 12 mice)<br>mCherry (n = 8 mice)	(1,18)	5.98	0.025
			Treatment<br>CNO<br>Vehicle	(1,18)	7.46	0.014
			Interaction	(1,18)	6.21	0.023
			Subject	(18,18)	2.26	0.046
			** Post hoc comparisons<br>hM4Di-CNO versus hM4Di-vehicle: P = 0.004 hM4Di-CNO versus mCherry-CNO: P = 0.016 mCherry-CNO versus mCherry-vehicle: P = 1.000			
Figure 10g, 10n	Two-way	AMPA/NMDA ratio	Treatment<br>No CNO control (Fig. 10g)<br>CNO (Fig. 10n)	(1,40)	2.95	0.094
			BA cell type<br>mCherry- cells mCherry + cells	(1,40)	5.60	0.023
			Interaction	(1,40)	9.88	0.003
			** Post hoc comparisons<br>No CNO/mCherry- versus No CNO/mCherry+: P = 0.002<br>CNO/mCherry- versus CNO/mCherry+: P = 1.000<br>No CNO/mCherry+ versus CNO/mCherry+: P = 0.007			
Supplementary Figure 2c	Repeated measures two-way	AP firing number	CNO treatment (n = 12 cells)<br>Pre-CNO<br>Post-CNO	(1,132)	37.24	< 0.001
			Current injection<br>50, 100, 150, 200, 250, 300, and 350 pA	(6,132)	20.68	< 0.001
			Interaction	(6,132)	2.05	0.063
			Cell	(22,132)	8.38	< 0.001
Supplementary Figure 5d	One-way	tdTomato+ cells	Behavioral groups<br>A-HC/7d (n = 5 mice)<br>A-A/7d (n = 5 mice) A-A/21d (n = 6 mice)<br>A-A/label 3x (n = 6 mice)	(3,18)	5.79	0.006

Data	ANOVA	Response variable	Factors	DF*	F value	P value
			** Post hoc comparisons			
			A-HC/7d versus A-A/label 3x: P = 0.010			
			A-A/7d versus A-A/label 3x: P = 0.032 A-			
			A/21d versus A-A/label 3x: P = 0.042			
**Supplementary Figure 5e**	One-way	c-Fos+ proportion	Behavioral groups	(3,17)	9.74	0.001
			A-HC/7d (n = 5 mice)			
			A-A/7d (n = 5 mice) A-			
			A/21d (n = 6 mice)			
			A-A/label 3x (n = 5 mice)			
			** Post hoc comparisons			
			A-HC/7d versus A-A/7d: P = 0.001 A-			
			HC/7d versus A-A/21d: P = 0.032			
			A-HC/7d versus A-A/label 3x: P = 0.005			
**Supplementary Figure 9d**	Repeated measures two-way	Paired-pulse ratio	Behavioral groups	(1,33)	1.67	0.205
			FC group (n = 12 cells)			
			NS group (n = 13 cells)			
			Photostimulation intensity	(2,33)	2.53	0.095
			5.4, 12.5, and 26.4 mW/mm^2			
			Interaction	(2,33)	0.21	0.812
			Cell	(21,33)	5.25	< 0.001
**Supplementary Figure 11e**	Repeated measures two-way	Paired-pulse ratio	Behavioral groups	(1,49)	2.76	0.103
			FC group (n = 14 cells)			
			NS group (n = 14 cells)			
			Photostimulation intensity	(2,49)	12.58	< 0.001
			6.3, 13.4, and 20.5 mW/mm^2			
			Interaction	(2,49)	0.40	0.669
			Cell	(26,49)	21.22	< 0.001
**Supplementary Figure 15d-e**	Two-way	AMPA/NMDA ratio	Treatment	(1,48)	2.10	0.154
			Saline			
			MK-801			
			BA cell type	(1,48)	9.03	0.004
			tdT- cells			
			tdT+ cells			
			Interaction	(1,48)	5.97	0.018
			** Post hoc comparisons			
			Saline/tdT- versus saline/tdT+: P = 0.005 MK-801/			
			tdT- versus MK-801/tdT+: P = 1.000 Saline/tdT+			
			versus MK-801/tdT+: P = 0.048			
**Supplementary Figure 19d**	Repeated measures two-way	AP firing number	BA cell type	(1,160)	0.18	0.673
			tdTomato- (n = 17 cells)			
			tdTomato+ (n = 17 cells)			
			Current injection	(5,160)	78.86	< 0.001
			50, 100, 150, 200, 250, and 300 pA			
			Interaction	(5,160)	0.39	0.853
			Cell	(32,160)	12.78	< 0.001

* DF: degree of freedom
** Post hoc Bonferroni's simultaneous multiple comparisons

Supplementary Table 2. Summary of Statistical Analysis: t-test*

Data	t-test	Response variable	Groups	DF**	t value	P value
**Figure 2d**	Paired	Freezing time (Chronos: paired, HB)	Laser on vs laser off (n = 7 mice)	6	-0.62	0.555
**Figure 2g**	Paired	ON - OFF freezing (%)	HB vs test (n = 9 mice)	8	-0.91	0.390
**Figure 3f**	Paired	tdTomato+ cells	Context A-A vs Context A-B (n = 6 pairs of mice)	5	0.42	0.689
**Figure 3f**	Paired	c-Fos+ cells	Context A-A vs Context A-B (n = 6 pairs of mice)	5	0.68	0.528
**Figure 3f**	Paired	Fos+ and tdT+ cells	Context A-A vs Context A-B (n = 6 pairs of mice)	5	3.65	0.015
**Figure 3f**	Paired	Fos+ proportion among tdT+ cells	Context A-A vs Context A-B (n = 6 pairs of mice)	5	4.47	0.007
**Figure 5c**	Paired	Freezing time (%)	Context A vs Context B (n = 5 mice)	4	9.59	0.001
**Figure 5g**	Paired	AMPAR EPSC	tdTomato- vs tdTomato+ cells (n = 18 pairs of neurons)	17	-3.07	0.007
**Figure 5j**	Unpaired	RMP	tdTomato- (n = 18 cells) tdTomato+ (n = 17 cells)	32	0.77	0.447
**Figure 5j**	Unpaired	Input resistance	tdTomato- (n = 17 cells) tdTomato+ (n = 18 cells)	32	-0.05	0.957
**Figure 5l**	Paired	Freezing time (%)	Context A vs Context B (n = 5 mice)	4	9.51	0.001
**Figure 6c**	Unpaired	Freezing time (%)	Saline (n = 7 mice) Anisomycin (n = 10 mice)	9	4.47	0.002
**Figure 6j**	Unpaired	c-Fos+ proportion	FC (n = 5 mice) HC (n = 7 mice)	9	3.27	0.010
**Figure 6j**	Unpaired	c-Fos+ proportion	FC (n = 6 mice) HC (n = 6 mice)	9	2.29	0.048
**Figure 7f**	Paired	AMPA/NMDA ratio	mCherry- vs mCherry+ BA neurons (n = 12 pairs of neurons)	11	-3.41	0.006
**Figure 8g**	Unpaired	mCherry+ cells	FC (n = 10 mice) HC (n = 9 mice)	13	1.37	0.194
**Figure 8g**	Unpaired	c-Fos+ cells	FC (n = 10 mice) HC (n = 9 mice)	16	1.31	0.210
**Figure 8g**	Unpaired	c-Fos+ proportion	FC (n = 10 mice) HC (n = 9 mice)	16	3.96	0.001
**Figure 9f**	Paired	Freezing time (%)	CNO vs vehicle (7 mice)	6	-1.54	0.175
**Figure 10c, 10j**	Unpaired	Freezing time (%)	No CNO control (n = 6 mice) CNO (n = 5 mice)	6	7.14	< 0.001
**Supplementary Figure 2e**	Paired	Freezing time (%)	Day 2 vd Day 4 (8 mice)	7	-0.71	0.501
**Supplementary Figure 4g**	Paired	tdT+ proportion among DAPI+ cells	Context A-A vs Context A-B (n = 6 pairs of mice)	5	0.42	0.689

Data	t-test	Response variable	Groups	DF**	t value	P value
**Supplementary Figure 4g**	Paired	Fos+ proportion among DAPI+ cells	Context A-A vs Context A-B (n = 6 pairs of mice)	5	0.68	0.528
**Supplementary Figure 5b**	Unpaired	tdT+ cells	HC (n = 6 mice) Ctx A (n = 11 cells)	14	-4.05	0.001
**Supplementary Figure 9b**	Unpaired	NMDAR EPSC decay constant	FC (n = 12 cells) NS (n = 12 cells)	21	0.72	0.477
**Supplementary Figure 9c**	Unpaired	NMDAR EPSC decay constant	FC (n = 11 cells) NS (n = 12 cells)	19	-1.71	0.103
**Supplementary Figure 11d**	Unpaired	NMDAR EPSC decay constant	FC (n = 9 cells) NS (n = 10 cells)	16	-1.20	0.249
**Supplementary Figure 12d**	Unpaired	AMPAR EPSC	tdTomato- (n = 17 cells) tdTomato+ (n = 17 cells)	27	-3.89	< 0.001
**Supplementary Figure 12f**	Unpaired	NMDAR EPSC	tdTomato- (n = 12 cells) tdTomato+ (n = 8 cells)	17	0.16	0.878
**Supplementary Figure 12h**	Unpaired	IPSC	tdTomato- (n = 12 cells) tdTomato+ (n = 13 cells)	21	-0.24	0.809
**Supplementary Figure 13e**	Unpaired	AN (tdT+) - AN (tdT-)	No shock control (n = 13 pairs) Fear conditioning group (n = 18 pairs)	27	-3.27	0.003
**Supplementary Figure 14c**	Unpaired	c-Fos+ cells	Saline (SAL, 5 mice) Anisomycin (ANI, 5 mice)	4	3.75	0.020
**Supplementary Figure 14d**	Unpaired	c-Fos+ cells	Saline (SAL, 5 mice) Anisomycin (ANI, 5 mice)	4	5.52	0.005
**Supplementary Figure 15c**	Unpaired	Freezing time (%)	Saline (SAL, 6 mice) MK-801 (6 mice)	5	2.95	0.032
**Supplementary Figure 16b**	Unpaired	c-Fos proportion	A-A (n = 6 mice) A-HC (n = 5 mice)	8	2.86	0.021
**Supplementary Figure 17c**	Unpaired	mCherry+ BA neurons	Fos-Cre mice (6 mice) Arc-Cre mice (3 mice)	2	-3.39	0.077
**Supplementary Figure 17g**	Unpaired	mCherry+ proportion	Fos-Cre mice (4 mice) Arc-Cre mice (4 mice)	3	-8.17	0.004
**Supplementary Figure 18d**	Unpaired	TVA-G-GFP+ cells	FC (n = 10 mice) HC (n = 9 mice)	15	2.96	0.010
**Supplementary Figure 19e**	Paired	RMP	tdTomato- (n = 18 cells) tdTomato+ (n = 18 cells)	17	0.08	0.936
**Supplementary Figure 19e**	Paired	Input resistance	tdTomato- (n = 18 cells) tdTomato+ (n = 18 cells)	17	-0.11	0.912
**Supplementary Figure 20c**	Paired	c-Fos+ proportion	Context A-A vs Context B-A (n = 5 pairs of mice)	4	3.02	0.039
**Supplementary Figure 21c**	Paired	Freezing time (%)	Day 2 vd Day 4 (9 mice)	8	-0.67	0.523

* All t-tests were two-sided.
** DF: degree of freedom

Supplementary Table 3. Summary of Electrophysiological Data in Main Figures

Data	Mice	Labeling and training	ChR2+ vCA1 inputs	Recorded neurons	Results and interpretation
Fig. 4a-f	Fos-Cre	Label: Context A (x3, Tam)<br>* FC group: dCFC in Ctx A<br>* NS group: no shock in Ctx A	Context A inputs<br>(AAV-DIO-ChR2)	Randomly selected<br>BA neurons	AN (FC group) > AN (NS group)<br>* Strengthening in Context A vCA1 inputs to BA neurons
Fig. 4g-l	Fos-Cre	Label: Context B (x3, Tam)<br>* FC group: dCFC in Ctx A<br>* NS group: no shock in Ctx A	Context B inputs<br>(AAV-DIO-ChR2)	Randomly selected<br>BA neurons	No difference in AN (FC vs NS)<br>* No strengthening in Context B vCA1 inputs to BA neurons
Fig. 5a-g	Fos-Cre x LSL-tdT	Label 1: Context A (x3, Tam)<br>Label 2: CFC in Ctx A (Tam)	Context A inputs<br>(AAV-DIO-ChR2)	tdT+: BA fear neurons<br>tdT-: other BA neurons	AN (tdT+) > AN (tdT-)<br>AMPAR EPSC (tdT+) > AMPAR EPSC (tdT-)<br>* Strengthening in Context A vCA1 inputs to BA fear neurons
Fig. 5k-n	Fos-Cre x LSL-tdT	Label 1: Context A (x3, Tam)<br>Label 2: CFC in Ctx A (Tam)	Nonspecific inputs<br>(AAV-CaMKII-ChR2)	tdT+: BA fear neurons<br>tdT-: other BA neurons	No difference in AN (tdT+ vs tdT-)<br>* No strengthening in nonspecific vCA1 inputs to BA fear neurons
Fig. 6a-g	Fos-Cre x LSL-tdT	Label 1: Context A (x2, Tam)<br>Label 2: CFC in Ctx A (Tam, SAL or ANI)	AAV-DIO-ChR2<br>(Context A inputs)	tdT+: BA fear neurons<br>tdT-: other BA neurons	AN (tdT+) > AN (tdT-) in saline (SAL) control group<br>No difference in AN (tdT+ vs tdT-) in anisomycin (ANI) group<br>* Anisomycin prevents stregnthening of vCA1-BA pathway.
Fig. 7	Fos-Cre x Fos-tTA	Label 1: Context A (x3, Off Dox)<br><br>Label 2: CFC in Ctx A (Tam)	Context A inputs<br>(AAV-TRE-ChR2)	mCh+: BA fear neurons<br>mCh-: other BA neurons<br>(AAV-DIO-mCh)	AN (mCh+) > AN (mCh-)<br>* Strengthening in Context A vCA1 inputs to BA fear neurons
Fig. 10a-g	Arc-Cre x Fos-tTA	Label 1: Context A (4-OHT)<br><br>Label 2: CFC in Ctx A (Off Dox)	Context A inputs<br>(AAV-DIO-ChR2)	mCh+: BA fear neurons<br>mCh-: other BA neurons<br>(AAV-TRE-mCh)	AN (mCh+) > AN (mCh-)<br>* Strengthening in Context A vCA1 inputs to BA fear neurons
Fig. 10h-n	Arc-Cre x Fos-tTA	Label 1: Context A (4-OHT)<br><br>Label 2: CFC in Ctx A (Off Dox, + CNO)	Context A inputs<br>(AAV-DIO-ChR2 +<br>AAV-DIO-hM4Di)	mCh+: BA fear neurons<br>mCh-: other BA neurons<br>(AAV-TRE-mCh)	No difference in AN (mCh+ vs mCh-)<br>* Inhibition of Context A vCA1 activity prevents strengthening of vCA1-BA pathway.

* Major conclusions are highlighted in red.
** Abbreviations: Tam (Tamoxifen), FC (fear conditioning), dCFC (discriminative contextual fear conditioning), Ctx (Context), NS (no shock), AN (AMPA/NMDA EPSC ratio) CFC (contextual fear conditioning), tdT (tdTomato), SAL (saline), ANI (anisomycin), mCh (mCherry), 4-OHT (4-hydroxytamoxifen), CNO (clozapin N-oxide)

Supplementary Table 4. Summary of Electrophysiological Data in Supplementary Figures

Data	Mice	Labeling and training	ChR2+ vCA1 inputs	Recorded neurons	Results and interpretation
Suppl Fig. 9b,d	Fos-Cre	Label: Context A (x3, Tam)<br>* FC group: dCFC in Ctx A<br>* NS group: no shock in Ctx A	Context A inputs<br>(AAV-DIO-ChR2)	Randomly selected<br>BA neurons	No difference in NMDAR EPSC decay rate (FC vs NS)<br>No difference in PPR (FC vs NS)<br>* No presynaptically-expressed synaptic potentiation in Context A vCA1 inputs to BA neurons
Suppl Fig. 9c	Fos-Cre	Label: Context B (x3, Tam)<br>* FC group: dCFC in Ctx A<br>* NS group: no shock in Ctx A	Context B inputs<br>(AAV-DIO-ChR2)	Randomly selected<br>BA neurons	No difference in PPR (FC vs NS)<br>* No presynaptically-expressed synaptic potentiation in Context B vCA1 inputs to BA neurons
Suppl Fig. 10	Fos-Cre	Label: Context A (x3, Tam)<br>* FC group: dCFC in Ctx A<br>* NS group: no shock in Ctx A	Context A inputs<br>(AAV-DIO-ChR2)	Randomly selected<br>CeA neurons	No difference in AN (FC vs NS)<br>* No strengthening in Context A vCA1 inputs to CeA neurons
Suppl Fig. 11	Wild type	FC group: dCFC in Ctx A<br>NS group: no shock in Ctx A	Nonspecific inputs<br>(AAV-CaMKII-ChR2)	Randomly selected<br>BA neurons	No difference in AN, NMDAR EPSC decay rate, or PPR (FC vs NS)<br>* No strengthening in nonspecific vCA1 inputs to BA neurons
Suppl Fig. 12	Fos-Cre x LSL-tdT	Label 1: Context A (x2, Tam)<br>Label 2: CFC in Ctx A (Tam)	Context A inputs<br>(AAV-DIO-ChR2)	tdT+: BA fear neurons<br>tdT-: other BA neurons	AMPAR EPSC (tdT+) > AMPAR EPSC (tdT-)<br>No difference in NMDAR EPSC amplitude (FC vs NS)<br>No difference in feed-forward inhibition (FC vs NS)
Suppl Fig. 13	Fos-Cre x LSL-tdT	Label: Context A (x3, Tam)	AAV-DIO-ChR2<br>(Context A inputs)	tdT+ BA neurons<br>tdT- BA neurons	No difference in AN (tdT+ vs tdT-)<br>* No synaptic strengthening between vCA1 and BA neurons active during context exposure
Suppl Fig. 15	Fos-Cre x LSL-tdT	Label 1: Context A (x2, Tam)<br>Label 2: CFC in Ctx A (Tam, + SAL or MK-801)	Context A inputs<br>(AAV-DIO-ChR2)	tdT+: BA fear neurons<br>tdT-: other BA neurons	AN (tdT+) > AN (tdT-) in saline (SAL) control group<br>No difference in AN (tdT+ vs tdT-) in MK-801 group<br>* MK-801 prevents stregnthening of vCA1-BA pathway.

* Major conclusions are highlighted in red.

** Abbreviations: Tam (Tamoxifen), FC (fear conditioning), dCFC (discriminative contextual fear conditioning), Ctx (Context), NS (no shock), AN (AMPA/NMDA EPSC ratio), PPR (paired-pulse ratio) CFC (contextual fear conditioning), tdT (tdTomato), SAL (saline)
