# Supplementary material for: Encoding of contextual fear memory in hippocampal–amygdala circuit
Source: Nat Commun. 2020 Mar 13;11:1382. doi: 10.1038/s41467-020-15121-2 (PMC7069961; doi:10.1038/s41467-020-15121-2)
Supplement: Supplementary file 2 — Reporting Summary [file 41467_2020_15121_MOESM2_ESM.pdf]

nature research

Corresponding author(s): Jun-Hyeong Cho

Last updated by author(s): Jan 24, 2020

Reporting Summary

Nature Research wishes to improve the reproducibility of the work that we publish. This form provides structure for consistency and transparency in reporting. For further information on Nature Research policies, see Authors & Referees and the Editorial Policy Checklist .

Statistics

For all statistical analyses, confirm that the following items are present in the figure legend, table legend, main text, or Methods section.

n/a	Confirmed	
☐	☒	The exact sample size (*n*) for each experimental group/condition, given as a discrete number and unit of measurement
☐	☒	A statement on whether measurements were taken from distinct samples or whether the same sample was measured repeatedly
☐	☒	The statistical test(s) used AND whether they are one- or two-sided<br>*Only common tests should be described solely by name; describe more complex techniques in the Methods section.*
☐	☒	A description of all covariates tested
☐	☒	A description of any assumptions or corrections, such as tests of normality and adjustment for multiple comparisons
☐	☒	A full description of the statistical parameters including central tendency (e.g. means) or other basic estimates (e.g. regression coefficient) AND variation (e.g. standard deviation) or associated estimates of uncertainty (e.g. confidence intervals)
☐	☒	For null hypothesis testing, the test statistic (e.g. *F*, *t*, *r*) with confidence intervals, effect sizes, degrees of freedom and *P* value noted<br>*Give P values as exact values whenever suitable.*
☒	☐	For Bayesian analysis, information on the choice of priors and Markov chain Monte Carlo settings
☒	☐	For hierarchical and complex designs, identification of the appropriate level for tests and full reporting of outcomes
☒	☐	Estimates of effect sizes (e.g. Cohen's *d*, Pearson's *r*), indicating how they were calculated

Our web collection on statistics for biologists contains articles on many of the points above.

Software and code

Policy information about availability of computer code

Data collection	Ethovision XT 11 (Noldus) was used to collect data in behavioral studies. Clampex 10 (Molecular Devices) was used to obtain electrophysiological data.
Data analysis	Clampfit 10 (Molecular Devices) was used to analyze electrophysiological data. ImageJ 1.45s (NIH) was used to analyze confocal microscopic data. Imaris 9 Bitplane was used to count labeled cells. Statistical analysis was performed with Minitab 18 software (Minitab).

For manuscripts utilizing custom algorithms or software that are central to the research but not yet described in published literature, software must be made available to editors/reviewers. We strongly encourage code deposition in a community repository (e.g. GitHub). See the Nature Research guidelines for submitting code & software for further information.

Data

Policy information about availability of data

All manuscripts must include a data availability statement. This statement should provide the following information, where applicable:
- Accession codes, unique identifiers, or web links for publicly available datasets
- A list of figures that have associated raw data
- A description of any restrictions on data availability

All data reported in this study are available from the corresponding authors upon request. The source data underlying all Figures and Supplementary Figures are provided as **a** Source Data file.

Field-specific reporting

Please select the one below that is the best fit for your research. If you are not sure, read the appropriate sections before making your selection.

- [x] Life sciences
- [ ] Behavioural & social sciences
- [ ] Ecological, evolutionary & environmental sciences

For a reference copy of the document with all sections, see nature.com/documents/nr-reporting-summary-flat.pdf

Life sciences study design

All studies must disclose on these points even when the disclosure is negative.

Sample size	Using preliminary data and results from previous studies, we performed power analysis to determine sample size. Sample sizes are indicated in the legends of Figures and Supplementary Figures.
Data exclusions	No data was excluded from the study.
Replication	Micrographic images presented in some figures are representative ones from experiments repeated independently as indicated under 'Reproducibility' in the Methods section. Statistically significant results in critical electrophysiological experiments were replicated successfully.
Randomization	Mice were randomly assigned to behavioral groups.
Blinding	The investigators were blinded to groups for cell counting experiments. Blinding was not relevant to behavioral studies as freezing behavior was automatically scored with constant settings in Ethovision. Blinding was not necessary for electrophysiological experiments as recording data were obtained under constant experimental conditions.

Reporting for specific materials, systems and methods

We require information from authors about some types of materials, experimental systems and methods used in many studies. Here, indicate whether each material, system or method listed is relevant to your study. If you are not sure if a list item applies to your research, read the appropriate section before selecting a response.

Materials & experimental systems

n/a	Involved in the study	
☐	☒	Antibodies
☒	☐	Eukaryotic cell lines
☒	☐	Palaeontology
☐	☒	Animals and other organisms
☒	☐	Human research participants
☒	☐	Clinical data

Methods

n/a	Involved in the study	
☒	☐	ChIP-seq
☒	☐	Flow cytometry
☒	☐	MRI-based neuroimaging

Antibodies

Antibodies used	Polyclonal rabbit affinity purified anti-c-Fos antibody (Synaptic Systems, Cat# 226003)<br>Goat anti-rabbit IgG antibody-Alexa Fluor 647 (Thermo Fisher, Cat# A-21246)
Validation	The antibodies were validated by the manufacturers as below:<br>anti-c-Fos antibody (https://www.sysy.com/products/c-fos/facts-226003.php)<br>anti-rabbit IgG antibody (https://www.thermofisher.com/antibody/product/Goat-anti-Rabbit-IgG-H-L-Cross-Adsorbed-Secondary-Antibody-Polyclonal/A-21246)

Animals and other organisms

Policy information about studies involving animals; ARRIVE guidelines recommended for reporting animal research

Laboratory animals	C57BL6/J mice: Jackson Laboratory Stock # 000664<br>Fos-CreERT2 (+/-) mice: Jackson Laboratory Stock # 021882<br>Arc-CreERT2 (+/-) mice: Jackson Laboratory Stock # 022357<br>Fos-tTA/Fos-shGFP (+/-) mice: Jackson Laboratory Stock # 018306<br>Ai9 ROSA-LSL-tdTomato (+/+) mice: Jackson Laboratory Stock # 007909<br>GAD2-IRES-Cre (+/+) mice: Jackson Laboratory Stock # 010802<br>* These mice were singly housed in home cages on a 12-h light/dark cycle with food and water continuously available. The light

	cycle was from 8 AM to 8 PM. Temperature range was 22-24°C, and humidity range was 30-70 %. Six- to eight-week-old mice of both sexes underwent stereotaxic brain surgery.
Wild animals	No wild animal was used.
Field-collected samples	No field-collected sample was used.
Ethics oversight	All of the animal procedures were approved by the Institutional Animal Care and Use Committee of the University of California, Riverside.

Note that full information on the approval of the study protocol must also be provided in the manuscript.
